# Cost-Utility Analysis of the Integrated Care Models for the Management of Hypertension Patients: A Quasi-Experiment in Southwest Rural China

**DOI:** 10.3389/fpubh.2021.727829

**Published:** 2021-12-13

**Authors:** Xiatong Ke, Liang Zhang, Wenxi Tang

**Affiliations:** ^1^School of International Pharmaceutical Business, China Pharmaceutical University, Nanjing, China; ^2^Center for Pharmacoeconomics and Outcomes Research of China Pharmaceutical University, Nanjing, China; ^3^School of Medical and Health Management, Tongji Medical College of Huazhong University of Science and Technology, Wuhan, China; ^4^Research Center for Rural Health Services, Hubei Province Key Research Institute of Humanities and Social Sciences, Wuhan, China

**Keywords:** integrated care, hypertension management, cost-benefit analysis, Markov model, rural China

## Abstract

**Background:** Hypertension has become the second-leading risk factor for death worldwide. However, the fragmented three-level “county–township–village” medical and healthcare system in rural China cannot provide continuous, coordinated, and comprehensive health care for patients with hypertension, as a result of which rural China has a low rate of hypertension control. This study aimed to explore the costs and benefits of an integrated care model using three intervention modes—multidisciplinary teams (MDT), multi-institutional pathway (MIP), and system global budget and performance-based payments (SGB-P4P)—for hypertension management in rural China.

**Methods:** A Markov model with 1-year per cycle was adopted to simulate the lifetime medical costs and quality-adjusted life-years (QALYs) for patients. The interventions included Option 1 (MDT + MIP), Option 2 (MDT + MIP + SGB–P4P), and the Usual practice (usual care). We used the incremental cost-effectiveness ratio (ICER), net monetary benefit (NMB), and net health benefit (NHB) to make economic decisions and a 5% discount rate. One-way and probability sensitivity analyses were performed to test model robustness. Data on the blood pressure control rate, transition probability, utility, annual treatment costs, and project costs were from the community intervention trial (CMB-OC) project.

**Results:** Compared with the Usual practice, Option 1 yielded an additional 0.068 QALYs and an additional cost of $229.99, resulting in an ICER of $3,373.75/QALY, the NMB was –$120.97, and the NHB was −0.076 QALYs. Compared with the Usual practice, Option 2 yielded an additional 0.545 QALYs, and the cost decreased by $2,007.31, yielding an ICER of –$3,680.72/QALY. The NMB was $2,879.42, and the NHB was 1.801 QALYs. Compared with Option 1, Option 2 yielded an additional 0.477 QALYs, and the cost decreased by $2,237.30, so the ICER was –$4,688.50/QALY, the NMB was $3,000.40, and the NHB was 1.876 QALYs. The one-way sensitivity analysis showed that the most sensitive factors in the model were treatment cost of ESRD, human cost, and discount rate. The probability sensitivity analysis showed that when willingness to pay was $1,599.16/QALY, the cost-effectiveness probability of Option 1, Option 2, and the Usual practice was 0.008, 0.813, and 0.179, respectively.

**Conclusions:** The integrated care model with performance-based prepaid payments was the most beneficial intervention, whereas the general integrated care model (MDT + MIP) was not cost-effective. The integrated care model (MDT + MIP + SGB-P4P) was suggested for use in the community management of hypertension in rural China as a continuous, patient-centered care system to improve the efficiency of hypertension management.

## Introduction

Hypertension has become the second-leading risk factor for death globally next to cancer, accounting for 10.8 million deaths in 2019 (1). There were positive log-linear associations of blood pressure with cardiovascular disease (CVD); e.g., each 10 mmHg higher usual systolic blood pressure (SBP) was associated with a hazard ratio of 1.28, 1.18, and 1.17 for cardiovascular death, major coronary events, and ischemic stroke, respectively (2). In 2019, ischemic heart disease and stroke were the top-ranked causes of disability-adjusted life-years for both 50- to 74-year-olds and those aged 75 years and above (3). However, insufficient control of hypertension in rural China is common, resulting in substantial excess risks of CVD and increases in the disease burden and healthcare expenditures.

Different countries have different management models for hypertension control, such as Preferred Provider Organization (PPO), Health Maintenance Organization (HMO), and Point of Services (POS) in the United States; providing personalized health guidance for chronic patients and performing performance evaluations based on the completion of medical institution to provide economic rewards and punishments in Japan; changing people's behavior and guiding people to choose healthy lifestyles in Finland. Summarizing the hypertension management models in many countries, we found that the Chronic Care Model (CCM) based on community prevention and treatment was the most effective. In the CCM model, delivery system design requires a macroscopic design and reconstruction of the health system, which mainly includes three aspects: a multidisciplinary team, disease management, and planned follow-up. The provision of macro health services plays a decisive role in CCM. Without the support of an integrated and coordinated health system, the model cannot be effectively implemented.

The United States 4. Centers for Disease Control and Prevention (4) study found that the main reason for insufficient hypertension control was the lack of continuous and coordinated comprehensive services for patients with hypertension. As early as 1996, scholars pointed out that the fragmented system made it difficult to meet the needs of patients with hypertension for comprehensive and coordinated services (5). The healthcare delivery system is particularly fragmented in rural China, as it involves three levels of delivery at county, township, and village (6). The three-level healthcare delivery system is characterized by uncoordinated and inefficient care, resulting in the inefficient use of funds and an increase in the disease burden of patients (7). Thus, there is an urgent need to transform the healthcare delivery system from a dysfunctional and fragmented system to an integrated care system.

Several recent studies have shown that blood pressure control will be improved by using a multi-institutional, multidisciplinary team to provide patients with integrated care, including a comprehensive hypertension registry, development and sharing of performance metrics, evidence-based guidelines, and medical assistant visits for blood pressure measurement and single-pill combination pharmacotherapy (8–12). Crosson pointed out that the integrated care delivery system should adopt a prepayment system, as multiple institutions would conduct a “virtual integration” by signing contracts and forming teams to share patient resources and reduce management costs (13). VanLare et al. found that performance-based payments create incentives for physicians to improve health care and reduce costs (14).

To reform the fragmented healthcare delivery system in rural China, we examined relevant literature, undertook investigations of certain rural areas, and finally determined that the intervention package should be a combination of multidisciplinary team (MDT), multi-institutional pathway (MIP), and system global budget and performance-based payment (SGB-P4P) reform. To test out the effectiveness of the potential intervention package in rural China, we conducted a multi-town clustered randomized trial on hypertension management in Qianjiang district in Chongqing, a rural municipality in southwestern China. The integrated CCM of hypertension in China's Qianjiang county study showed success in lowering blood pressure, reducing rates of hospitalization, and reducing inpatient spending (15). In this paper, we aim to retrospectively quantify the costs and benefits of the integrated CCM of hypertension in rural China.

In this study, we used a Markov model to perform the cost-utility analyses, comparing Option 1 (MDT + MIP intervention), Option 2 (MDT + MIP + SGB-P4P intervention), and Usual practice (usual care) in the CMB–OC community intervention trial for hypertension management in rural China from the perspective of the healthcare system. Hereafter, we refer to this trial, which was entitled the “Study on the effectiveness and efficiency of rural health-care integration” (11–69) as the CMB-OC trial (16, 17).

## Methods

The data used in this study were mainly derived from the CMB–OC trial, which was conducted between July 2012 and December 2014 (16, 17). The trial was a quasi-randomized controlled experiment, which established a prospective dynamic cohort to observe the intervention process and effectiveness. Qianjiang in Chongqing Municipality was selected as the pilot city for the community intervention. According to the principle of stratified random sampling, Zhuoshui and Shihui were selected as the pilot towns for Option 2, Apengjiang and Jinxi as the pilot towns for Option 1, and Shijia and Fengjia as the control towns.

The sample areas were representative of the following: (1) The population size was moderate, and the level of economic development was backward, which was a typical rural area in China; (2) the incidence of chronic diseases was increasing day by day, and the management foundation was weak; (3) the family structure of elderly patients with chronic diseases is dominated by “empty nest families” and “broken-off families,” lacking social support. The representativeness of the sample area made the research conclusions generalizable.

This analysis applied the Patients–Intervention–Comparators–Outcomes–Setting (PICOS) principle, which describes the essential components of a health economic evaluation in order of importance. The PICOS principle has been successfully applied in multiple health economic evaluation tasks (18).

### Population

The objects of intervention in this study were consistent with those in the CMB–OC trial. The inclusion criteria for patients with hypertension were as follows: (1) patients who were registered for hypertension management at the health center of the pilot town; (2) patients who continued to participate in the new rural cooperative medical scheme; and (3) patients who were permanent residents, that is, who lived in the town for at least 6 months each year or throughout the year in the case of patients undergoing level 3 hypertension management.

The exclusion criteria were as follows: (1) patients with severe visual and hearing impairments who were unable to communicate normally; (2) patients with tumors who had received radiotherapy or chemotherapy in the past 6 months; (3) patients who had mild or severe mental illness; (4) patients who had secondary hypertension; and (5) patients who had participated in other clinical research trials and taken associated medication within the past month.

At the time of the baseline survey, all patients signed an informed consent form, and the trial received approval from the Medical Ethics Committee of Tongji Medical College, Huazhong University of Science and Technology (Wuhan, China). The trial was registered in the Chinese Clinical Trial Registry (ChiCTR-OOR-14005563).

### Interventions of Interest

As stated above, the three experimental groups tested were Option 1 (MDT + MIP), Option 2 (MDP + MIP + SGB-P4P), and the Usual practice (control group), who received their usual care. The specific content of each intervention was as follows.

(1) MDT involved the following steps. First, 15 physicians were selected from the endocrinology, neurology, and cardiology departments in each of two district-level hospitals as the top level, technical experts of the team. Second, two clinicians (responsible for outpatients and hospitalization), three chronic disease service personnel (responsible for screening, follow-up, and monitoring of patients with hypertension), and one project coordinator were selected from the health centers in each sample town. Third, village doctors in each sample town were included as the bottom level of the team to assist with chronic disease follow-ups in the community and basic medical treatments.
(2) The MIP formulates the treatment approaches and process management methods for a certain disease to integrate all services, such as screening, diagnosis, treatment, rehabilitation, and community management, into continuous and coordinated services. The pathway consisted of three parts: (a) routine community health care under general conditions, including screening, monitoring, and follow-up; (b) continuous diagnosis and treatment under disease conditions, including medical status assessment, continuous treatment, variation analysis, and rehabilitation treatment; and (c) process management, including communication between physicians, referrals, document delivery, and joint follow-up.
(3) The SGB–P4P component of the intervention mainly aimed to stimulate the service personnel from different institutions to make a reasonable diagnosis and determine treatment based on team work and patient benefit. The specific steps included (a) historical cost estimation and budgeting, and allocation of total budget to the public account of the MDT on a monthly basis; (b) determination of the inclusion/exclusion criteria for cost estimation; (c) determination of the performance indicators; (d) consulting, negotiating, and contracting; (e) prepayment and budget allocation; (f) supervision, evaluation, audit, and feedback; (g) financial settlement and balance allocation; and (h) a rolling budget for the next fiscal year.

The MDT + MIP personnel training for Option 1 and Option 2 was carried out from January to May 2013. The training mainly involved the implementation of coordinated team learning, table manipulation, and process operations. The SGB-P4P estimation in Option 2 started from August 2012. After three rounds of negotiations with district hospitals during the period from March to May 2013, the SGB-P4P reform pilot was officially implemented from May 31, 2013. The specific time frame for the interventions in Option 1 and Option 2 was from June 2013 to December 2014.

### Outcomes

In this study, the primary outcome was the incremental cost-effectiveness ratio (ICER), measured as the difference (Δ) in simulated costs divided by the difference in simulated effectiveness [measured in quality-adjusted life-years (QALYs)]. Secondary outcomes included the net monetary benefit (NMB), net health benefit (NHB), mean total costs, and mean total QALYs gained.

### Economic Evaluation

#### Markov Model Structure

**Figure 1:**
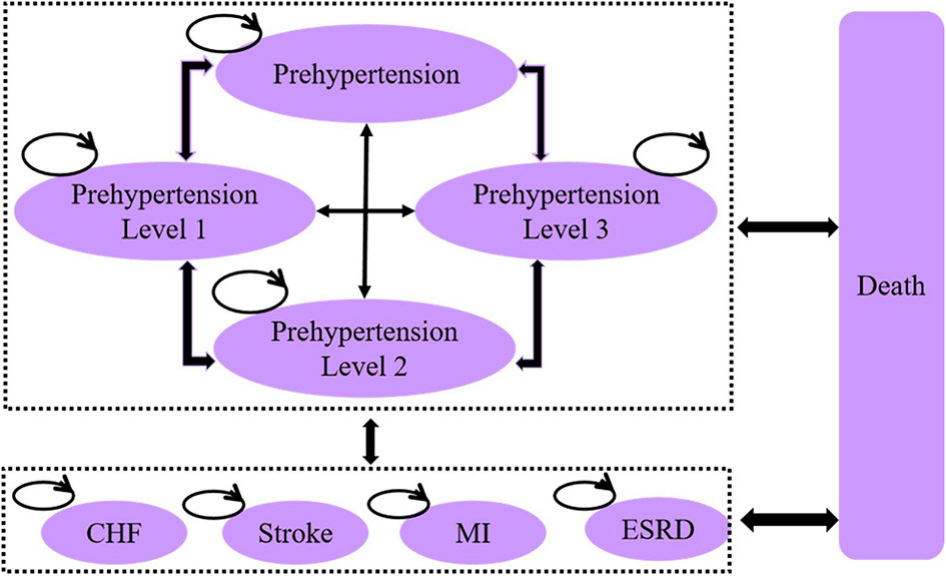
Schematic diagram showing health-state transition in the Markov model for hypertension. (1) Prehypertension: systolic blood pressure (SBP) = 120–139, diastolic blood pressure (DBP) = 80–89; (2) Hypertension-L1: SBP = 140–159, DBP = 90–99; (3) Hypertension-L2: SBP = 160–179, DBP = 100–109; (4) Hypertension-L3: SBP ≥ 180, DBP ≥ 110; (5) MI: myocardial infarction; (6) CHF: congestive heart failure; (7) ESRD: end-stage renal disease.

Based on the classification of hypertension, natural clinical outcomes of blood pressure, and existing model structures in the literature (19–21), we constructed a Markov model structure for hypertension (Figure 1) using Microsoft Excel 2019 (Microsoft Corp., Redmond, WA, United States). The constructed model mainly consisted of nine health states: prehypertension, hypertension level 1 (L1), hypertension level 2 (L2), hypertension level 3 (L3), CVD represented by stroke, coronary heart disease (CHD) represented by myocardial infarction (MI) and congestive heart failure (CHF), other hypertensive diseases represented by end-stage renal disease (ESRD), and death. The health states of all hypertension levels can be transferred into each other. Meanwhile, all levels of hypertension involve a certain probability of transitioning to other health states, including MI, stroke, CHF, ESRD, and death.

#### Study Perspective

Because an integrated CCM requires the coordination of multilevel medical institutions, we selected the health system perspective and estimated the impact of the model on healthcare expenditures in the entire healthcare system.

#### Key Assumptions

(1) First, we assumed that the key factors underpinning the fragmented system and discontinuous services in the sample area were related to the lack of health human resources in rural areas of China, the absence of standardized pathways for cooperative behavior among medical personnel at all levels of medical institutions, and the lack of incentives motivating physicians to coordinate. We also assumed that the interventions used in this study can improve the situation in the above three aspects to promote service integration.
(2) We assumed that the discount rate for model parameters, such as costs, utilities, and benefits, was 5%.
(3) We assumed that the treatment costs of associated diseases caused by hypertension were close to the mean treatment costs in China's general public hospitals.
(4) We assumed that in the case of no incidences of hypertension or related diseases, mortality was equivalent to the natural mortality.
(5) Finally, we assumed that the patients cannot return to the normotension state and that the best state that they can achieve was the prehypertension state.

#### Study Time Horizon

Hypertension is a typical chronic disease, so the study period was set to the patients' lifetime. Based on the results of model operation, we found that 99.2% of the patients died after running 65 cycles, with 1-year per cycle, namely, after 65 years. Accordingly, the time horizon of this study was set to 65 years, and the cycle length was 1 year.

### Parameter Estimation

#### Effectiveness

In the CMB–OC trial, the health outcome indicators of patients included blood pressure, blood pressure control rate, and quality of life. We used the blood pressure control rate as an effectiveness indicator. After the start of the trial, blood pressure measurement was carried out at the time of follow-up appointments every 2 months, and the follow-up period was from August 2012 to June 2014. A total of 12 blood pressure measurements were taken. According to the SPRINT trial study, lowering the standard of normal SBP to 120 mmHg, rather than the clinical standard of 140 mmHg, can reduce CHD events by 25% (22). Therefore, only SBP below 120 mmHg was regarded as blood pressure control in this study. The blood pressure control rate was calculated based on data from 12 follow-ups as part of the CMB–OC trial project. The mean blood pressure control rates for Option 1, Option 2, and Usual practice are listed in Table 1.

**Table 1:** Blood pressure control rate, sensitivity analysis range, and parameter distribution.

**Parameter name**	**Parameter description**	**Mean value**	**Sensitivity analysis range**	**Parameter distribution**	**Source**
SSCR	Blood pressure control rate of G1	0.62	(0.33, 0.92)	Beta	CMB-OC project
DSCR	Blood pressure control rate of G2	0.69	(0.42, 1.00)	Beta	
PSCR	Blood pressure control rate of control group	0.61	(0.25, 0.83)	Beta	
P0.5	Proportion of prehypertension	0.32	/	/	
P1	Proportion of hypertension level 1	0.53	/	/	
P2	Proportion of hypertension level 2	0.12	/	/	
P3	Proportion of hypertension level 3	0.03	/	/	

*G1, Group 1 (MDT + MIP intervention); G2, Group 2 (MDP + MIP + SGB–P4P intervention); control group (usual care). Similar notation is used hereafter*.

#### Utility

In the CMB–OC trial, the quality of life of patients with hypertension was measured using the Short Form (SF)-36 scale, which collected eight dimensional scores for the patients, namely, physiological function (PF), role physical (RP), bodily pain (BP), general health (GH), vitality (VT), social function (SF), role emotional (RE), and mental health (MH). The National Institute for Clinical Excellence (NICE) Guide to the technology appraisal process recommends that the EQ-5D scale, a measurement tool based on patient preference, should be used to calculate the utilities for economic evaluation (23). Accordingly, we mapped the patients' eight dimensional scores collected by the SF-36 scale to EQ-5D using a publicly published and validated mapping model. According to the Health Economics Research center (HERC) database of mapping studies (24), as of October 2020, there were 413 articles on utility mapping, including six articles (25–28) on mapping SF-36 to EQ-5D. Taking into account the study population, disease, sample size, and goodness-of-model fit, we selected Ara and Brazier's (25) Model 1 as the bridge to map SF-36 to EQ-5D. Of the seven models in Ara and Brazier (25), we concluded that Model 1 produced the most accurate results, although all had advantages and disadvantages. The model is expressed by the following equation:


$$\begin{array}{l} EQ-5D=0.03256+0.0037{\;}^{*}\;PF+0.00111{\;}^{*}\;SF-0.00024\\ \;{\;}^{*}\;RP+0.00024{\;}^{*}\;RE+0.00256{\;}^{*}\;MH-0.00063\\ {\;}^{*}\;VT+0.00286{\;}^{*}\;BP+0.00052{\;}^{*}\;GH \end{array}$$

The advantage of Model 1 is that EQ-5D is significantly correlated with all eight dimensions of SF-36. Except for RP and VT, all the other dimensions are positively correlated with EQ-5D. The disadvantage is that the model overpredicts when the EQ-5D score is lower than 0.5 (25).

Because the utility values for the health states of MI, CHF, stroke, and ESRD were missing in the trial data, we used the utility values from clinical data and literature on China and other countries. The utility of each health state is summarized in Table 2.

**Table 2:** Utility, range, parameter distribution, and reference sources for different health states.

**Parameter name**	**Parameter description**	**Basic value**	**95% confidence interval**	**Parameter distribution**	**Reference source**
U_nor	Utility of normotension	0.698375	(0.4689, 0.9278)	Beta	SF-36 mapping to EQ-5D
U_pre	Utility of prehypertension	0.651375	(0.6066, 0.6962)	Beta	
U_1	Utility of hypertension-L1	0.65525	(0.6057, 0.7048)	Beta	
U_2	Utility of hypertension-L2	0.683	(0.5595, 0.8065)	Beta	
U_3	Utility of hypertension-L3	0.669	(0.5153, 0.8227)	Beta	
U_MI	Utility of MI	0.684	(0.6165, 0.7524)	Beta	PLATO data (29)
U_Stroke	Utility of stroke	0.605	(0.5445, 0.6655)	Beta	
U_CHF	Utility of CHF	0.64	(0.6086, 0.6714)	Beta	van Stel and Buskens (30)
U_ESRD	Utility of ESRD	0.60	(0.3900, 0.8100)	Beta	Yang et al. (31)

*Please refer to Table 1 for explanations of the abbreviations*.

#### Transition Probabilities and Mortality

The data on transition probabilities among prehypertension, hypertension-L1, hypertension-L2, and hypertension-L3 were derived from the follow-up data of the CMB-OC trial. There was a total of six steps. In the first three steps, we determined, first, the level of hypertension based on the blood pressure values of patients at baseline; second, the level of hypertension based on the blood pressure values of patients at the 12th follow-up after the intervention; and third, the change in the level of hypertension from baseline to the end of follow-up for each patient. For the fourth and fifth steps, we calculated the study probability (Table 3) and the study rate as–(1/t) * LN (1–study probability). For the sixth step, we calculated the 1-year probability, namely, the transition probability, as 1–EXP (–study rate).

**Table 3:** Original transition probabilities among different levels of hypertension during the intervention period of the CMB–OC trial.

**Parameter**	**Parameter description**	**Transition**	**Study**
**name**		**(*n*)**	**probability**
			**(%)**
0_0.5	From normotension to prehypertension	5	0.0040
0_1	From normotension to hypertension-L1	5	0.0040
0_2	From normotension to hypertension-L2	1	0.0008
0.5_0	From prehypertension to normotension	12	0.0096
0.5_0.5	From prehypertension to prehypertension	269	0.2155
0.5_1	From prehypertension to hypertension-L1	101	0.0809
0.5_2	From prehypertension to hypertension-L2	14	0.0112
0.5_3	From prehypertension to hypertension-L3	4	0.0032
1_0	From hypertension-L1 to normotension	17	0.0136
1_0.5	From hypertension-L1 to prehypertension	383	0.3069
1_1	From hypertension-L1 to hypertension-L1	234	0.1875
1_2	From hypertension-L1 to hypertension-L2	18	0.0144
1_3	From hypertension-L1 to hypertension-L3	4	0.0032
2_0	From hypertension-L2 to normotension	6	0.0048
2_0.5	From hypertension-L2 to prehypertension	56	0.0449
2_1	From hypertension-L2 to hypertension-L1	71	0.0569
2_2	From hypertension-L2 to hypertension-L2	14	0.0112
2_3	From hypertension-L2 to hypertension-L3	2	0.0016
3_0.5	From hypertension-L3 to prehypertension	15	0.0120
3_1	From hypertension-L3 to hypertension-L1	15	0.0120
3_2	From hypertension-L3 to hypertension-L2	2	0.0016
Sum		1248	100%

The transition probabilities from different levels of hypertension to MI, CHF, stroke, ESRD, and death were obtained by searching relevant data from the literature in China and other countries, and calculated using the transition probability equation above. The transition probabilities among different states are summarized in Table 4.

**Table 4:** Transition probabilities among different health states, sensitivity analysis range, parameter distribution, and sources.

**Parameter name**	**Parameter description**	**Basic value**	**Sensitivity analysis range**	**Parameter distribution**	**Reference source**
p0.5_1	From prehypertension to hypertension-L1	0.0413	(0.0330, 0.0496)	Beta	Calculated based on data from CMB-OC project
p0.5_2	From prehypertension to hypertension-L2	0.0056	(0.0045, 0.0067)	Beta	
p0.5_3	From prehypertension to hypertension-L3	0.0016	(0.0013, 0.0019)	Beta	
p1_0.5	From hypertension-L1 to prehypertension	0.1675	(0.01340, 0.2010)	Beta	
p1_2	From hypertension-L1 to hypertension-L2	0.0072	(0.0058, 0.0086)	Beta	
p1_3	From hypertension-L1 to hypertension-L3	0.0016	(0.0013, 0.0019)	Beta	
p2_0.5	From hypertension-L2 to prehypertension	0.0227	(0.0182, 0.0272)	Beta	
p2_1	From hypertension-L2 to hypertension-L1	0.0289	(0.0231, 0.0347)	Beta	
p2_3	From hypertension-L2 to hypertension-L3	0.0008	(0.0006, 0.0010)	Beta	
p3_0.5	From hypertension-L3 to prehypertension	0.0060	(0.0048, 0.0072)	Beta	
p3_1	From hypertension-L3 to hypertension-L1	0.0060	(0.0048, 0.0072)	Beta	
p3_2	From hypertension-L3 to hypertension-L2	0.0008	(0.006, 0.0010)	Beta	
p0.5_MI	From prehypertension to MI	0.0014	(0.0012, 0.0017)	Beta	Gu et al. (32)
P1_MI	From hypertension-L1 to MI	0.0022	(0.0018, 0.0027)	Beta	
P2_MI	From hypertension-L2 to MI	0.0035	(0.0028, 0.0042)	Beta	
P3_MI	From hypertension-L3 to MI	0.0035	(0.0028, 0.0042)	Beta	
P0.5_CHF	From prehypertension to CHF	0.0002	(0.0002, 0.0003)	Beta	
P1_CHF	From hypertension-L1 to CHF	0.0003	(0.0003, 0.0004)	Beta	
P2_CHF	From hypertension-L2 to CHF	0.0004	(0.0003, 0.0005)	Beta	
P3_CHF	From hypertension-L3 to CHF	0.0004	(0.0003, 0.0005)	Beta	
P0.5_Stroke	From prehypertension to stroke	0.0011	(0.0009, 0.0013)	Beta	
P1_Stroke	From hypertension-L1 to stroke	0.0020	(0.0016, 0.0024)	Beta	
P2_Stroke	From hypertension-L2 to stroke	0.0035	(0.0028, 0.0042)	Beta	
P3_Stroke	From hypertension-L3 to stroke	0.0035	(0.0028, 0.0042)	Beta	
pMI_Death	From MI to death	0.0049	(0.00390, 00446)	Beta	
pCHF_Death	From CHF to death	0.0007	(0.00053, 0.00076)	Beta	
pStroke_Death	From stroke to death	0.0040	(0.0032, 0.0048)	Beta	
P1_ESRD	From hypertension-L1 to ESRD	0.0152	(0.0121, 0.0182)	Beta	Zhou (33)
P2_ESRD	From hypertension-L2 to ESRD	0.0078	(0.0063, 0.0094)	Beta	
P3_ESRD	From hypertension-L3 to ESRD	0.0017	(0.0014, 0.002)	Beta	
pESRD_Death	From ESRD to death	0.0552	(0.0442, 0.0662)	Beta	Zhang et al. (34)

The natural mortality of each health state was a time-dependent variable, and the data were derived from the Sixth National Population Census in China conducted in 2010 (Table 5).

**Table 5:** Natural mortality among people over 35 years of age in China.

**Age**	**Index**	**Deaths**
35–39	35	0.0012
40–44	40	0.0018
45–49	45	0.0026
50–54	50	0.0042
55–59	55	0.0062
60–64	60	0.0103
65–69	65	0.0172
70–74	70	0.0306
75–79	75	0.0495
80–84	80	0.0848
85–89	85	0.1274
90–94	90	0.1908
95–99	95	0.2171
100 and over	100	0.4543

#### Costs

##### Project Costs

The calculation of project costs involved two parts: first, the additional labor costs, and second, project expenditures added by implementing the integrated CCM. The project expenditures mainly comprised conference, printing, hospitality, and transportation expenses associated with the implementation of the project. The human capital approach was used to estimate the salaries corresponding to the working time of personnel in the project, namely, physicians, chronic care personnel, and managers. Their increased work time was derived from the records of the CMB–OC project. According to the National Bureau of Statistics of China (35), the mean annual salary of employed healthcare and social care workers is $17,979.40. Assuming that MDT and MIP members work for 250 days per year (365 days less statutory holidays) and 8 h per day, we have:


$$\begin{array}{l} Salary\;of\;MDT\;and\;MIP\;members\\ \;=\;(Mean\;annual\;salary\;of\;healthcare\;and\;social\;workers\\ \;employed\;÷\;Number\;of\;working\;days\;÷\;8\;working\\ \;hours\;per\;day)\;\times \;Additional\;working\;time \end{array}$$

##### Treatment Costs for Different Health States

In this study, the treatment costs for the health states of different hypertension levels were obtained from the survey data of the CMB-OC project. The treatment costs for the health states of MI, CHF, stroke, and ESRD were derived from National Health Commission Health (36) and the relevant literature.

Data on the project expenditures and treatment costs for different health states were recorded in 2013 and 2014, respectively. Considering inflation and the time value of money, we calculated the costs at an inflation rate of 2.5% (37), with 2021 as the base year. For certain parameters, the range of treatment costs for each stage of hypertension could not be found; in such cases, we conducted sensitivity analyses for fluctuations of ±20%.

All cost parameters in the study were converted using the exchange rate for Chinese yuan (CNY, ¥) and US dollars (USD, $) on June 2, 2021 (¥1 = $0.156).

### Willingness to Pay

In this study, WTP refers to the price that people are willing to pay to maintain a healthy state. The lack of official thresholds for different diseases has led to increased uncertainties in the main results of economic evaluations (38). Hypertension is an important pathogenic factor of CHD and CVD. Therefore, the measurement of WTP for hypertension should not be limited to the cost of treatment for hypertension; the treatment costs of MI, stroke, and other CHD and CVD should be taken into account. According to the results of our literature search, there remains a lack of WTP data for hypertension and associated CHD and CVD. Considering factors such as population characteristics and the economic development level, we adopted the results of Hideo (2005) in Japan (39) and used a mean WTP for hypertension treatment of $75.03/month. The mean WTP for 2021 transformed using the official consumer price index was $1,599.16/year.

To transform the primary outcome to NMB and NHB, the WTP thresholds were applied to:


$$NMB\;=\;WTP^{*}(Change\;in\;QALYs)-(Change\;in\;Cost)$$

The NHB was calculated by subtracting the costs divided by the WTP from the health gain or loss, as follows:


NHB = (Change in QALYs)−((Change in Cost)/WTP)

## Sensitivity Analysis

Deterministic and probabilistic sensitivity analyses were used to determine whether the results were robust. The deterministic sensitivity analysis was conducted using a one-way sensitivity analysis, under which we changed the value of only one key variable at a time and then examined the change in the results. The probabilistic sensitivity analysis was conducted based on the distribution of different parameters using a second-order Monte Carlo simulation, and the results were represented by the cost-effectiveness acceptability curve. We discussed both sensitivity analyses below.

### One-Way Sensitivity Analysis

The range of variation in the parameters in the one-way sensitivity analysis is shown in Tables 1–6. Generally, the range of variations for the model parameters was based on the 95% confidence interval, the maximum value, and the minimum value of parameter estimates. For parameters that lacked reference data, such as treatment costs for different levels of hypertension, we allowed their range to fluctuate up and down by 20%. The discount rates of costs and utilities ranged from 0 to 8%.

**Table 6:** Annual costs of intervention measures and different health states (US dollars).

**Parameter name**	**Parameter description**	**Basic value**	**Sensitivity analysis range**	**Parameter distribution**	**Reference source**
cphuman	Human costs of control	$346.10	($258.89, $433.32)	Gamma	Calculated from trial data
cshuman	Human costs of G1	$24,469.97	($15,798.43, $33,141.50)	Gamma	
cdhuman	Human costs of G2	$11,938.33	($2,519.22, $21,357.40)	Gamma	
cpprogram	Project costs of control	$14.72	($0, $55.52)	Gamma	
csprogram	Project costs of G1	$1,278.47	($204.85, $2,352.09)	Gamma	
cdproagram	Project costs of G1	$699.40	($648.90, $749.90)	Gamma	
cprehy	Treatment cost of prehypertension	$140.69	($112.55, $168.83)	Gamma	
chy1	Treatment cost of hypertension-L1	$150.87	($120.70, $181.05)	Gamma	
chy2	Treatment cost of hypertension-L2	$187.08	($149.66, $224.49)	Gamma	
chy3	Treatment cost of hypertension-L3	$468.15	($374.52, $561.78)	Gamma	
cMI	Treatment cost of MI	$4,953.07	($3,962.45, $5,943.68)	Gamma	National Health Commission Health (36)
cCHF	Treatment cost of CHF	$1,754.30	($1,403.44, $2,105.16)	Gamma	
cStroke	Treatment cost of stroke	$1,974.68	($1,579.74, $2,369.61)	Gamma	
cESRD	Treatment cost of ESRD	$19,406.36	($15,525.09, $23,287.63)	Gamma	Wang et al. (40)

### Probabilistic Sensitivity Analysis

The probabilistic sensitivity analysis was conducted using a second-order Monte Carlo simulation with 1,000 iterations. The distribution of model parameters is summarized in Tables 1–6. Because the costs are positive and may have a long tail (i.e., a non-normal distribution), we assumed that the cost input parameters obey a Gamma distribution. Blood pressure control rates, transition probabilities, and utilities ranged between 0 and 1, so they were assumed to obey a beta distribution.

## Results

### Population Characteristics

According to the inclusion criteria, the sample size was 1,640 patients with hypertension. However, based on patients with hypertension who were actually surveyed at baseline in July 2012, the sample size reduced to 1,425. Then, because some patients withdrew during the follow-up period, by the time of the survey at the end of the study, the sample size was 1,245 patients with hypertension. Accordingly, the rate of loss for follow-up was 12.63%.

In the baseline period, the mean age of all patients was 67.2 years old (SD = 10.98), with ages ranging from 20 to 108 years old. The proportion of older people over 65 years was 61.3%, and the male-to-female ratio was 0.8:1. In terms of education levels, 36.6% of the patients received no education and 46.5% only attended elementary school. With regard to the family structure, 15.2% of the patients lived alone, 34.3% lived with their spouse only, and 48.4% lived with their children and spouse. The baseline characteristics of the patients indicate the generally high age, low education levels, and insufficient family support of patients with hypertension in rural China. The baseline characteristics of Option 1, Option 2, and Usual practice patients are summarized in Table 7 (15).

**Table 7:** Basic characteristics of patients with hypertension in different groups.

**Variables**	**Group 1**	**Group 2**	**Control**
Age	64.5	66.5	65.5
Female, %	51.9	54.2	55.9
**Family Structures, %**			
Living alone	14.4	16.0	14.6
Living with spouse only	34.3	32.8	36.9
Living with kids only	13.4	17.6	17.3
Living with both spouse and kids	35.8	32.8	28.8
Other family structure	2.1	0.8	2.4
**Education, %**			
No education	32.4	37.2	39.6
Attend elementary school	45.8	48.5	44.4
Attend high school or expenditure	21.9	14.3	16.1

### Base Case Analysis

The Markov model constructed using Microsoft Excel 2019 was applied to analyze the costs and benefits of the interventions for the management of patients with hypertension for Option 1 (MDT + MIP), Option 2 (MDP + MIP + SGB-P4P), and Usual practice (usual care) (Table 8). The results were as follows. (1) Compared with the Usual practice, Option 1's per-capita costs increased by $229.99 and resulted in an additional 0.068 QALYs, yielding an ICER of $3,373.75/QALY. The NMB was –$120.97, and the NHB was −0.076 QALYs. (2) Compared with the Usual practice, Option 2's per-capita costs fell by $2,007.31 and QALYs increased by 0.545. The resulting ICER was –$3,680.72/QALY, the NMB was $2,879.42, and the NHB was 1.801 QALYs. (3) Compared with Option 1, Option 2's per-capita costs fell by $2,237.30 and QALYs increased by 0.477, yielding an ICER of –$4,688.50, an NMB of $3,000.40, and an NHB of 1.876 QALYs.

**Table 8:** Basic results of the costs and benefits of different interventions.

**Program**	**Cost ($)**	**Years of life gained (LYGs)**	**Quality-Adjusted life years (QALYs)**
Option 1	$11,229.54	39.92488	15.331
Option 2	$8,992.24	42.1859	15.808
Usual practice	$10,999.55	41.8530	15.263
**Comparator**	**Option 1 vs. Usual practice**	**Option 2 vs. Usual practice**	**Option 2 vs. Option 1**
Incremental cost ($)	$229.99	–$2,007.31	–$2,237.30
Incremental effectiveness (QALYs)	0.068	0.545	0.477
ICER ($/QALYs)	$3,373.75	–$3,680.72	–$4,688.50
NMB ($)	–$120.97	$2,879.42	$3,000.40
NHB (QALYs)	−0.075648706	1.801	1.876
Decision making	G1 is the disadvantageous program, and G2 is the advantageous program		

### Sensitivity Analysis

**Figure 2:**
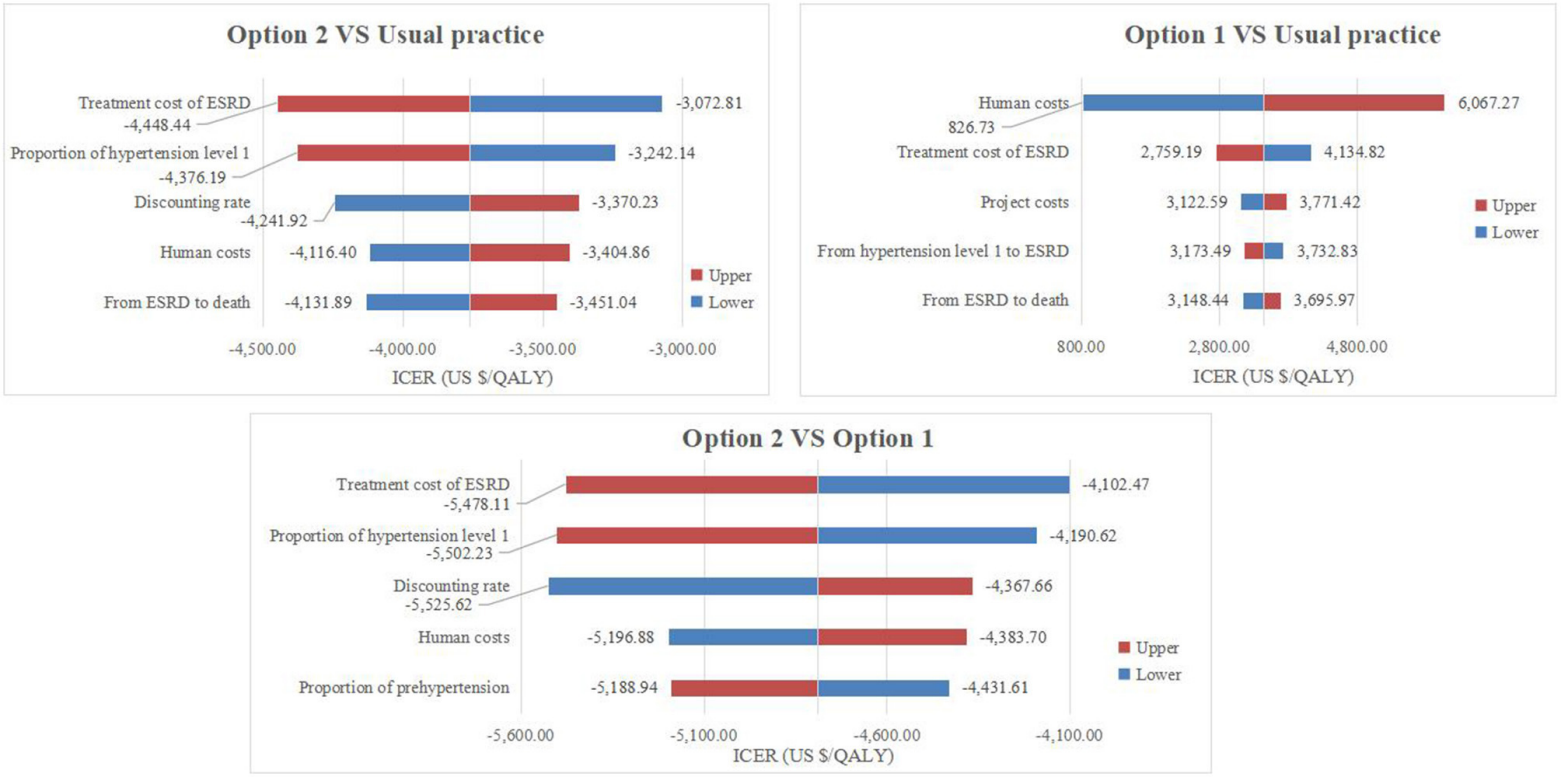
Tornado diagrams of the one-way sensitivity analysis.

The results of the one-way sensitivity analysis are shown in Figure 2. Among the model parameters, factors such as treatment cost of ESRD, human costs, and the discount rate exhibited a greater impact on the results. When the treatment cost for ESRD of Option 2 fluctuated by ±20%, the ICER was –$4,448.44/QALY and –$3,072.81, respectively. The one-way sensitivity analysis revealed that the model results were robust.

**Figure 3:**
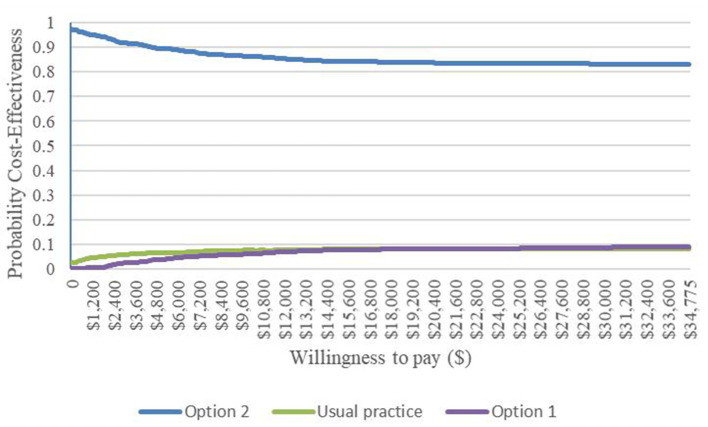
Cost-effectiveness acceptance curve.

The cost-effectiveness acceptability curve for the probabilistic sensitivity analysis is presented in Figure 3, which showed the probability that the treatment program was cost-effective under different WTP values. The probabilities of costs effectiveness for Option 1 (MDT + MIP), Option 2 (MDT + MIP + SGB-P4P), and the Usual practice were 0.008, 0.813, and 0.179, respectively, at the WTP threshold of $1,599.16/QALY. The Option 2 was always more cost-effective than the other option interventions at different WTP thresholds. The results of the probabilistic sensitivity analysis were consistent with the results of the basic analysis, indicating that these results were robust.

## Discussion

We studied the cost-effectiveness of integrated care model interventions, including Option 1 (MDT + MIP), Option 2 (MDT + MIP + SGB-P4P), and Usual practice for the management of patients with hypertension in rural China using the Markov model. We found that the Option 1 (MDT+MIP) was not cost-effective and the Option 2 (MDT + MIP + SGB-P4P) was more cost-effective.

In Option 1, the intervention was implemented through the general MDT + MIP integrated care model, which increased intervention costs but did not markedly improve QALYs among the patients compared with the control group. Possible reasons why the general MDT + MIP integrated care model is not cost-effective for the management of hypertension in rural China are as follows. First, like many service integration models in China that focus on institutional alliance, the incentives and support for clinicians from large hospitals to coordinate are insufficient in the context of the interdisciplinary teams within large hospitals and primary healthcare institutions. Second, relative to the increase in labor costs, the time for health benefits is long, and the improvement in efficiency is limited.

In Option 2, the intervention with payment method reform can improve the efficiency of coordination and communication between different levels of medical institutions through the group's internal benefit-sharing mechanisms, which, in turn, reduces the cost of delivering care. A previous study has shown that the SGB-P4P method has an incentive effect on vertical coordination behaviors that is 12 times greater than the incentive effect of the general MDT + MIP integrated care model (16). The potential reasons why the integrated care model with payment method reform is more cost-effective than the general integrated care model include the following: (1) SGB-P4P is factorable for incentivizing the personnel to achieve efficient referral communication between the different levels of medical institutions, and improving the vertical coordination and trust or familiarity of medical personnel between district- and township-level medical institutions, which in turn reduces the working time and intervention costs (41). For instance, Liu et al. (42) found that following the implementation of a global budget payment system, the expenditures of a healthcare insurance fund decreased and the ratio of healthcare insurance expenditures between the three-level county–township–village medical institutions changed, resulting in the saving of medical resources and more continuous delivery of services. (2) Interventions with changes in the payment method play a pronounced role in improving patients' self-perceived health (16, 43), which greatly enhances the effectiveness of such intervention projects. Therefore, the integrated care model with payment method reform achieves larger benefits at lower cost than does the general MDT + MIP integrated care model.

Existing studies have demonstrated that the MDT + MIP integrated care model accompanied by the SGB–P4P payment method reform has a major impact on the health system (44–46). The integration requires the vertical coordination of all levels of medical institutions. If an intervention relies only on reforms to the team and the MIP, it cannot markedly enhance the level of coordination. Therefore, it is imperative to introduce the incentive mechanisms associated with the payment method reform. In summary, the functions of the three intervention measures are as follows: MDT reform provides health-sector human resources, MIP reform supplies standardized pathways of diagnosis, treatment, referral, and communication for medical personnel, while SGB-P4P is an effective incentive method to drive the entire system to operate in a continuous, coordinated, and efficient manner.

In China, practical examples of integrated care models with SGB-P4P payment methods include Shenzhen Luohu Hospital Group (47, 48) and Shanghai Changning District General Practitioner Care Model (49). In Shenzhen, the Luohu Medical Group implements a health-oriented capitation payment system. The principle of the global budget management is summarized by the following statement: “scale payments keep the surplus for self-use, make up for over-expenditure, and share the costs reasonably.” An integrated healthcare delivery system is formed to ensure systematic, integrated, and continuous delivery of healthcare services for patients (50). Similarly, the General Practitioner Care Model in Changning District, Shanghai, combines a number of contracted services and implements a capitation payment system. Furthermore, it establishes a service-oriented distribution mechanism to reasonably determine the income structure of general practitioners, which ensures that the practitioners provide patients with continuous services in a reasonable, orderly, and standardized manner (51).

This study had some limitations, as follows. (1) Because the trial data only contained eight dimensional scores measured by the SF-36 scale, we mapped the SF-36 scores to EQ-5D through a mapping model based on the British population. Despite the large sample size, wide universality, and favorable extrapolation effect of the model, the utilities obtained might be biased. (2) The data sources of all the model parameters were not completely identical. If the data were missing in the CMB-OC project, we obtained the parameter values from data from other trials and relevant literature. For instance, the transition probabilities, utilities, and costs of health states, including MI, stroke, CHF, and ESRD, were derived from Plato data (29), relevant literature, and China's Health Statistical Yearbook. To address the bias that the different data sources might cause in the results, we conducted a parameter uncertainty analysis. (3) In the one-way sensitivity analysis, when the reference ranges (95% confidence interval, maximum value, and minimum value) of the parameters could not be found, the variation range was set to ±20% subjectively.

Despite these limitations, the study has a number of innovative features that distinguish it from the existing literature, as follows. (1) The Markov model has rarely been used to evaluate public healthcare interventions. We used the Markov model to predict and analyze the long-term cost-effectiveness of integrated care for the management of patients with hypertension, and the results provide an evidence-based reference for integrated care to improve the efficiency of the healthcare delivery system. (2) In this study, we selected the intervention programs according to local conditions and designed an integrated care model combining MDT, MIP, and SGB-P4P. The study design has compensated for the factors missing from the healthcare delivery system for the management of chronic diseases in China, including multi-team human resources, standardized pathways, and incentive measures. (3) The community intervention trial that we conducted was a long-term follow-up controlled cohort study over a period of ~3 years (2012–2014). At present, there remain very few long-term randomized controlled trials on public health interventions in China, but chronic diseases such as hypertension require long-term follow-up to better understand the effectiveness of interventions. (4) The risk probabilities of MI, stroke, and ESRD vary owing to different levels of hypertension. Therefore, in the Markov model structure used in this study, we classified hypertension into four states, namely, prehypertension, hypertension-L1, hypertension-L2, and hypertension-L3. We are not aware of any previous studies based on the Markov model that subdivide the hypertension state, owing to the lack of data on the transition probabilities among different levels of hypertension. However, the data collected by our community intervention trial can be used to calculate the transition probabilities among different levels of hypertension.

## Conclusions

From a healthcare system perspective, intervention through the integrated CCM indeed improved the QALYs of patients and allowed them to enjoy more continuous and coordinated services compared with the conventional management of hypertension in rural China. Additionally, the integrated CCM coupled with the payment method reform is favorable for motivating the personnel from all levels of medical institutions to improve efficiency through benefit-sharing mechanisms when compared with the general integrated CCM. Therefore, it is recommended that an integrated care delivery model that combines MDT, MIP, and SGB-P4P be adopted in the community management of hypertension in rural China, forming “patient-centered” continuous services to improve the efficiency of the healthcare system.

## Data Availability Statement

The raw data supporting the conclusions of this article will be made available by the authors, without undue reservation.

## Author Contributions

XK wrote this manuscript. WT and LZ designed the filed study, collected data, and revised this manuscript. All authors contributed to the article and approved the submitted version.

## Funding

This research was funded by National Natural Science Foundation of China (Grant No. 71603278), The Key Program of the National Natural Science Foundation of China (Grant No. 71734003), Research project on postgraduate education reform under double first-class capability construction from China Pharmaceutical University (Grant No. 3151920118), and National Natural Science Foundation of China (Grant No. 72174207).

## Author Disclaimer

The views expressed in the submitted article are the authors' own and not an official position of the institution or the funders.

## Conflict of Interest

The authors declare that the research was conducted in the absence of any commercial or financial relationships that could be construed as a potential conflict of interest.

## Publisher's Note

All claims expressed in this article are solely those of the authors and do not necessarily represent those of their affiliated organizations, or those of the publisher, the editors and the reviewers. Any product that may be evaluated in this article, or claim that may be made by its manufacturer, is not guaranteed or endorsed by the publisher.
