# Supplementary material for: Cost-Utility Analysis of the Integrated Care Models for the Management of Hypertension Patients: A Quasi-Experiment in Southwest Rural China
Source: Front Public Health. 2021 Dec 13;9:727829. doi: 10.3389/fpubh.2021.727829 (PMC8710505; doi:10.3389/fpubh.2021.727829)

Cost-Benefit Analysis of MDT+MIP+SGB_P4P, MDT+MIP, Placebo for hypertension treatment

Enter age of patient

**35**	Patient age

(Enter value between 35 and 80 years)

Enter WTP per QALY

72447	WTP

0.156

Deterministic Results (Dollar)

	Treatment	Cost	QALYs
	MDT+MIP+SGB_P4P	8992.24	15.808
	MDT+MIP	11229.54	15.331
	Control	10999.55	15.263
	MDT+MIP+SGB_P4P （2	-2237.3	0.477
**Incrementals:**	MDT+MIP（1 VS usual）	229.99	0.068
	Usual Practice（2 VS	-2007.31	0.545
	MDT+MIP+SGB_P4P （2	-4688.498833	
**ICER:**	MDT+MIP（1 VS usual）	3373.778629	per QALY
	Usual Practice（2 VS	-3680.71415	
	MDT+MIP+SGB_P4P （2	105950.0312	
**NMB:**	MDT+MIP（1 VS usual）	14586.11446	
	Usual Practice（2 VS	120536.1457	
	MDT+MIP+SGB_P4P （2	0.487482947	
**NHB:**	MDT+MIP（1 VS usual）	0.067111656	
	Usual Practice（2 VS	0.554594603	

Note:Due to the effect of rounding the decimal point, the results in the model are not completely consistent with the results in the manuscript, but the difference is not significant

Perspective
医疗卫生系统

Intervention

双干预　MDT+MIP+SGB -P4P

单干预　MDT+MIP

对照组　Usual Care

Markov Tree

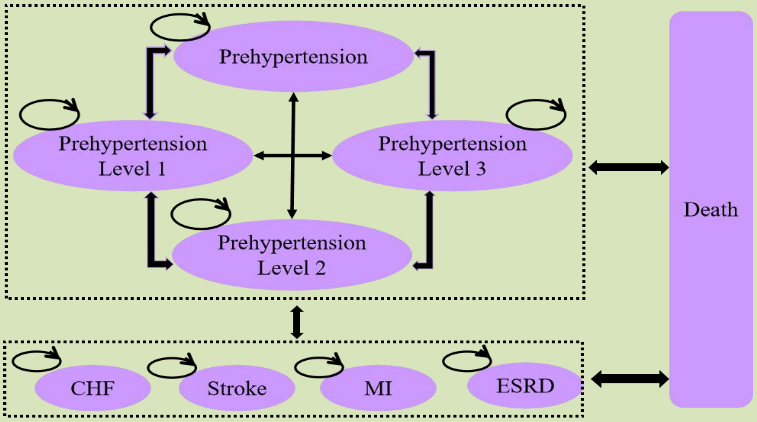

Normotensive 正常血压值：SBP为80~120，DBP为70~79；
Prehypertension 血压正常高值 :SBP为120~139 DBP为80~89
Hypertension L1:1级高血压（轻度）SBP为140~159 DBP为90~99；
L2:2级高血压（中度）SBP为160~179 DBP为100~109；
L3:3级高血压（重度）SBP为≥180 DBP为≥110；
MI：myocardial infarction 心肌梗塞；
stroke:中风；
CHF：congestive heart failure 缺血性心力衰竭；
ESRD：End stage renal disease 终末期肾病；

（1）模拟年龄从35岁开始，模拟到100岁，因此模型模拟时间为65年，即65个循环；
（2）对模型中的成本、效益等参数进行5%的贴现；
（3）所有高血压前期、正常血压都没有服用降压药物，因此没有治疗药物成本；
（4）高血压前期发展为高血压的风险比健康者发展为高血压的风险大　；
（5）各种并发症出现后，各种疾病的治疗费用为平均医药费用　；

Model Parameters

Name	Deterministic Model Parameters (Mean)	Probabilistic Model Parameters	Standard Error/Standard Deviation	Distribution	alpha	beta	Description	Source
Age	35.0000						Patient Age for Model	
Discount	0.0500							
**Interventions Efficacy**								
SSCR	0.6200	0.6464		Beta	270.0000	166.0000	单干预组血压控制率	
DSCR	0.6900	0.6822		Beta	346.0000	155.0000	双干预组血压控制率	
PSCR	0.6100	0.6324		Beta	190.0000	121.0000	空白组血压控制率	
P0.5	0.3234	0.3228		Beta	403.5570	844.4430		
P1	0.5303	0.2855		Beta	262.5707	549.4293		
P2	0.1205	0.3177		Beta	504.1229	1054.8771		
P3	0.0259	0.3479		Beta	666.1277	1393.8723		
**Transition Probability**								
p0.5_1	0.0413	0.0421		Beta	51.5424	1196.4576		
p0.5_2	0.0056	0.0054		Beta	6.9888	1241.0112		
p0.5_3	0.0016	0.0015		Beta	1.9968	1246.0032		
p1_0.5	0.1675	0.1493		Beta	209.0400	1038.9600		
p1_2	0.0072	0.0077		Beta	8.9856	1239.0144		
p1_3	0.0016	0.0012		Beta	1.9968	1246.0032		
p2_0.5	0.0227	0.0278		Beta	28.3296	1219.6704		
p2_1	0.0289	0.0245		Beta	36.0672	1211.9328		
p2_3	0.0008	0.0023		Beta	0.9984	1247.0016		
p3_0.5	0.0060	0.0051		Beta	7.4880	1240.5120		
p3_1	0.0060	0.0037		Beta	7.4880	1240.5120		
p3_2	0.0008	0.0014		Beta	0.9984	1247.0016		
p0.5_CVD	0.0014	0.0083		Beta	3575.0568	422026.9432		
p1_CVD	0.0022	0.0164		Beta	3100.6980	179293.3020		
p2_CVD	0.0035	0.0308		Beta	4086.9926	126072.0074		
p3_CVD	0.0035	0.0317		Beta	4086.9926	126072.0074		
p0.5_CHD	0.0002	0.0015		Beta	643.9005	428623.0995		
p1_CHD	0.0003	0.0026		Beta	521.3628	185679.6372		
p2_CHD	0.0004	0.0043		Beta	610.3080	135013.6920		
p3_CHD	0.0004	0.0045		Beta	610.3080	135013.6920		
p0.5_Stroke	0.0011	0.0046		Beta	2004.8931	424568.1069		
p1_Stroke	0.0020	0.0110		Beta	1959.5338	181174.4662		
p2_Stroke	0.0035	0.0219		Beta	2921.2331	128075.7669		
p3_Stroke	0.0035	0.0222		Beta	2921.2331	128075.7669		
p0.5_ESRD	0.0074	0.0019		Beta	1.2721	213.7279		
p1_ESRD	0.0152	0.0085		Beta	2.5758	212.4242		
p2_ESRD	0.0078	0.0100		Beta	1.3527	213.6473		
p3_ESRD	0.0017	0.0000		Beta	0.2909	214.7091		
pCVD_Death	0.0049	0.0401		Beta	49793.3750	1187035.6250		
pCHD_Death	0.0007	0.0060		Beta	7420.9740	1229408.0260		
pESRD_Death	0.0552	0.2457		Beta	126.7110	386.2890		
pStroke_Death	0.0040	0.0216		Beta	26810.2475	1210018.7525		
**Costs**								
cphuman	2210.1119	2447.3613	284.1425	Gamma	60.5001	36.5307		
cshuman	156257.8100	148761.1148	28251.9099	Gamma	30.5906	5108.0353		
cdhuman	76234.5296	116135.8689	30687.4274	Gamma	6.1714	12352.9089		
cpprogram	94.0000	51.9671	132.9361	Gamma	0.5000	188.0000		
csprogram	8163.9028	11204.0861	3497.8815	Gamma	5.4474	1498.6919		
cdproagram	4466.1765	4562.0435	164.5084	Gamma	737.0481	6.0595		
cprehy	898.4040	157.5409	1459.9820	Gamma	0.3787	2372.5934		
chy1	963.4320	769.0095	1902.5688	Gamma	0.2564	3757.1597		
chy2	1194.6120	82.0158	3192.0004	Gamma	0.1401	8529.0172		
chy3	2989.4760	1.3049	7526.1061	Gamma	0.1578	18947.2247		
cMI	31628.7790	69913.3662	16878.4042	Gamma	3.5116	9007.0037		
cCHF	11202.4460	1013.2466	12425.5121	Gamma	0.8128	13782.1106		
cStroke	12609.6855	12088.7774	4549.2906	Gamma	7.6828	1641.2816		
cESRD	123923.1180	181138.9720	22165.7258	Gamma	31.2565	3964.7114		
**Utilities**								
U_nor	0.6984	0.6293	0.1171	Beta	10.0370	4.3349		
U_pre	0.6514	0.6559	0.0229	Beta	282.5709	151.2359		
U_1	0.6553	0.6831	0.0253	Beta	230.7540	121.4078		
U_2	0.6830	0.6727	0.0630	Beta	36.5522	16.9649		
U_3	0.6690	0.7854	0.0784	Beta	23.4136	11.5843		
U_MI	0.6840	0.7554	0.0515	Beta	54.9517	25.3870		
U_Stroke	0.6050	0.6689	0.0515	Beta	53.8029	35.1275		
U_CHF	0.6400	0.6395	0.0160	Beta	575.3600	323.6400		
U_ESRD	0.6000	0.4829	0.2100	Beta	2.6653	1.7769		

Death Rates by age

Age	Deaths	Total Population	Rate
**35-39**	140531	121046434	**0.001160968**
**40-44**	216353	123217058	**0.001755869**
**45-49**	262531	100540459	**0.002611198**
**50-54**	337397	80681808	**0.004181822**
**55-59**	494339	79916406	**0.006185701**
**60-64**	586160	56869341	**0.010307135**
**65-69**	695662	40430322	**0.017206442**
**70-74**	999653	32626699	**0.030639109**
**75-79**	1162694	23477629	**0.049523485**
**80-84**	1081704	12754562	**0.084809184**
**85-89**	686462	5387168	**0.127425393**
**90-94**	279569	1465384	**0.190782075**
**95-99**	74729	344209	**0.217103562**
**100 and o**	16485	36283	**0.45434501**

Yearly probability of dying by age

Age	Index	Deaths
**35-39**	35	0.12%
**40-44**	40	0.18%
**45-49**	45	0.26%
**50-54**	50	0.42%
**55-59**	55	0.62%
**60-64**	60	1.03%
**65-69**	65	1.72%
**70-74**	70	3.06%
**75-79**	75	4.95%
**80-84**	80	8.48%
**85-89**	85	12.74%
**90-94**	90	19.08%
**95-99**	95	21.71%
**100 and o**	100	45.43%

MDT+MIP+
SGB-P4P

Deterministic Cohort Analysis

Cycle	Mortality Rate	Cohort Size	Normotensive	Prehypertension	Hypertension L1	Hypertension L2	Hypertension L3	MI	CHF	Stroke	ESRD	Dead	Cohort Check	Cost(RMB)	Cost(Dollar)	Lifeyears	QALYs
			Markov States														
		1000															
0	0.001		690	100.2425222	164.3977365	37.34033953	8.019401778	0	0	0	0	0	1000	397726.0559	62045.26472	1000	893.8855
1	0.001		689.1989323	122.6777494	137.2501687	36.52233436	8.287746944	0.6712525	0.0941568	0.5989648	3.537726527	1.160967699	1000	818853.9733	127741.2198	998.839	850.0333
2	0.001		688.3987946	139.2065845	116.3599198	35.69100676	8.541709115	1.3077568	0.1844267	1.164008	6.624201061	2.521592612	1000	1147428.275	178998.8109	997.4784	808.2558
3	0.001		687.5995858	151.2300971	100.2488085	34.85923886	8.78217083	1.9152057	0.2714387	1.7010469	9.33598095	4.056426726	1000	1402057.336	218720.9444	995.9436	768.4645
4	0.001		686.8013049	159.8182383	87.78835306	34.03623015	9.009881202	2.4979956	0.3556779	2.2146433	11.73334631	5.744329276	1000	1597200.261	249163.2408	994.2557	730.5735
5	0.002		685.5953719	165.6930647	78.0648123	33.20816614	9.22012579	3.0580457	0.437308	2.7070054	13.85706486	8.159035323	1000	1743322.775	271958.353	991.841	694.0856
6	0.002		684.3915563	169.5666985	70.48423897	32.40063275	9.418576036	3.5988213	0.5167347	3.1815966	15.7496666	10.69147818	1000	1850085.424	288613.3262	989.3085	659.3749
7	0.002		683.1898544	171.9193966	64.54069568	31.61641828	9.605754234	4.1224223	0.5941891	3.6405739	17.4421683	13.32852724	1000	1924708.258	300254.4882	986.6715	626.3606
8	0.002		681.9902626	173.1177198	59.84822167	30.8571646	9.782131464	4.6304988	0.6698522	4.0856241	18.9597187	16.05880606	1000	1973002.128	307788.332	983.9412	594.9646
9	0.002		680.7927771	173.4414727	56.11237615	30.12367385	9.948137934	5.1243564	0.7438673	4.5180743	20.322903	18.87236128	1000	1999684.361	311950.7603	981.1276	565.1118
10	0.003		679.0150927	172.9559063	53.06053652	29.3903741	10.09566197	5.6006536	0.8157119	4.935111	21.53136037	22.59959162	1000	2007029.365	313096.5809	977.4004	536.27
11	0.003		677.2420501	171.9731498	50.57450279	28.68402281	10.23332322	6.0637933	0.8859903	5.3407211	22.61461771	26.38782882	1000	1999790.329	311967.2914	973.6122	508.8767
12	0.003		675.4736374	170.6189943	48.5220783	28.0040082	10.36149481	6.5144909	0.9547807	5.7356093	23.58501433	30.2298918	1000	1980646.527	308980.8582	969.7701	482.8623
13	0.003		673.7098423	168.9893683	46.8025035	27.34951435	10.48052996	6.9533389	1.0221477	6.1203526	24.45309664	34.11930576	1000	1951832.716	304485.9036	965.8807	458.1599
14	0.003		671.9506528	167.1574285	45.33897241	26.71959472	10.59076485	7.3808352	1.0881456	6.4954294	25.2279763	38.05020014	1000	1915221.979	298774.6287	961.9498	434.7056
15	0.004		669.1406744	164.9164227	44.00172006	26.07125974	10.67588672	7.785812	1.1511114	6.8510401	25.87798338	43.52808946	1000	1869586.535	291655.4994	956.4719	411.7881
16	0.004		666.3424469	162.5780615	42.82139806	25.44738639	10.7525444	8.1789388	1.2125936	7.1965964	26.4476482	49.02238576	1000	1819186.933	283793.1615	950.9776	390.0671
17	0.004		663.555921	160.1752536	41.76322525	24.84679548	10.82107356	8.5605914	1.2726337	7.53244	26.94357922	54.52848673	1000	1765170.078	275366.5322	945.4715	369.4815
18	0.004		660.781048	157.732989	40.80071053	24.26832665	10.88179718	8.9311082	1.3312693	7.8588743	27.37169952	60.04217729	1000	1708503.454	266526.5389	939.9578	349.9734
19	0.004		658.0177789	155.2702257	39.91366666	23.71085236	10.93502646	9.2907982	1.3885345	8.1761727	27.73735476	65.55958983	1000	1650003.041	257400.4744	934.4404	331.4877
20	0.006		653.9474776	152.4901821	39.00671783	23.12577344	10.95914919	9.6213286	1.4416787	8.4682003	27.9898165	72.94967575	1000	1587268.224	247613.8429	927.0503	313.3392
21	0.006		649.902354	149.7253766	38.15157239	22.56181554	10.97622711	9.9402211	1.4932953	8.7503679	28.18797411	80.31079598	1000	1524192.562	237774.0397	919.6892	296.1788
22	0.006		645.8822523	146.9839353	37.33895325	22.01787183	10.98658226	10.247792	1.5434206	9.0229455	28.33621241	87.64003504	1000	1461263.139	227957.0496	912.36	279.9533
23	0.006		641.8870177	144.2718801	36.56176469	21.4928962	10.99052491	10.54434	1.5920896	9.286188	28.43855822	94.9347402	1000	1398882.921	218225.7357	905.0653	264.6127
24	0.006		637.9164965	141.593632	35.81456607	20.98590265	10.98835405	10.830155	1.6393354	9.5403379	28.49872043	102.1924999	1000	1337382.872	208631.7281	897.8075	250.1092
25	0.010		631.3414048	138.3688245	34.94556534	20.40947179	10.93507007	11.060876	1.6784339	9.746307	28.40266756	113.1113793	1000	1271881.017	198413.4387	886.8886	235.4162
26	0.010		624.8340834	135.2074776	34.10569323	19.85361974	10.87649109	11.279318	1.7158222	9.9417779	28.27133024	123.9143865	1000	1208229.63	188483.8222	876.0856	221.5839
27	0.010		618.3938339	132.1105286	33.29212382	19.31730311	10.81295226	11.485879	1.7515494	10.127083	28.10808201	134.6006652	1000	1146581.573	178866.7254	865.3993	208.5624
28	0.010		612.0199649	129.0783552	32.5026248	18.79954499	10.74477407	11.680939	1.7856632	10.302544	27.91603189	145.1695577	1000	1087048.345	169579.5418	854.8304	196.3046
29	0.010		605.7117922	126.1109149	31.73541372	18.29943095	10.67226297	11.864871	1.8182097	10.46847	27.69804708	155.6205875	1000	1029706.509	160634.2154	844.3794	184.7659
30	0.017		595.2896472	122.3377728	30.77009718	17.68985162	10.52208064	11.956172	1.8366891	10.552936	27.26567562	171.7790772	1000	967966.088	151002.7097	828.2209	172.6905
31	0.017		585.0468303	118.6744764	29.83625549	17.10379929	10.36970252	12.035225	1.853348	10.626794	26.81710395	187.6364643	1000	909242.2187	141841.7861	812.3635	161.4035
32	0.017		574.9802558	115.1184846	28.93238102	16.54011753	10.21545941	12.102603	1.8682631	10.69053	26.3549734	203.1969324	1000	853485.8782	133143.797	796.8031	150.8537
33	0.017		565.0868913	111.6671721	28.05715119	15.99772322	10.05966178	12.158856	1.8815083	10.744609	25.88168397	218.4647434	1000	800631.5627	124898.5238	781.5353	140.9931
34	0.017		555.3637563	108.3178686	27.20938756	15.47560117	9.90260078	12.204513	1.893155	10.78948	25.39941447	233.444223	1000	750600.8845	117093.738	766.5558	131.7769
35	0.031		538.3479054	103.6128897	26.02253044	14.76492054	9.61153085	12.076144	1.8778417	10.680642	24.56895906	258.4366361	1000	693899.6856	108248.351	741.5634	121.4771
36	0.031		521.8534051	99.11140868	24.88807541	14.08896631	9.325758587	11.939422	1.8611346	10.564419	23.75101709	282.616393	1000	641150.0908	100019.4142	717.3836	111.9824
37	0.031		505.8642815	94.80484977	23.80358532	13.44584635	9.045442865	11.795194	1.8431575	10.441538	22.9470915	306.0090126	1000	592127.0758	92371.82382	693.991	103.2299
38	0.031		490.3650504	90.68495275	22.76676748	12.83378906	8.77071508	11.644252	1.8240268	10.312683	22.15844144	328.6393217	1000	546610.3201	85271.20994	671.3607	95.1616
39	0.031		475.340702	86.74377164	21.77545727	12.25113434	8.501681418	11.487337	1.8038519	10.178493	21.3861086	350.5314627	1000	504385.7	78684.1692	649.4685	87.72412
40	0.050		451.800174	81.33556851	20.41638929	11.46497022	8.077876041	11.10821	1.748671	9.8473531	20.2270777	383.9737096	1000	456391.4426	71197.06505	616.0263	79.29043
41	0.050		429.425455	76.26443965	19.14239935	10.73051378	7.672884621	10.734675	1.6940863	9.5209142	19.12203619	415.6925963	1000	412830.3062	64401.52777	584.3074	71.66779
42	0.050		408.1588101	71.50945462	17.94812614	10.04422147	7.286080169	10.367346	1.6402013	9.1997198	18.06954419	445.7764962	1000	373317.6964	58237.56063	554.2235	64.77823
43	0.050		387.9453635	67.05096521	16.82855417	9.402808615	6.916835582	10.006751	1.5871067	8.8842353	17.06804803	474.3093319	1000	337498.0978	52649.70325	525.6907	58.55126
44	0.050		368.7329573	62.8705305	15.77898925	8.803228789	6.564526453	9.6533344	1.5348803	8.5748557	16.11590894	501.3707885	1000	305043.6406	47586.80794	498.6292	52.92314
45	0.085		337.4610159	56.73241483	14.23826372	7.932026867	5.996899617	8.9668422	1.4294298	7.9693417	14.64276648	544.6309988	1000	265718.3859	41452.0682	455.369	46.05778
46	0.085		308.8412224	51.19370856	12.84811931	7.147727318	5.476861828	8.3246306	1.330502	7.4025916	13.29943901	584.1351974	1000	231458.4117	36107.51223	415.8648	40.08328
47	0.085		282.6486502	46.19589229	11.59382225	6.441559984	5.000604307	7.7243956	1.2377803	6.8726075	12.07521861	620.209469	1000	201621.527	31452.95821	379.7905	34.88402
48	0.085		258.6774487	41.68615282	10.46208313	5.805654963	4.564600493	7.1638791	1.150952	6.3774296	10.96016622	653.1516329	1000	175644.5249	27400.54589	346.8484	30.35939
49	0.085		236.7392253	37.61682851	9.440914739	5.232947937	4.16558809	6.6408801	1.0697099	5.9151467	9.945075535	683.2336832	1000	153033.9701	23873.29934	316.7663	26.42183
50	0.127		206.5726365	32.34182156	8.117167957	4.494087387	3.623030264	5.8702546	0.9481672	5.2318224	8.597613506	724.2033987	1000	127446.695	19881.68443	275.7966	21.92278
51	0.127		180.2500371	27.80668923	6.979104555	3.859855636	3.15034211	5.1865709	0.8400359	4.6252435	7.430531284	759.8715898	1000	106224.768	16571.0638	240.1284	18.18998
52	0.127		157.2816052	23.90764401	6.000667342	3.315378245	2.738654766	4.5804142	0.7438989	4.0871368	6.420099621	790.9245009	1000	88623.59079	13825.28016	209.0755	15.09291
53	0.127		137.2399349	20.5554568	5.1594575	2.847906501	2.380204082	4.0433246	0.6584784	3.6100649	5.545630388	817.9595419	1000	74024.53361	11547.82724	182.0405	12.52329
54	0.127		119.7520822	17.67341547	4.436220645	2.446511492	2.068196506	3.5677117	0.5826245	3.1873508	4.789102598	841.496784	1000	61913.91381	9658.570554	158.5032	10.39125
55	0.191		96.9055315	14.07584036	3.533340949	1.946819769	1.665656591	2.9207359	0.4783902	2.6110687	3.831409611	872.0312064	1000	48478.8938	7562.707433	127.9688	7.995275
56	0.191		78.41769314	11.21068032	2.814253844	1.549295464	1.34113004	2.3900082	0.3926298	2.1380267	3.064460213	896.6818222	1000	38136.06342	5949.225893	103.3182	6.15183
57	0.191		63.45700293	8.928808872	2.241538852	1.233022432	1.079572317	1.9548761	0.3221069	1.7499371	2.45045842	916.582676	1000	30166.22283	4705.930762	83.41732	4.733482
58	0.191		51.35054425	7.111465756	1.785395859	0.981373372	0.868823409	1.5983045	0.2641437	1.431706	1.959048306	932.6491948	1000	24017.34971	3746.706555	67.35081	3.642191
59	0.191		41.55378087	5.664073228	1.422092981	0.781128669	0.699058549	1.3062534	0.2165266	1.1708856	1.565861969	945.620338	1000	19265.87836	3005.477024	54.37966	2.802531
60	0.217		32.53230703	4.362224757	1.095299032	0.601216256	0.543942409	1.0327781	0.1717278	0.926399	1.210131861	957.5239737	1000	15217.66061	2373.955055	42.47603	2.086218
61	0.217		25.46942729	3.359632859	0.843612333	0.462767267	0.423150138	0.8162378	0.1361456	0.7326782	0.935035163	966.8213134	1000	12175.64452	1899.400545	33.17869	1.553013
62	0.217		19.9399239	2.587499519	0.649768423	0.356218894	0.329110291	0.6448565	0.1078964	0.5792508	0.72234501	974.0831303	1000	9880.879878	1541.417261	25.91687	1.156104
63	0.217		15.61089539	1.992844768	0.50047191	0.274215745	0.255915691	0.5092752	0.0854785	0.457788	0.557940629	979.7551742	1000	8141.400689	1270.058507	20.24483	0.860647
64	0.217		12.2217144	1.534869474	0.385483852	0.211099766	0.198959087	0.40206	0.0676954	0.36167	0.430886242	984.1855617	1000	6814.912726	1063.126385	15.81444	0.640707
65	0.454		6.668839446	0.818019877	0.205466265	0.112436562	0.107446854	0.2219247	0.0375343	0.1998352	0.230491425	991.3980053	1000	5059.243338	789.2419607	8.601995	0.332216
														Dcosted	Dcosted$	Dlifed	Dqalyd
													Per patient	57421.72175	8957.788594	42.18587	15.80799

Probabilitics Analysis

Cycle	Mortality Rate	Cohort Size	Normotensive	Prehypertension	Hypertension L1	Hypertension L2	Hypertension L3	MI	CHF	Stroke	ESRD	Dead	Cohort Check	Cost（￥）	Cost（$）	Lifeyears	QALYs
			Markov States														
0	0.001	1000															
1	0.001		682.2192337	102.758534	168.5239957	38.2775539	8.220682717	0	0	0	0	0	1000	269633.23	42062.78387	1000	896.9422
2	0.001		681.4271992	122.1972337	140.4620258	35.44921531	8.088795482	5.0649675	0.7952897	3.3435194	2.010786194	1.160967699	1000	1010449.114	157630.0618	998.839	895.2682
3	0.001		680.6360843	136.074715	118.7024944	32.85709786	7.954135881	9.531221	1.5269515	6.3285169	3.294329508	3.094453611	1000	1577266.187	246053.5252	996.9055	893.3252
4	0.001		679.8458878	145.7081758	101.7697199	30.49056986	7.8169237	13.487717	2.2048395	9.0115991	4.075787994	5.5887793	1000	2016854.707	314629.3343	994.4112	891.1821
5	0.002		678.6521675	152.0199548	88.47472923	28.31794867	7.672831412	16.998161	2.8354367	11.432417	4.51210146	9.08425265	1000	2361810.693	368442.4681	990.9157	888.3574
6	0.002		677.4605433	155.8622674	78.03089763	26.34485107	7.527058903	20.124649	3.4255015	13.629448	4.717338993	12.87744369	1000	2637139.759	411393.8024	987.1226	885.4182
7	0.002		676.2710114	157.8319598	69.77316853	24.55522934	7.380016615	22.915546	3.9800634	15.632068	4.770334085	16.89060243	1000	2860444.444	446229.3332	983.1094	882.3887
8	0.002		675.0835681	158.3861099	63.19323969	22.93313213	7.23211832	25.410771	4.5031739	17.464097	4.725704519	21.06808577	1000	3044380.904	474923.421	978.9319	879.2861
9	0.002		673.8982099	157.8748079	57.90256429	21.46314133	7.083766169	27.643561	4.9981222	19.145002	4.620919337	25.36990501	1000	3198053.843	498896.3995	974.6301	876.123
10	0.003		672.1385285	156.4311915	53.55456158	20.11229004	6.929282067	29.618201	5.4633255	20.674456	4.477415179	30.60074798	1000	3325391.88	518761.1334	969.3993	872.1592
11	0.003		670.3834421	154.3980812	49.97899883	18.88759203	6.775337742	31.378618	5.9044826	22.07955	4.316318968	35.8975792	1000	3433549.994	535633.7991	964.1024	868.1574
12	0.003		668.6329385	151.9343543	47.00015676	17.7761349	6.622255546	32.945622	6.3235082	23.371803	4.148732253	41.24449454	1000	3526024.149	550059.7673	958.7555	864.1233
13	0.003		666.8870058	149.1615038	44.48372127	16.76611886	6.470325221	34.33719	6.7220181	24.560992	3.981671898	46.62945276	1000	3605376.82	562438.7839	953.3705	860.0617
14	0.003		665.1456321	146.172391	42.32698208	15.84683454	6.319804147	35.568985	7.1013903	25.655494	3.819378172	52.04310878	1000	3673508.536	573067.3315	947.9569	855.9767
15	0.004		662.3641111	142.8083562	40.38487348	14.98372613	6.160992433	36.5989	7.451659	26.622264	3.65823188	58.96688495	1000	3726271.041	581298.2824	941.0331	850.5275
16	0.004		659.5942219	139.3632927	38.66970231	14.19566905	6.004496009	37.491664	7.7838947	27.504833	3.505884424	65.88634224	1000	3770170.314	588146.569	934.1137	845.0769
17	0.004		656.835916	135.8788122	37.13430528	13.4745388	5.850474205	38.258779	8.0990907	28.309176	3.362666574	72.796241	1000	3806113.456	593753.6991	927.2038	839.6281
18	0.004		654.0891448	132.3863685	35.74254898	12.81309528	5.699059971	38.910569	8.3981331	29.040618	3.228421687	79.69204051	1000	3834835.623	598234.3572	920.308	834.184
19	0.004		651.3538601	128.9096338	34.46670754	12.20490128	5.550362303	39.45635	8.68182	29.70394	3.102711253	86.56971428	1000	3856949.197	601684.0747	913.4303	828.7475
20	0.006		647.3247798	125.2079984	33.21639819	11.61978693	5.393346357	39.825507	8.9334785	30.243942	2.978732915	95.25602947	1000	3865491.044	603016.6029	904.744	821.6605
21	0.006		643.3206222	121.5672371	32.04913373	11.07944297	5.23980946	40.103171	9.1701257	30.721804	2.862544497	103.8861094	1000	3868358.033	603463.8532	896.1139	814.6115
22	0.006		639.3412331	117.9966594	30.95173276	10.57912621	5.089783418	40.296934	9.3924599	31.1415	2.75345178	112.4571196	1000	3865994.932	603095.2094	887.5429	807.6026
23	0.006		635.3864594	114.5028097	29.91396466	10.11464839	4.943284467	40.413753	9.6011296	31.506692	2.650793316	120.9664664	1000	3858806.069	601973.7467	879.0335	800.6361
24	0.006		631.4561486	111.0901206	28.92784787	9.682312992	4.800315228	40.460021	9.7967406	31.820764	2.553957661	129.4117716	1000	3847163.488	600157.5041	870.5882	793.714
25	0.010		624.9476446	107.3035629	27.86789233	9.23895322	4.641082303	40.274876	9.9394839	31.955713	2.451864907	141.3789262	1000	3816054.202	595304.4555	858.6211	783.5668
26	0.010		618.5062246	103.6319056	26.85661253	8.825086101	4.486581421	40.031348	10.068928	32.043964	2.355344191	153.194006	1000	3781352.375	589890.9705	846.806	773.5377
27	0.010		612.1311971	100.0745727	25.88950526	8.437828888	4.336733595	39.735193	10.185734	32.088821	2.263893436	164.8565208	1000	3743406.407	583971.3996	835.1435	763.6273
28	0.010		605.821878	96.63027898	24.96290456	8.074641004	4.191453181	39.391708	10.290528	32.093369	2.177070938	176.3661682	1000	3702541.307	577596.4439	823.6338	753.8361
29	0.010		599.5775898	93.29721789	24.07378689	7.733281515	4.050649154	39.005772	10.383901	32.06049	2.094489118	187.7228227	1000	3659060.573	570813.4494	812.2772	744.1643
30	0.017		589.2609927	89.42951916	23.05353103	7.35841745	3.886279571	38.312769	10.394774	31.771684	2.001357625	204.5306758	1000	3588835.453	559858.3306	795.4693	729.4778
31	0.017		579.1219074	85.7173448	22.07901566	7.006458313	3.728267821	37.593662	10.394679	31.453134	1.913014183	220.9925172	1000	3517343.532	548705.591	779.0075	715.0783
32	0.017		569.1572798	82.15536129	21.14758382	6.675432399	3.576403497	36.852902	10.384346	31.107858	1.829110142	237.1137234	1000	3444867.004	537399.2526	762.8863	700.9614
33	0.017		559.3641079	78.73822261	20.25687345	6.363591836	3.430478719	36.094529	10.364464	30.738656	1.749336216	252.8997405	1000	3371664.053	525979.5923	747.1003	687.1229
34	0.017		549.7394417	75.4606215	19.40475931	6.069383744	3.290288767	35.32221	10.335683	30.348125	1.673416857	268.3560711	1000	3297970.468	514483.393	731.6439	673.5586
35	0.031		532.8959148	71.30368968	18.32865102	5.709897199	3.111435247	34.064793	10.159782	29.531018	1.578626962	293.316192	1000	3181321.892	496286.2152	706.6838	651.2163
36	0.031		516.5684586	67.37399876	17.31284099	5.373988178	2.942129839	32.827165	9.9790392	28.713705	1.489467724	317.4192068	1000	3067153.478	478475.9426	682.5808	629.6182
37	0.031		500.7412611	63.65945422	16.35379364	5.059781323	2.7818841	31.611843	9.7942677	27.898637	1.405558906	340.6935191	1000	2955597.966	461073.2828	659.3065	608.7402
38	0.031		485.3989948	60.1485394	15.44822062	4.765593033	2.630231807	30.420932	9.6062147	27.087993	1.326553814	363.1667272	1000	2846762.407	444094.9355	636.8333	588.5592
39	0.031		470.5268019	56.83030241	14.59305371	4.489907356	2.486728336	29.256173	9.4155651	26.283698	1.252135126	384.8656346	1000	2740730.791	427554.0034	615.1344	569.0525
40	0.050		447.224675	52.62113481	13.50984199	4.146566181	2.303989682	27.566497	9.0451387	24.991102	1.158365595	417.432689	1000	2588088.781	403741.8498	582.5673	539.4518
41	0.050		425.0765507	48.72307525	12.50718305	3.830485068	2.134587689	25.958898	8.6839486	23.747492	1.071717471	448.2660624	1000	2443189.719	381137.5961	551.7339	511.3972
42	0.050		404.0252787	45.11325378	11.57904786	3.53933057	1.9775616	24.431203	8.3323283	22.552602	0.991631014	477.4577628	1000	2305769.113	359699.9816	522.5422	484.8081
43	0.050		384.016539	41.77045656	10.71986903	3.27099635	1.832018078	22.981045	7.9905286	21.40594	0.917594131	505.0950135	1000	2175555.106	339386.5965	494.905	459.6083
44	0.050		364.9987018	38.67501126	9.924502671	3.023576976	1.697126746	21.605906	7.6587277	20.306821	0.849137621	531.2604884	1000	2052271.374	320154.3343	468.7395	435.7251
45	0.085		334.0434596	34.44400434	8.838001067	2.688656253	1.512231664	19.540779	7.0667959	18.537864	0.755868604	572.5723389	1000	1867482.683	291327.2985	427.4277	397.7151
46	0.085		305.7135062	30.67563892	7.870479966	2.391225909	1.347435531	17.664184	6.517272	16.914191	0.672878993	610.2331878	1000	1699703.597	265153.7611	389.7668	363.0278
47	0.085		279.7861931	27.3193771	7.008900559	2.12700855	1.200560999	15.960186	6.007549	15.425055	0.599028202	644.5661419	1000	1547462.618	241404.1684	355.4339	331.3725
48	0.085		256.0577543	24.33019047	6.241655696	1.8922313	1.069665309	14.414004	5.5351202	14.060336	0.533303918	675.8657392	1000	1409401.752	219866.6733	324.1343	302.4838
49	0.085		234.341705	21.66796199	5.558411864	1.683562997	0.953015237	13.011958	5.0975847	12.810529	0.474807287	704.4004641	1000	1284269.901	200346.1046	295.5995	276.1196
50	0.127		204.4806211	18.37354591	4.713090418	1.426312759	0.808450791	11.186893	4.4754119	11.120794	0.402505413	743.012374	1000	1121329.242	174927.3618	256.9876	240.2919
51	0.127		178.4245976	15.57994972	3.996332137	1.208492527	0.685797613	9.6136097	3.9274335	9.6494995	0.341223505	776.5730643	1000	980884.4177	153017.9692	223.4269	209.1184
52	0.127		155.6887731	13.21105544	3.38858203	1.024028142	0.581738388	8.2581378	3.445092	8.3691713	0.289279331	805.7441425	1000	859887.5402	134142.4563	194.2559	181.9939
53	0.127		135.85007	11.20230959	2.873260153	0.867788784	0.493457256	7.0909499	3.0207675	7.2556566	0.245248136	831.100492	1000	755693.0879	117888.1217	168.8995	158.392
54	0.127		118.5393214	9.498967334	2.436308784	0.735438579	0.418564175	6.0864051	2.6476837	6.2877461	0.207923059	853.1416417	1000	666006.7566	103897.054	146.8584	137.8548
55	0.191		95.92414374	7.452779941	1.911452146	0.576717058	0.328511819	4.8366455	2.1520728	5.0484647	0.163108358	881.6061039	1000	554291.4788	86469.47069	118.3939	111.2498
56	0.191		77.62353657	5.847350116	1.499667195	0.452279336	0.257828192	3.8419368	1.7485156	4.0516745	0.12795508	904.5492566	1000	465341.6553	72593.29822	95.45074	89.78246
57	0.191		62.8143572	4.587741546	1.17659408	0.354711096	0.202348928	3.0506092	1.4200768	3.2503371	0.10037963	923.0428444	1000	394547.3486	61549.38637	76.95716	72.46003
58	0.191		50.8305038	3.599465053	0.923121117	0.278204301	0.158804609	2.4213673	1.1528999	2.6064426	0.078747995	937.9504433	1000	338225.0343	52763.10535	62.04956	58.48179
59	0.191		41.13295482	2.824075239	0.724254053	0.218208309	0.124628547	1.9212302	0.9356554	2.0892994	0.061778664	949.9679153	1000	293433.1578	45775.57262	50.03208	47.20176
60	0.217		32.20284381	2.141381377	0.549165528	0.165413478	0.094525412	1.4733051	0.7344591	1.619149	0.046840427	960.9729167	1000	253277.3388	39511.26485	39.02708	36.85636
61	0.217		25.21149171	1.623720244	0.416404849	0.125396528	0.071692274	1.1294143	0.5763248	1.2543175	0.035514632	969.5557231	1000	222425.6765	34698.40553	30.44428	28.77952
62	0.217		19.73798705	1.231197981	0.315739154	0.095063363	0.054373757	0.8655009	0.4520864	0.9713351	0.026927583	976.2497886	1000	198730.5969	31001.97311	23.75021	22.47353
63	0.217		15.45279976	0.933564134	0.23940942	0.07206961	0.041238227	0.6630426	0.3545165	0.7519303	0.020416934	981.4710125	1000	180538.0359	28163.9336	18.52899	17.54996
64	0.217		12.09794189	0.707880675	0.181532398	0.054638782	0.031275531	0.507786	0.2779188	0.5818869	0.01548055	985.5436584	1000	166574.618	25985.6404	14.45634	13.70559
65	0.454		6.601302358	0.368815797	0.094580119	0.028462024	0.016299557	0.2683003	0.151873	0.3121026	0.008065115	992.1501991	1000	144976.6127	22616.35158	7.849801	7.452229
														Dcostp ￥	Dcostp$	Dlifep	Dqalyp
													Per patient	152524.0566	23793.75283	40.00591	36.45263

MDT+MIP

Deterministic Cohort Analysis

Markov States

Cycle	Mortality Rate	Cohort Size	Normotensive	Prehypertension	Hypertension L1	Hypertension L2	Hypertension L3	MI	CHF	Stroke	ESRD	Dead	Cohort Check	Cost(¥)	Cost($)	Lifeyears	QALYs
		1000															
0	0.00116097		620	122.8779305	201.519806	45.7720291	9.830234438	0	0	0	0	0	1000	553033.4319	86273.21537	1000	869.9242
1	0.00116097		619.2802	150.3791767	168.2421423	44.76931308	10.15917367	0.822826	0.115418	0.734215	4.336568	1.160968	1000	1066135.785	166317.1824	998.839	827.1722
2	0.00116097		618.5612357	170.6403294	142.6347404	43.75026635	10.47048214	1.603057	0.226071	1.426849	8.119988	2.566981	1000	1465933.841	228685.6792	997.433	786.4273
3	0.00116097		617.8431061	185.3788287	122.8856362	42.73067989	10.76524166	2.347671	0.332731	2.085154	11.44411	4.186845	1000	1775230.793	276936.0038	995.8132	747.6072
4	0.00116097		617.1258102	195.9062276	107.6115296	41.7218305	11.04437051	3.062059	0.435992	2.714724	14.38281	5.994645	1000	2011743.971	313832.0595	994.0054	710.6314
5	0.00175587		616.0422182	203.1076277	95.69235056	40.70678429	11.30208968	3.748572	0.536055	3.318265	16.98608	8.559958	1000	2188295.911	341374.1621	991.44	675.0188
6	0.00175587		614.9605288	207.8559531	86.40003487	39.71690466	11.54535127	4.411458	0.633417	3.900022	19.30604	11.27029	1000	2316722.464	361408.7044	988.7297	641.1354
7	0.00175587		613.8807388	210.7399055	79.11440116	38.7556095	11.77479551	5.053292	0.728361	4.462639	21.38072	14.10954	1000	2405868.195	375315.4384	985.8905	608.9043
8	0.00175587		612.8028447	212.2088178	73.36233624	37.82491145	11.99099986	5.676095	0.821109	5.008184	23.24095	17.06376	1000	2462850.542	384204.6846	982.9362	578.2509
9	0.00175587		611.7268432	212.6056762	68.7829127	36.92579375	12.19449166	6.281469	0.911837	5.538285	24.91195	20.12075	1000	2493446.757	388977.6941	979.8793	549.1032
10	0.0026112		610.1295036	212.0104658	65.04194799	36.02691018	12.37532758	6.865317	0.999905	6.049491	26.39328	24.10785	1000	2500439.798	390068.6084	975.8921	520.9445
11	0.0026112		608.5363349	210.8057965	61.99455181	35.16106022	12.54407363	7.433037	1.086053	6.54669	27.72114	28.17126	1000	2489651.367	388385.6133	971.8287	494.2011
12	0.0026112		606.9473263	209.145864	59.47867663	34.32749392	12.70118719	7.985505	1.170376	7.030747	28.91066	32.30216	1000	2464361.177	384440.3436	967.6978	468.8053
13	0.0026112		605.362467	207.1482579	57.37081074	33.52521114	12.84710124	8.523448	1.252955	7.502368	29.97476	36.49262	1000	2427304.264	378659.4653	963.5074	444.6925
14	0.0026112		603.781746	204.9026543	55.57680489	32.7530516	12.98222788	9.047475	1.333856	7.962139	30.92462	40.73543	1000	2380772.533	371400.5152	959.2646	421.8006
15	0.00418182		601.2568379	202.155615	53.93759233	31.95831839	13.08657082	9.543899	1.41104	8.398049	31.7214	46.53068	1000	2323257.022	362428.0955	953.4693	399.4394
16	0.00418182		598.7424885	199.2892366	52.49074601	31.19357042	13.1805383	10.0258	1.486405	8.821634	32.4197	52.34989	1000	2259976.59	352556.348	947.6501	378.2489
17	0.00418182		596.2386537	196.3438592	51.19363095	30.45736221	13.26454179	10.49363	1.560003	9.233314	33.02761	58.18739	1000	2192333.547	342004.0333	941.8126	358.1699
18	0.00418182		593.7452895	193.3501156	50.0137742	29.74827138	13.33897718	10.94781	1.631878	9.633459	33.55241	64.03802	1000	2121510.44	330955.6286	935.962	339.1457
19	0.00418182		591.2623521	190.3312444	48.92643009	29.06491579	13.40422599	11.38872	1.702075	10.02241	34.00063	69.897	1000	2048504.261	319566.6647	930.103	321.1224
20	0.0061857		587.6049799	186.923449	47.81468637	28.34772228	13.43379578	11.79389	1.767219	10.38037	34.3101	77.62379	1000	1970369.237	307377.6009	922.3762	303.4359
21	0.0061857		583.9702311	183.5343326	46.76644357	27.65641905	13.45473	12.18479	1.830491	10.72626	34.553	85.32331	1000	1891875.179	295132.5279	914.6767	286.7164
22	0.0061857		580.3579658	180.1738561	45.77032979	26.98964934	13.46742342	12.56181	1.891935	11.06038	34.73471	92.99193	1000	1813616.357	282924.1516	907.0081	270.9122
23	0.0061857		576.7680449	176.8494014	44.81764704	26.34613083	13.47225634	12.92532	1.951594	11.38307	34.86017	100.6264	1000	1736084.066	270829.1142	899.3736	255.974
24	0.0061857		573.2003302	173.5663876	43.90172615	25.72465486	13.46959528	13.27567	2.009508	11.69461	34.93392	108.2236	1000	1659681.464	258910.3084	891.7764	241.8552
25	0.01030714		567.2922768	169.6133978	42.83649945	25.01806219	13.40427944	13.55849	2.057435	11.94709	34.81617	119.4563	1000	1578421.776	246233.7971	880.5437	227.5621
26	0.01030714		561.4451184	165.7381984	41.8069788	24.33669517	13.33247295	13.82626	2.103266	12.1867	34.65518	130.5691	1000	1499476.456	233918.3271	869.4309	214.1107
27	0.01030714		555.6582275	161.9419383	40.80970017	23.67927478	13.25458664	14.07946	2.147061	12.41384	34.45507	141.5608	1000	1423030.69	221992.7876	858.4392	201.4519
28	0.01030714		549.9309829	158.2250806	39.84192718	23.04460353	13.17101338	14.31857	2.188878	12.62892	34.21965	152.4304	1000	1349219.066	210478.1742	847.5696	189.5395
29	0.01030714		544.2627698	154.5875731	38.90147488	22.43156051	13.0821288	14.54404	2.228773	12.83232	33.95244	163.1769	1000	1278133.445	199388.8174	836.8231	178.3298
30	0.01720644		534.8979439	149.9624311	37.71818364	21.68433424	12.89803433	14.65595	2.251425	12.93586	33.42244	179.5734	1000	1201693.897	187464.248	820.4266	166.61
31	0.01720644		525.6942533	145.4719389	36.57347448	20.96594751	12.71124825	14.75286	2.271846	13.02639	32.87258	195.6595	1000	1128988.141	176122.1501	804.3405	155.6595
32	0.01720644		516.6489255	141.1129812	35.46549932	20.27498277	12.52217605	14.83545	2.290129	13.10452	32.3061	211.4392	1000	1059954.366	165352.8811	788.5608	145.4279
33	0.01720644		507.7592356	136.88234	34.39263695	19.61011233	12.33119831	14.9044	2.306365	13.17081	31.72594	226.917	1000	994510.6399	155143.6598	773.083	135.8683
34	0.01720644		499.0225057	132.7767421	33.35344282	18.97009176	12.13867192	14.96037	2.320642	13.22581	31.13477	242.097	1000	932559.3162	145479.2533	757.903	126.9367
35	0.03063911		483.7329005	127.0093486	31.8985857	18.09893486	11.78187653	14.80301	2.30187	13.0924	30.11679	267.1643	1000	862460.9109	134543.9021	732.8357	116.9682
36	0.03063911		468.9117553	121.4914042	30.50796341	17.2703458	11.43157504	14.63542	2.281391	12.94993	29.11415	291.4061	1000	797234.6791	124368.6099	708.5939	107.7824
37	0.03063911		454.5447167	116.2123965	29.17858846	16.4820052	11.08796222	14.45863	2.259354	12.7993	28.12869	314.8484	1000	736603.4386	114910.1364	685.1516	99.31803
38	0.03063911		440.6178714	111.1622001	27.90765046	15.73174143	10.75119913	14.2736	2.235904	12.64135	27.16196	337.5165	1000	680295.8368	106126.1505	662.4835	91.5185
39	0.03063911		427.1177322	106.3310749	26.69249601	15.01751952	10.42141593	14.08125	2.211173	12.47686	26.21523	359.4352	1000	628048.1784	97975.51584	640.5648	84.33164
40	0.04952348		405.9653738	99.70166462	25.02654171	14.05383446	9.901912566	13.61652	2.143532	12.07095	24.79448	392.7252	1000	568751.3208	88725.20604	607.2748	76.19267
41	0.04952348		385.8605538	93.48544215	23.46487662	13.15353302	9.40547147	13.15863	2.076622	11.6708	23.43992	424.2842	1000	514910.7632	80326.07906	575.7158	68.83946
42	0.04952348		366.7513946	87.65675082	22.00092882	12.31227148	8.931324078	12.70836	2.010569	11.27708	22.14976	454.2016	1000	466054.0126	72704.42596	545.7984	62.19617
43	0.04952348		348.5885875	82.19150574	20.62855027	11.52602346	8.478701681	12.26634	1.945486	10.89035	20.92212	482.5623	1000	421744.2712	65792.10631	517.4377	56.19428
44	0.04952348		331.3252659	77.0671019	19.34198682	10.79105464	8.046838878	11.83312	1.881466	10.51111	19.75499	509.4471	1000	381578.6776	59526.2737	490.5529	50.77187
45	0.08480918		303.2258404	69.54296011	17.45335552	9.723129708	7.351038241	10.99161	1.752204	9.76887	17.9492	552.2418	1000	333009.0402	51949.41026	447.7582	44.16677
46	0.08480918		277.5095042	62.75357823	15.74930754	8.761730261	6.713572563	10.20439	1.630938	9.074145	16.30254	591.3003	1000	290665.8129	45343.86681	408.6997	38.42125
47	0.08480918		253.9741495	56.6272228	14.21178211	7.896105787	6.129773021	9.468614	1.517279	8.424487	14.80188	626.9487	1000	253760.9671	39586.71087	373.0513	33.42341
48	0.08480918		232.434809	51.09915506	12.824489	7.116609309	5.595316734	8.781529	1.410844	7.817494	13.43504	659.4847	1000	221603.3333	34570.12	340.5153	29.07595
49	0.08480918		212.7222025	46.11095108	11.5727342	6.414581342	5.106204756	8.140434	1.311257	7.250825	12.19074	689.1801	1000	193587.3055	30199.61965	310.8199	25.2942
50	0.12742539		185.6159922	39.64481353	9.950076851	5.508881313	4.441133872	7.195796	1.162269	6.413202	10.53901	729.5288	1000	161936.674	25262.12114	270.4712	20.97793
51	0.12742539		161.9638014	34.08561906	8.55503139	4.731435941	3.861709683	6.357732	1.029721	5.669653	9.108393	764.6369	1000	135650.7135	21161.5113	235.3631	17.39838
52	0.12742539		141.3255004	29.30614427	7.355656742	4.064012042	3.357060681	5.614701	0.911876	5.010039	7.8698	795.1852	1000	113816.0432	17755.30274	204.8148	14.4298
53	0.12742539		123.3170429	25.19701157	6.32449629	3.490982162	2.91766952	4.956333	0.807167	4.425241	6.79787	821.7662	1000	95673.726	14925.10126	178.2338	11.96787
54	0.12742539		107.6033203	21.66418671	5.437947887	2.998949571	2.53520862	4.373324	0.714185	3.907075	5.870513	844.8953	1000	80593.49892	12572.58583	155.1047	9.926101
55	0.19078207		87.07453555	17.25425593	4.331192131	2.386424232	2.041772596	3.580257	0.586414	3.200665	4.696567	874.8479	1000	63901.00167	9968.556261	125.1521	7.633833
56	0.19078207		70.46227499	13.74212426	3.449730518	1.899136375	1.643965856	2.929687	0.481288	2.620807	3.756435	899.0146	1000	51009.5858	7957.495385	100.9854	5.871014
57	0.19078207		57.01933597	10.94499152	2.747692786	1.511446853	1.323346711	2.3963	0.394841	2.145084	3.003788	918.5132	1000	41037.14411	6401.794482	81.48683	4.515338
58	0.19078207		46.14106875	8.717280604	2.188549763	1.202973811	1.06500934	1.959212	0.323789	1.754994	2.401414	934.2457	1000	33306.52085	5195.817252	65.75429	3.472755
59	0.19078207		37.33817991	6.943057506	1.743210751	0.957512562	0.85691048	1.601214	0.26542	1.435279	1.919444	946.9398	1000	27298.04619	4258.495206	53.06023	2.670945
60	0.21710356		29.23192805	5.34724325	1.342624619	0.736974765	0.666768114	1.265986	0.210505	1.135586	1.483387	958.579	1000	22160.39056	3457.020927	41.421	1.98734
61	0.21710356		22.88557235	4.118259634	1.034105441	0.567263102	0.518700169	1.00055	0.166888	0.898122	1.146172	967.6644	1000	18264.49218	2849.26078	32.33563	1.478723
62	0.21710356		17.91703307	3.171773604	0.796490325	0.436655418	0.403425517	0.790469	0.13226	0.710049	0.885455	974.7564	1000	15292.53066	2385.634783	25.24361	1.100295
63	0.21710356		14.02718137	2.442841973	0.613481696	0.336135429	0.313703106	0.624273	0.10478	0.56116	0.683927	980.2925	1000	13008.81421	2029.375017	19.70748	0.818727
64	0.21710356		10.98183033	1.881452904	0.472528593	0.258767456	0.243885332	0.492848	0.082981	0.443337	0.528183	984.6142	1000	11238.55706	1753.214901	15.38581	0.609224
65	0.45434501		5.992290517	1.002734042	0.251861873	0.137825463	0.131709047	0.272037	0.04601	0.244959	0.282538	991.638	1000	8949.075095	1396.055715	8.361965	0.315688
														Scosted	Scosteddollar	Slifed	Sqalyd
													Per patient	71708.43215	11186.51542	41.89462	15.3308

Probabilitics Analysis

Markov States

Cycle	Mortality Rate	Cohort Size	Normotensive	Prehypertension	Hypertension L1	Hypertension L2	Hypertension L3	MI	CHF	Stroke	ESRD	Dead	Cohort Check	cost (¥)	Cost($)	Lifeyears	QALYs
		1000															
0	0.00116097		646.4228774	114.3337502	187.5073504	42.58932197	9.146700019	0	0	0	0	0	1000	325677.3101	50805.66038	1000	885.3333
1	0.00116097		645.6724013	135.9621188	156.284345	39.44238569	8.999956371	5.63551	0.884875	3.72015	2.237291	1.160968	1000	1149942.264	179390.9932	998.839	883.6015
2	0.00116097		644.9227965	151.4028265	132.0737152	36.55827964	8.850128064	10.60486	1.698955	7.041392	3.665419	3.181626	1000	1780608.343	277774.9015	996.8184	881.5702
3	0.00116097		644.174062	162.1214467	113.2335514	33.92517453	8.697459642	15.00704	2.453203	10.02671	4.534904	5.826451	1000	2269714.231	354075.4201	994.1735	879.3163
4	0.00116097		643.4261967	169.2406611	98.50831172	31.52800095	8.542310234	18.92184	3.156293	12.72618	5.023064	8.927134	1000	2654782.814	414146.1189	991.0729	876.8935
5	0.00175587		642.2964246	173.5199681	86.87923647	29.33120605	8.38002183	22.40284	3.813243	15.1723	5.251709	12.95305	1000	2961354.603	461971.318	987.047	873.8182
6	0.00175587		641.1686363	175.7140794	77.68440407	27.33868668	8.216319502	25.51018	4.430664	17.40196	5.31081	17.22425	1000	3209995.647	500759.3209	982.7757	870.642
7	0.00175587		640.0428282	176.3319554	70.35781445	25.53268405	8.051663901	28.28833	5.013068	19.44168	5.261188	21.67878	1000	3414799.404	532708.7071	978.3212	867.3842
8	0.00175587		638.918997	175.7634247	64.46681654	23.89603272	7.886502754	30.77429	5.564116	21.31315	5.14457	26.27211	1000	3585904.554	559401.1104	973.7279	864.0585
9	0.00175587		637.797139	174.3070998	59.68063642	22.41246801	7.721260079	32.99914	6.086806	23.03422	4.989229	30.972	1000	3730616.928	581976.2408	969.028	860.6754
10	0.0026112		636.1317246	172.0426408	55.69528916	21.04763439	7.549725449	34.96105	6.578407	24.60006	4.809755	36.58372	1000	3851163.965	600781.5785	963.4163	856.5067
11	0.0026112		634.470659	169.2981071	52.37512647	19.80900121	7.379150934	36.70741	7.045346	26.04014	4.62303	42.25203	1000	3954229.377	616859.7828	957.748	852.3012
12	0.0026112		632.8139308	166.2089341	49.57042094	18.68341866	7.209859433	38.25826	7.489422	27.36537	4.436881	47.9635	1000	4042669.93	630656.5091	952.0365	848.0642
13	0.0026112		631.1615286	162.8786389	47.16667577	17.65895198	7.042137787	39.63105	7.912172	28.58508	4.256036	53.70772	1000	4118604.373	642502.2822	946.2923	843.8005
14	0.0026112		629.5134412	159.3862831	45.07628702	16.72482863	6.876238037	40.84113	8.314918	29.70736	4.08315	59.47637	1000	4183628.944	652646.1153	940.5236	839.5139
15	0.00418182		626.8809277	155.541844	43.16138301	15.84509997	6.701579313	41.83792	8.685756	30.69259	3.9131	66.7398	1000	4232650.585	660293.4913	933.2602	833.8897
16	0.00418182		624.2594229	151.6532755	41.44726653	15.04008938	6.529681745	42.69445	9.037574	31.59051	3.753237	73.9945	1000	4272790.086	666555.2535	926.0055	828.2656
17	0.00418182		621.6488808	147.7557143	39.89357541	14.30171385	6.360693794	43.42224	9.371361	32.40704	3.603389	81.23539	1000	4304868.467	671559.4808	918.7646	822.6448
18	0.00418182		619.0492556	143.8756049	38.46933917	13.62278675	6.19473718	44.03169	9.688009	33.14754	3.463067	88.45797	1000	4329569.56	675412.8513	911.542	817.0306
19	0.00418182		616.4605015	140.0327313	37.15074782	12.99693018	6.031909524	44.53223	9.988326	33.81683	3.331618	95.65817	1000	4347478.294	678206.6138	904.3418	811.426
20	0.0061857		612.6472611	135.9611621	35.84500691	12.39244805	5.860199655	44.84324	10.25304	34.35154	3.201649	104.6445	1000	4350715.989	678711.6943	895.3555	804.2078
21	0.0061857		608.8576082	131.9680228	34.61746353	11.83275228	5.692415572	45.06038	10.50174	34.82119	3.079618	113.5688	1000	4348102.726	678304.0252	886.4312	797.0304
22	0.0061857		605.0913971	128.0606468	33.45666095	11.3131563	5.528575517	45.19144	10.73516	35.22986	2.964785	122.4283	1000	4340090.862	677054.1745	877.5717	789.8963
23	0.0061857		601.3484825	124.2440028	32.35366131	10.82952378	5.368682496	45.24358	10.95397	35.58135	2.856468	131.2203	1000	4327096.615	675027.0719	868.7797	782.8077
24	0.0061857		597.6287206	120.5212546	31.30144837	10.37820486	5.212726273	45.22337	11.15881	35.87918	2.754045	139.9422	1000	4309506.363	672282.9926	860.0578	775.7667
25	0.01030714		591.4688804	116.3974705	30.16546657	9.91320593	5.039201243	44.95053	11.30429	35.97875	2.645613	152.1366	1000	4270578.974	666210.3199	847.8634	765.578
26	0.01030714		585.3725305	112.4020581	29.07912845	9.478114506	4.870901568	44.61853	11.43557	36.02955	2.542878	164.1707	1000	4228005.449	659568.85	835.8293	755.5116
27	0.01030714		579.3390166	108.5335816	28.03830928	9.070074491	4.707731151	44.23334	11.55336	36.03507	2.445345	176.0442	1000	4182150.952	652415.5485	823.9558	745.5679
28	0.01030714		573.3676909	104.7900178	27.03960762	8.686566667	4.549587843	43.80044	11.65831	35.99853	2.352577	187.7567	1000	4133356.477	644803.6105	812.2433	735.7476
29	0.01030714		567.4579124	101.1689182	26.08017907	8.325366649	4.396364714	43.32487	11.75107	35.92297	2.264193	199.3081	1000	4081940.545	636782.7251	800.6919	726.0508
30	0.01720644		557.6939806	96.96953696	24.97767506	7.927068724	4.217619283	42.51239	11.75116	35.56338	2.164235	216.223	1000	4001141.708	624178.1064	783.777	711.4684
31	0.01720644		548.0980514	92.94004134	23.92392431	7.55247096	4.04582726	41.67535	11.73966	35.17353	2.069303	232.7818	1000	3919224.735	611399.0586	767.2182	697.1764
32	0.01720644		538.6672339	89.07438791	22.91625126	7.199593397	3.880753592	40.81832	11.71736	34.75657	1.979045	248.9905	1000	3836481.934	598491.1817	751.0095	683.1705
33	0.01720644		529.3986873	85.36657614	21.9522491	6.866677025	3.722166786	39.94547	11.68498	34.31546	1.893149	264.8546	1000	3753180.262	585496.1209	735.1454	669.4463
34	0.01720644		520.2896194	81.81069118	21.02972947	6.552155431	3.569839501	39.06054	11.64323	33.85291	1.811337	280.38	1000	3669563.024	572451.8318	719.62	655.9993
35	0.03063911		504.3484088	77.30199897	19.86419875	6.166617309	3.375596553	37.64226	11.43635	32.91673	1.709025	305.2388	1000	3538708.208	552038.4805	694.7612	634.0134
36	0.03063911		488.8956227	73.04014149	18.76380244	5.805999301	3.191746965	36.24944	11.22484	31.98305	1.61274	329.2326	1000	3410841.97	532091.3474	670.7674	612.768
37	0.03063911		473.9162962	69.01190807	17.72476581	5.468366647	3.017755975	34.88452	11.00954	31.05435	1.522084	352.3904	1000	3286087.773	512629.6926	647.6096	592.2392
38	0.03063911		459.395923	65.20473544	16.74356716	5.151977414	2.853113584	33.5495	10.79125	30.13287	1.436691	374.7404	1000	3164542.58	493668.6425	625.2596	572.4035
39	0.03063911		445.3204411	61.60668877	15.81691232	4.85525865	2.697333787	32.246	10.57068	29.22055	1.356227	396.3099	1000	3046279.628	475219.622	603.6901	553.2382
40	0.04952348		423.266621	57.04303641	14.6430211	4.485097344	2.499016314	30.36639	10.14887	27.7673	1.254773	428.5259	1000	2876844.725	448787.7771	571.4741	524.2736
41	0.04952348		402.304983	52.8168315	13.55638291	4.144137381	2.315186477	28.58011	9.738208	26.3709	1.161003	459.0123	1000	2716147.337	423718.9846	540.9877	496.8326
42	0.04952348		382.3814383	48.90323991	12.55047881	3.82990959	2.144798611	26.88439	9.338985	25.03077	1.074316	487.8617	1000	2563870.753	399963.8375	512.1383	470.8351
43	0.04952348		363.444577	45.27923179	11.61928587	3.54018056	1.986880728	25.27632	8.951416	23.74614	0.994165	515.1618	1000	2419692.892	377472.0912	484.8382	446.2056
44	0.04952348		345.4455351	41.9234554	10.75723704	3.272926036	1.840529591	23.75282	8.575637	22.51603	0.920043	540.9958	1000	2283289.223	356193.1187	459.0042	422.872
45	0.08480918		316.148581	37.33682014	9.579608174	2.910820381	1.639961703	21.47264	7.909121	20.54482	0.819025	581.6386	1000	2079412.269	324388.314	418.3614	385.845
46	0.08480918		289.3362777	33.25176821	8.530928392	2.589158239	1.461204816	19.40204	7.290836	18.73682	0.729131	618.6718	1000	1894406.807	295527.4619	381.3282	352.0678
47	0.08480918		264.797904	29.61349537	7.597069526	2.303343641	1.30189468	17.52309	6.71775	17.07978	0.64913	652.4165	1000	1726624.917	269353.487	347.5835	321.2548
48	0.08480918		242.3406098	26.37317479	6.765453759	2.049319038	1.159922022	15.81921	6.186959	15.56218	0.577927	683.1653	1000	1574548.183	245629.5165	316.8347	293.1455
49	0.08480918		221.7879003	23.48730754	6.024882783	1.823498985	1.033405271	14.27506	5.695691	14.17321	0.51455	711.1845	1000	1436779.736	224137.6388	288.8155	267.5024
50	0.12742539		193.5264899	19.91620307	5.108628335	1.545002002	0.876626356	12.26815	4.998578	12.2988	0.436208	749.0253	1000	1257623.41	196189.252	250.9747	232.7088
51	0.12742539		168.8663009	16.8879999	4.331722779	1.309157649	0.743614166	10.53897	4.384919	10.66755	0.369803	781.9	1000	1103266.125	172109.5155	218.1	202.4467
52	0.12742539		147.3484461	14.3201793	3.672971295	1.109403805	0.630769607	9.049873	3.845034	9.248732	0.313514	810.4611	1000	970336.1641	151372.4416	189.5389	176.1252
53	0.12742539		128.5725124	12.14276078	3.114403358	0.940195364	0.535037816	7.768197	3.370311	8.015357	0.265799	835.2754	1000	855909.1186	133521.8225	164.7246	153.2306
54	0.12742539		112.1891095	10.29639805	2.640782046	0.79684477	0.453826037	6.665584	2.953103	6.943743	0.225349	856.8353	1000	757450.6091	118162.295	143.1647	133.3163
55	0.19078207		90.78543842	8.078418335	2.071876907	0.624902567	0.356180935	5.295151	2.399523	5.573204	0.176781	884.6385	1000	634911.2544	99046.15569	115.3615	107.5474
56	0.19078207		73.46520411	6.33820629	1.625532503	0.490089783	0.279539321	4.20482	1.948945	4.471297	0.138683	907.0377	1000	537375.6184	83830.59646	92.96232	86.76272
57	0.19078207		59.44936004	4.97285217	1.275344854	0.384379855	0.219384892	3.337742	1.582377	3.585804	0.108797	925.084	1000	459772.6378	71724.53149	74.91604	69.99758
58	0.19078207		48.10748778	3.901611206	1.000598481	0.30148415	0.172172053	2.648509	1.284292	2.874558	0.085352	939.6239	1000	398052.2536	62096.15157	60.37606	56.47419
59	0.19078207		38.92944144	3.06113013	0.785040823	0.236474794	0.135117388	2.100874	1.042	2.303528	0.06696	951.3394	1000	348981.6624	54441.13933	48.66057	45.56529
60	0.21710356		30.47772104	2.321128068	0.595257276	0.179265265	0.102479413	1.610624	0.817711	1.784639	0.050769	962.0604	1000	305006.5856	47581.02735	37.93959	35.56576
61	0.21710356		23.86089924	1.760013107	0.451354047	0.135900457	0.077723996	1.234357	0.641484	1.382123	0.038494	970.4177	1000	271229.7291	42311.83774	29.58235	27.76187
62	0.21710356		18.68061302	1.334542043	0.342239479	0.103028506	0.058947741	0.945683	0.503073	1.070012	0.029187	976.9327	1000	245294.7151	38265.97555	23.06732	21.67127
63	0.21710356		14.62498539	1.011924799	0.259503363	0.078109536	0.04470676	0.724293	0.394404	0.828098	0.02213	982.0118	1000	225387.311	35160.42051	17.98815	16.91761
64	0.21710356		11.44984897	0.767297694	0.196768683	0.059218794	0.033905768	0.554565	0.309117	0.640666	0.016779	985.9718	1000	210111.3142	32777.36501	14.02817	13.20726
65	0.45434501		6.247667222	0.399772619	0.102518393	0.030848408	0.017670092	0.292923	0.168868	0.343507	0.008742	992.3875	1000	186495.9477	29093.36784	7.612516	7.177805
														Scostp	Scostp($)	Slifep	Sqalyp
													Per patient	175257.4678	27340.16497	40.54374	36.52414

Usual practice

Deterministic Cohort Analysis

			Markov States														
**Cycle**	**Mortality Rate**	**Cohort Size**	Normotensive	Prehypertension	Hypertension L1	Hypertension L2	Hypertension L3	MI	CHF	Stroke	ESRD	Dead	Cohort Check	Cost(￥)	Cost($)	Lifeyears	QALYs
		1000															
0	0.00116097		610	126.1115602	206.8229588	46.97655618	10.08892482	0	0	0	0	0	1000	401142.4553	62578.22302	1000	866.5011
1	0.00116097		609.2918097	154.3365235	172.6695671	45.9474529	10.42652035	0.844479	0.118455	0.753536	4.450688	1.160968	1000	935673.4298	145965.055	998.839	823.9064
2	0.00116097		608.5844416	175.1308644	146.3882862	44.90158915	10.74602114	1.645242	0.232021	1.464397	8.333672	2.573465	1000	1353540.99	211152.3944	997.4265	783.3089
3	0.00116097		607.8778947	190.2572189	126.1194687	43.85517147	11.0485375	2.409452	0.341487	2.140027	11.74527	4.205476	1000	1678166.386	261793.9563	995.7945	744.6276
4	0.00116097		607.1721681	201.0616546	110.4434119	42.81977341	11.33501183	3.14264	0.447466	2.786164	14.76131	6.030404	1000	1927750.31	300729.0483	993.9696	707.7826
5	0.00175587		606.1060534	208.4525652	98.21057031	41.77801546	11.59951309	3.847219	0.550162	3.405587	17.43308	8.617233	1000	2115469.034	330013.1693	991.3828	672.2949
6	0.00175587		605.0418106	213.3258466	88.67371999	40.76208636	11.8491763	4.527549	0.650086	4.002654	19.8141	11.35297	1000	2253485.405	351543.7232	988.647	638.5298
7	0.00175587		603.9794365	216.2856925	81.19635909	39.77549396	12.08465855	5.186273	0.747528	4.580077	21.94337	14.22111	1000	2350891.526	366739.078	985.7789	606.4106
8	0.00175587		602.9189278	217.7932604	75.29292404	38.82030386	12.30655249	5.825466	0.842717	5.139979	23.85255	17.20732	1000	2415006.218	376740.97	982.7927	575.8632
9	0.00175587		601.8602812	218.2005624	70.59298935	37.89752517	12.51539934	6.446771	0.935833	5.684029	25.56752	20.29909	1000	2451772.176	382476.4595	979.7009	546.8162
10	0.0026112		600.2887051	217.5896885	66.7535782	36.97498677	12.70099409	7.045984	1.026218	6.208688	27.08784	24.32332	1000	2464058.368	384393.1054	975.6767	518.7552
11	0.0026112		598.7212327	216.3533175	63.62598738	36.08635128	12.87418083	7.628643	1.114633	6.718972	28.45065	28.42603	1000	2457851.864	383424.8907	971.574	492.1046
12	0.0026112		597.1578533	214.6497025	61.04390496	35.23084902	13.03542895	8.19565	1.201176	7.215767	29.67147	32.5982	1000	2436530.268	380098.7217	967.4018	466.7972
13	0.0026112		595.5985562	212.5995279	58.88056892	34.40745354	13.18518285	8.747749	1.285928	7.699798	30.76357	36.83166	1000	2402911.627	374854.2138	963.1683	442.7686
14	0.0026112		594.0433307	210.2948294	57.03935239	33.61497401	13.32386545	9.285567	1.368957	8.171669	31.73842	41.11903	1000	2359358.665	368059.9518	958.881	419.957
15	0.00418182		591.5591469	207.4754996	55.35700266	32.79932677	13.43095426	9.795054	1.448172	8.61905	32.55617	46.95962	1000	2304332.72	359475.9044	953.0404	397.6753
16	0.00418182		589.0853516	204.5336902	53.87208143	32.01445385	13.52739457	10.28963	1.525521	9.053783	33.27285	52.82524	1000	2243199.52	349939.1251	947.1748	376.5606
17	0.00418182		586.6219012	201.5108029	52.54083177	31.25887174	13.61360868	10.76978	1.601055	9.476295	33.89676	58.7101	1000	2177407.356	339675.5476	941.2899	356.5539
18	0.00418182		584.1687525	198.4382765	51.32992616	30.53112063	13.6900029	11.23591	1.674823	9.886971	34.43536	64.60885	1000	2108178.539	328875.8521	935.3911	337.5988
19	0.00418182		581.7258625	195.3399613	50.21396773	29.829782	13.75696878	11.68842	1.746866	10.28615	34.89538	70.51663	1000	2036544.532	317700.947	929.4834	319.6416
20	0.0061857		578.1274802	191.8424871	49.0729676	29.09371498	13.78731672	12.10425	1.813725	10.65354	35.21299	78.29152	1000	1959489.882	305680.4216	921.7085	302.0211
21	0.0061857		574.5513564	188.3641835	47.99713946	28.38421955	13.80880185	12.50544	1.878662	11.00853	35.46229	86.03938	1000	1881917.39	293579.1128	913.9606	285.3647
22	0.0061857		570.9973535	184.9152734	46.97481215	27.69990327	13.8218293	12.89238	1.941723	11.35145	35.64878	93.75649	1000	1804444.076	281493.2759	906.2435	269.6206
23	0.0061857		567.4653345	181.503333	45.9970588	27.03945006	13.8267894	13.26546	2.002951	11.68262	35.77754	101.4395	1000	1727580.939	269502.6265	898.5605	254.7399
24	0.0061857		563.9551636	178.1339241	45.05703473	26.40161947	13.82405832	13.62503	2.06239	12.00236	35.85323	109.0852	1000	1651748.197	257672.7188	890.9148	240.676
25	0.01030714		558.1424013	174.0769083	43.96377575	25.67643225	13.75702364	13.9153	2.111578	12.26148	35.73239	120.3627	1000	1570807.673	245045.9969	879.6373	226.4401
26	0.01030714		552.389552	170.0997299	42.90716245	24.97713451	13.6833275	14.19011	2.158615	12.5074	35.56716	131.5198	1000	1492125.392	232771.5612	868.4802	213.0431
27	0.01030714		546.6959981	166.2035682	41.88363965	24.30241359	13.60339155	14.44998	2.203562	12.74052	35.36178	142.5551	1000	1415896.99	220879.9304	857.4449	200.4361
28	0.01030714		541.0611284	162.3888985	40.89039894	23.65104047	13.51761899	14.69538	2.24648	12.96126	35.12017	153.4676	1000	1342265.901	209393.4805	846.5324	188.573
29	0.01030714		535.484338	158.6556671	39.9251979	23.02186474	13.42639535	14.92677	2.287425	13.17001	34.84593	164.2564	1000	1271331.46	198327.7077	835.7436	177.4104
30	0.01720644		526.2705577	153.9088109	38.71076742	22.25497461	13.23745629	15.04164	2.310673	13.27627	34.30198	180.6869	1000	1194805.92	186389.7235	819.3131	165.7414
31	0.01720644		517.2153138	149.3001478	37.53593433	21.51768297	13.04575478	15.14109	2.331631	13.36919	33.73765	196.8056	1000	1122020.736	175035.2348	803.1944	154.8389
32	0.01720644		508.3158783	144.8264807	36.39880193	20.80853495	12.851707	15.22586	2.350396	13.44938	33.15626	212.6167	1000	1052916.835	164255.0263	787.3833	144.6528
33	0.01720644		499.5695705	140.4845069	35.29770634	20.12616792	12.65570353	15.29663	2.367059	13.51741	32.56083	228.1244	1000	987414.29	154036.6292	771.8756	135.1362
34	0.01720644		490.9737556	136.2708669	34.231165	19.4693047	12.45811066	15.35406	2.381711	13.57386	31.9541	243.3331	1000	925416.8437	144365.0276	756.6669	126.2453
35	0.03063911		475.930757	130.3516999	32.73802216	18.57522262	12.09192591	15.19257	2.362446	13.43694	30.90934	268.4111	1000	854982.4819	133377.2672	731.5889	116.324
36	0.03063911		461.3486624	124.6885464	31.31080455	17.72482858	11.73240596	15.02056	2.341427	13.29072	29.88031	292.6617	1000	789476.6641	123158.3596	707.3383	107.1824
37	0.03063911		447.2133503	119.2706175	29.94644605	16.91574218	11.3797507	14.83912	2.318811	13.13613	28.86892	316.1111	1000	728618.3349	113664.4602	683.8889	98.7592
38	0.03063911		433.5111315	114.0875212	28.64206231	16.14573463	11.03412542	14.64922	2.294743	12.97402	27.87675	338.7847	1000	672132.2591	104852.6324	661.2153	90.99805
39	0.03063911		420.2287366	109.1292611	27.39493012	15.4127174	10.69566372	14.45181	2.269362	12.8052	26.9051	360.7072	1000	619750.9052	96681.14121	639.2928	83.847
40	0.04952348		399.4175452	102.3253926	25.68513492	14.42367221	10.16248921	13.97485	2.199941	12.38861	25.44697	393.9754	1000	560075.7399	87371.81543	606.0246	75.75013
41	0.04952348		379.6369965	95.94558536	24.08237338	13.49967863	9.652983877	13.50491	2.13127	11.97792	24.05676	425.5115	1000	505944.1694	78927.29043	574.4885	68.43541
42	0.04952348		360.8360495	89.96350742	22.57990063	12.63627862	9.166358923	13.04279	2.063479	11.57384	22.73265	455.4051	1000	456873.9471	71272.33574	544.5949	61.82731
43	0.04952348		342.9661909	84.3544401	21.17140686	11.82933987	8.701825409	12.58914	1.996683	11.17694	21.47271	483.7413	1000	412419.3335	64337.41602	516.2587	55.85757
44	0.04952348		325.98131	79.09518353	19.85098647	11.07502977	8.258597796	12.14452	1.930978	10.78772	20.27485	510.6008	1000	372169.2967	58058.41029	489.3992	50.46455
45	0.08480918		298.335101	71.37303801	17.91265435	9.979001543	7.544486615	11.28087	1.798315	10.02595	18.42154	553.329	1000	323247.7455	50426.6483	446.671	43.89663
46	0.08480918		273.0335444	64.40498818	16.16376301	8.99230211	6.890245525	10.47292	1.673857	9.312938	16.73155	592.3239	1000	280672.3509	43784.88674	407.6761	38.18382
47	0.08480918		249.8777922	58.11741287	14.58577638	8.103898044	6.291082837	9.717788	1.557208	8.646184	15.1914	627.9115	1000	243636.447	38007.28573	372.0885	33.21476
48	0.08480918		228.6858605	52.44386967	13.16197556	7.303888501	5.742561911	9.012622	1.447972	8.023218	13.7886	660.3894	1000	211432.6759	32983.49744	339.6106	28.8926
49	0.08480918		209.2911992	47.32439716	11.87727983	6.583386114	5.240578565	8.354656	1.345764	7.441636	12.51155	690.0296	1000	183441.3996	28616.85834	309.9704	25.13311
50	0.12742539		182.6221859	40.6880981	10.21192098	5.653851874	4.558005816	7.385159	1.192855	6.58197	10.81635	730.2896	1000	151683.5857	23662.63937	269.7104	20.84295
51	0.12742539		159.351482	34.98260904	8.780163795	4.855947414	3.963333622	6.525041	1.056819	5.818855	9.348088	765.3177	1000	125397.0599	19561.94134	234.6823	17.2853
52	0.12742539		139.0460568	30.07735859	7.549226657	4.170959728	3.445404383	5.762457	0.935873	5.141882	8.0769	795.7939	1000	103646.0506	16168.7839	204.2061	14.33507
53	0.12742539		121.3280584	25.86009082	6.490930403	3.582850114	2.994450297	5.086763	0.828408	4.541695	6.976761	822.31	1000	85653.21598	13361.90169	177.69	11.88852
54	0.12742539		105.8677828	22.23429689	5.581051779	3.077869296	2.601924637	4.488412	0.732979	4.009893	6.025	845.3808	1000	70773.19984	11040.61917	154.6192	9.85965
55	0.19078207		85.67010756	17.70831529	4.445170872	2.44922487	2.095503453	3.674474	0.601846	3.284893	4.82016	875.2503	1000	54210.054	8456.768424	124.7497	7.582198
56	0.19078207		69.32578669	14.10375911	3.5405129	1.949113648	1.687228115	3.006785	0.493954	2.689776	3.855289	899.3478	1000	41520.94048	6477.266715	100.6522	5.830897
57	0.19078207		56.09966926	11.23301761	2.820000491	1.55122177	1.358171624	2.45936	0.405231	2.201534	3.082835	918.789	1000	31801.82816	4961.085193	81.21104	4.484174
58	0.19078207		45.39685796	8.946682725	2.246143178	1.234631017	1.093035902	2.01077	0.33231	1.801179	2.464609	934.4738	1000	24358.96938	3799.999224	65.52622	3.44855
59	0.19078207		36.73595121	7.125769545	1.789084718	0.982710261	0.879460756	1.643351	0.272404	1.47305	1.969955	947.1283	1000	18660.18835	2910.989382	52.87174	2.652147
60	0.21710356		28.76044534	5.487960178	1.377956846	0.756368838	0.684314644	1.299302	0.216045	1.16547	1.522424	958.7297	1000	13832.86589	2157.927079	41.27029	1.973214
61	0.21710356		22.5164502	4.226634888	1.061318742	0.582191078	0.532350173	1.02688	0.17128	0.921756	1.176335	967.7848	1000	10258.76258	1600.366962	32.2152	1.46811
62	0.21710356		17.62804867	3.25524133	0.817450597	0.448146351	0.414041978	0.811271	0.135741	0.728735	0.908757	974.8526	1000	7612.7046	1187.581918	25.14743	1.092322
63	0.21710356		13.80093651	2.507127288	0.629625951	0.344981099	0.321958451	0.640701	0.107537	0.575927	0.701925	980.3693	1000	5653.759926	881.9865484	19.63072	0.812739
64	0.21710356		10.80470403	1.930964822	0.484963556	0.265577126	0.250303368	0.505817	0.085165	0.455004	0.542083	984.6754	1000	4203.459507	655.7396831	15.32458	0.604726
65	0.45434501		5.89564067	1.02912178	0.258489817	0.141452449	0.135175074	0.279196	0.047221	0.251406	0.289973	991.6723	1000	2202.81607	343.6393069	8.327675	0.313327
														pcosted	pcosteddollar	plifed	pqalyd
													Per patient	70239.79712	10957.40835	41.85301	15.26263

Probabilitics Analysis

			Markov States														
**Cycle**	**Mortality Rate**	**Cohort Size**	Normotensive	Prehypertension	Hypertension L1	Hypertension L2	Hypertension L3	MI	CHF	Stroke	ESRD	Dead	Cohort Check	Cost(￥)	Cost($)	Lifeyears	QALYs
		1000															
0	0.00116097		632.4289811	118.8588582	194.9285274	44.27492467	9.508708655	0	0	0	0	0	1000	174770.0013	27264.1202	1000	880.795
1	0.00116097		631.6947514	141.3432356	162.4697761	41.00343877	9.356157177	5.858552	0.919897	3.867386	2.325838	1.160968	1000	1031657.762	160938.6109	998.839	879.0406
2	0.00116097		630.9613742	157.3950565	137.3009365	38.0051854	9.200398957	11.02458	1.766196	7.320076	3.810489	3.215704	1000	1687284.381	263216.3634	996.7843	876.9748
3	0.00116097		630.2288485	168.5378989	117.7151157	35.2678671	9.041688215	15.60099	2.550296	10.42355	4.714387	5.919364	1000	2195748.131	342536.7085	994.0806	874.6775
4	0.00116097		629.4971731	175.938878	102.4070795	32.77581805	8.880398295	19.67073	3.281213	13.22986	5.221867	9.096978	1000	2596056.98	404984.889	990.903	872.2047
5	0.00175587		628.3918586	180.3875517	90.31774805	30.49207826	8.711686831	23.2895	3.964164	15.77279	5.459562	13.21306	1000	2914762.286	454702.9166	986.7869	869.0768
6	0.00175587		627.2884849	182.6685017	80.75900202	28.42069885	8.541505482	26.51983	4.606021	18.0907	5.521002	17.58426	1000	3173244.06	495026.0734	982.4157	865.844
7	0.00175587		626.1870486	183.310832	73.1424402	26.54321813	8.370333133	29.40793	5.211475	20.21115	5.469416	22.14616	1000	3386153.553	528239.9543	977.8538	862.5261
8	0.00175587		625.0875462	182.7198	67.01828802	24.84179132	8.198635227	31.99227	5.784332	22.15668	5.348182	26.85247	1000	3564030.712	555988.7911	973.1475	859.1376
9	0.00175587		623.9899744	181.2058366	62.04268019	23.29950999	8.026852568	34.30519	6.32771	23.94587	5.186693	31.66969	1000	3714470.522	579457.4014	968.3303	855.6894
10	0.0026112		622.3606133	178.8517546	57.89960062	21.88065891	7.848528931	36.34474	6.838768	25.57368	5.000115	37.40154	1000	3839788.576	599007.0178	962.5985	851.4576
11	0.0026112		620.7355068	175.9985977	54.44803233	20.59300304	7.671203407	38.16022	7.324187	27.07076	4.806001	43.19249	1000	3946933.117	615721.5663	956.8075	847.1873
12	0.0026112		619.1146438	172.7871611	51.532322	19.42287212	7.495211677	39.77245	7.785839	28.44844	4.612484	49.02858	1000	4038873.976	630064.3402	950.9714	842.8842
13	0.0026112		617.4980131	169.3250594	49.03344126	18.35785902	7.320851934	41.19957	8.22532	29.71643	4.424482	54.89897	1000	4117813.756	642378.946	945.101	838.5528
14	0.0026112		615.8856038	165.6944828	46.86031898	17.38676489	7.148386193	42.45754	8.644007	30.88312	4.244753	60.79503	1000	4185411.874	652924.2523	939.205	834.1975
15	0.00418182		613.3100796	161.6978883	44.86962678	16.47221828	6.966814817	43.49378	9.029522	31.90734	4.067973	68.18475	1000	4236373.696	660874.2966	931.8152	828.5119
16	0.00418182		610.7453257	157.6554179	43.0876689	15.63534692	6.788113876	44.38421	9.395264	32.8408	3.901783	75.56607	1000	4278101.841	667383.8872	924.4339	822.8257
17	0.00418182		608.1912971	153.6035987	41.47248571	14.86774793	6.61243771	45.1408	9.742262	33.68965	3.746004	82.93371	1000	4311449.822	672586.1722	917.0663	817.1423
18	0.00418182		605.6479491	149.5699221	39.99188095	14.16195021	6.439912855	45.77438	10.07144	34.45946	3.600128	90.28298	1000	4337128.536	676592.0515	909.717	811.4651
19	0.00418182		603.1152369	145.574955	38.62110232	13.51132345	6.270640797	46.29473	10.38364	35.15524	3.463477	97.60965	1000	4355746.063	679496.3858	902.3904	805.7971
20	0.0061857		599.3845463	141.342241	37.26368271	12.88291709	6.092134985	46.61805	10.65883	35.71111	3.328364	106.7181	1000	4359111.9	680021.4564	893.2819	798.5267
21	0.0061857		595.6769326	137.191061	35.98755556	12.30106965	5.917710333	46.84378	10.91738	36.19934	3.201503	115.7637	1000	4356395.209	679597.6525	884.2363	791.2972
22	0.0061857		591.9922532	133.1290387	34.78081066	11.76090907	5.747385806	46.98003	11.16003	36.62419	3.082126	124.7432	1000	4348066.251	678298.3352	875.2568	784.1113
23	0.0061857		588.3303661	129.1613393	33.63415643	11.25813531	5.58116453	47.03423	11.38751	36.98959	2.969521	133.654	1000	4334557.716	676191.0037	866.346	776.9713
24	0.0061857		584.6911303	125.291252	32.54029895	10.78895406	5.419035862	47.01322	11.60046	37.29921	2.863045	142.4934	1000	4316271.277	673338.3192	857.5066	769.8794
25	0.01030714		578.6646396	121.0042565	31.35935719	10.30555138	5.238643048	46.72958	11.75169	37.40271	2.750321	154.7932	1000	4275803.217	667025.3018	845.2068	759.6647
26	0.01030714		572.7002648	116.8507134	30.23002392	9.853239884	5.063682399	46.38445	11.88817	37.45553	2.64352	166.9304	1000	4231544.714	660120.9754	833.0696	749.5731
27	0.01030714		566.7973656	112.8291301	29.14801115	9.429050437	4.894054013	45.98401	12.01062	37.46127	2.542127	178.9044	1000	4183875.386	652684.5602	821.0956	739.6053
28	0.01030714		560.9553084	108.9374033	28.10978282	9.030364117	4.729651701	45.53397	12.11973	37.42329	2.445688	190.7148	1000	4133149.72	644771.3564	809.2852	729.7619
29	0.01030714		555.173466	105.1729877	27.11238194	8.654868504	4.570364298	45.03958	12.21616	37.34473	2.353805	202.3616	1000	4079698.846	636433.0199	797.6384	720.0431
30	0.01720644		545.6209059	100.807403	25.96624296	8.24080672	4.384544469	44.19495	12.21625	36.97091	2.249892	219.3481	1000	3995702.147	623329.5349	780.6519	705.4814
31	0.01720644		536.2327113	96.61842781	24.8707868	7.851383103	4.205953252	43.32478	12.2043	36.56563	2.151202	235.9748	1000	3910543.059	610044.7172	764.0252	691.2113
32	0.01720644		527.0060541	92.59977932	23.82323201	7.484539331	4.034346288	42.43383	12.18111	36.13217	2.057372	252.2476	1000	3824525.459	596625.9715	747.7524	677.2285
33	0.01720644		517.9381549	88.74521955	22.82107651	7.138446775	3.869482924	41.52643	12.14745	35.6736	1.968077	268.1721	1000	3737926.869	583116.5915	731.8279	663.5285
34	0.01720644		509.0262819	85.04859957	21.86204535	6.811477028	3.71112682	40.60648	12.10404	35.19275	1.883026	283.7542	1000	3651000.222	569556.0347	716.2458	650.1071
35	0.03063911		493.43017	80.36146207	20.65038519	6.410680055	3.509196113	39.13206	11.88898	34.21951	1.776665	308.6209	1000	3514966.427	548334.7626	691.3791	628.227
36	0.03063911		478.311909	75.93092854	19.50643733	6.035789485	3.318070115	37.68412	11.6691	33.24887	1.67657	332.6182	1000	3382039.491	527598.1607	667.3818	607.0869
37	0.03063911		463.6568581	71.74326546	18.42627764	5.684793986	3.1371929	36.26518	11.44528	32.28342	1.582325	355.7754	1000	3252347.765	507366.2514	644.2246	586.6627
38	0.03063911		449.4508249	67.78541232	17.40624505	5.355882681	2.966034282	34.87732	11.21835	31.32547	1.493552	378.1209	1000	3125992.049	487654.7597	621.8791	566.931
39	0.03063911		435.6800519	64.04496195	16.44291502	5.047420365	2.804089023	33.52224	10.98905	30.37705	1.409904	399.6823	1000	3003048.479	468475.5628	600.3177	547.869
40	0.04952348		414.1036575	59.30068909	15.22256346	4.662608793	2.59792253	31.56824	10.55054	28.86628	1.304434	431.8231	1000	2826907.671	440997.5967	568.1769	519.1083
41	0.04952348		393.5958014	54.90721918	14.09291824	4.308154296	2.406817065	29.71125	10.12363	27.4146	1.206953	462.2327	1000	2659850.193	414936.6301	537.7673	491.8646
42	0.04952348		374.1035658	50.83873523	13.0472024	3.981489979	2.229685577	27.94842	9.708604	26.02144	1.116836	491.004	1000	2501546.797	390241.3003	508.996	466.0581
43	0.04952348		355.5766536	47.07129591	12.07915466	3.680294036	2.065517611	26.2767	9.305696	24.68597	1.033512	518.2252	1000	2351662.656	366859.3743	481.7748	441.6132
44	0.04952348		337.9672586	43.5827044	11.1829876	3.402462097	1.91337418	24.69292	8.915045	23.40717	0.956457	543.9796	1000	2209860.394	344738.2215	456.0204	418.4581
45	0.08480918		309.3045311	38.81453902	9.958750474	3.026025003	1.704868204	22.32248	8.222149	21.35794	0.85144	584.4373	1000	1997914.387	311674.6443	415.5627	381.7594
46	0.08480918		283.0726661	34.56780867	8.868565982	2.691632097	1.519036467	20.16993	7.579393	19.47839	0.757989	621.2946	1000	1805586.767	281671.5357	378.7054	348.2869
47	0.08480918		259.0655041	30.78554005	7.897746794	2.394505512	1.353421145	18.21662	6.983625	17.75577	0.674821	654.8724	1000	1631164.395	254461.6457	345.1276	317.7568
48	0.08480918		237.09437	27.41697387	7.033217289	2.130427109	1.205829484	16.4453	6.431827	16.1781	0.6008	685.4632	1000	1473068.76	229798.7265	314.5368	289.91
49	0.08480918		216.9865899	24.41688959	6.263335951	1.895669536	1.074305446	14.84003	5.921115	14.73416	0.534915	713.333	1000	1329847.705	207456.2419	286.667	264.5104
50	0.12742539		189.3369884	20.70444774	5.31081793	1.606150182	0.911321526	12.7537	5.196412	12.78556	0.453472	750.9411	1000	1143600.72	178401.7123	249.0589	230.0701
51	0.12742539		165.2106482	17.55639417	4.50316396	1.360971568	0.773044971	10.95608	4.558465	11.08975	0.384439	783.607	1000	983134.2727	153368.9465	216.393	200.1201
52	0.12742539		144.1586164	14.88694421	3.818340371	1.153311854	0.655734243	9.40805	3.997213	9.614779	0.325922	811.9811	1000	844943.2016	131811.1394	188.0189	174.0743
53	0.12742539		125.7891481	12.62334768	3.237665399	0.977406472	0.556213574	8.075647	3.503702	8.332589	0.276319	836.628	1000	725987.3558	113254.0275	163.372	151.4232
54	0.12742539		109.7604164	10.70390951	2.745299074	0.82838234	0.471787591	6.929395	3.069982	7.218563	0.234268	858.038	1000	623632.0495	97286.59972	141.962	131.7238
55	0.19078207		88.82009645	8.398146454	2.153877774	0.649634998	0.370277885	5.504723	2.494492	5.793781	0.183778	885.6312	1000	496242.825	77413.8807	114.3688	106.2455
56	0.19078207		71.87481416	6.589060194	1.689867924	0.509486586	0.290602945	4.371239	2.02608	4.648262	0.144172	907.8564	1000	394846.9178	61596.11917	92.14358	85.69861
57	0.19078207		58.16238798	5.169668008	1.325820528	0.399592864	0.228067721	3.469844	1.645005	3.727723	0.113103	925.7588	1000	314172.5618	49010.91964	74.24121	69.12809
58	0.19078207		47.06604692	4.056029406	1.040200227	0.313416308	0.178986288	2.753332	1.335122	2.988327	0.08873	940.1798	1000	250009.4047	39001.46713	59.82019	55.76388
59	0.19078207		38.08668883	3.18228372	0.816111215	0.245834007	0.140465072	2.184023	1.08324	2.394697	0.06961	951.797	1000	198996.6946	31043.48436	48.20295	44.98517
60	0.21710356		29.81793302	2.412993812	0.618816403	0.186360236	0.106535349	1.67437	0.850075	1.855271	0.052779	962.4249	1000	153281.1697	23911.86247	37.57513	35.10734
61	0.21710356		23.34435355	1.82967101	0.469217764	0.141279134	0.080800161	1.28321	0.666872	1.436824	0.040017	970.7078	1000	118167.4908	18434.12856	29.29225	27.39972
62	0.21710356		18.27621124	1.387360627	0.355784653	0.107106174	0.061280778	0.983111	0.522983	1.112361	0.030342	977.1635	1000	91206.01918	14228.13899	22.83654	21.38523
63	0.21710356		14.30838068	1.051974819	0.269774002	0.08120096	0.046476167	0.752959	0.410013	0.860872	0.023006	982.1953	1000	70510.71867	10999.67211	17.80466	16.69174
64	0.21710356		11.20198026	0.797665848	0.204556405	0.061562559	0.035247693	0.576514	0.321351	0.666022	0.017444	986.1177	1000	54630.12754	8522.299896	13.88234	13.02894
65	0.45434501		6.112416429	0.415594844	0.106575872	0.032069328	0.01836944	0.304516	0.175551	0.357102	0.009088	992.4687	1000	30080.11068	4692.497265	7.531283	7.079367
														Pcostep	Pcostep$	Plifep	Pqalyp
													Per patient	171383.2172	26735.78189	40.42769	36.24901

Probabilistic Simulation Calculations

Net Benefit Calculation

Simulation Cycle	HDT+HEP+SSB-P4P Cost	HDT+HEP+SSB-P4P QALYs	Usual Practice Cost	Usual Practice QALYs	HDT+HEP Costs	HDT+HEP QALYs	HDT+HEP+SSB-P4P	Usual Practice	HDT+HEP
	152534.1	36.4526	171383.2	36.249	175257.4678	36.52413504			
1	89761.91	35.7995	101784.7	35.3638	118539.9101	34.96379958	7690932.098	7584225.774	7479527.255
2	149745.7	34.7501	149662.6	35.1029	162504.8217	34.94994669	7402880.244	7479631.821	7433551.541
3	144282.4	36.3485	180047.1	35.5682	166240.3596	36.65386945	7755747.101	7550377.303	7800148.282
4	93935.63	37.0339	123131.5	35.9978	136135.9697	35.74797802	7955050.446	7700660.31	7633365.321
5	60867.66	35.8581	65444.16	35.7883	73775.77719	35.68860248	7732573.452	7712829.421	7682820.773
6	84728.38	36.3308	109727.8	35.0142	124386.5469	34.5133387	7811433.895	7500302.896	7376776.999
7	120608.7	36.1225	158528.6	34.6012	169199.5056	34.48629076	7730293.929	7361732.011	7326085.415
8	94503.62	35.7952	110129.7	35.2295	112031.2176	35.67761367	7685252.896	7546694.382	7642177.014
9	165290.1	36.1978	202085.4	35.3504	194888.1975	36.01531917	7701981.566	7480995.369	7632717.285
10	200241.4	35.8245	266727.7	34.2297	257002.9526	34.94067898	7585888.035	7172795.312	7337039.157
11	91193.46	36.7499	122560.5	35.3092	113548.8977	36.61529867	7896076.499	7551584.798	7844456.731
12	103417.5	36.8079	135264.8	35.671	146093.7711	35.59287219	7896446.718	7617496.531	7589696.664
13	82214.96	36.1195	96190.11	35.3627	115131.0321	34.94686775	7768028.702	7589576.355	7480256.151
14	122708.7	36.6451	172633.1	34.7685	151091.0529	36.45384641	7841765.439	7383989.106	7771824.38
15	83120.1	36.5505	108245.9	35.2677	109750.9181	36.09244468	7860798.41	7556875.598	7734617.101
16	121688.5	35.8786	159091.2	34.5498	159700.8356	34.94857087	7676191.743	7350005.401	7436056.507
17	67190.8	37.0555	82771.39	35.6741	86901.79375	36.0510675	7986479.224	7670677.885	7748473.267
18	109267.7	36.6084	150954.6	34.9611	140185.2536	36.30218269	7847232.439	7447529.816	7749767.434
19	49533.87	35.582	56235.01	35.1703	66739.96723	35.45877878	7679538.434	7587711.392	7639906.471
20	121628.3	36.0596	129763.4	36.1669	146430.1383	35.73773893	7715604.467	7730782.736	7620845.778
21	82605.92	36.4213	104623.1	34.8105	106480.0468	35.81550692	7833233.512	7461116.627	7677698.042
22	120858.2	36.654	154869.7	35.4765	177717.4009	34.77428103	7845562.107	7555636.182	7380159.612
23	85242.69	36.5014	107232.5	35.3861	113972.2372	35.65327447	7848005.524	7583617.107	7634946.089
24	141131	34.486	136770.7	35.1635	170214.157	33.66990325	7354092.157	7505696.286	7147636.286
25	58449.96	37.5007	79029.31	35.4525	89486.09594	35.27981075	8091984.947	7626250.18	7578263.252
26	127679.3	36.6356	150966.3	36.1018	170249.3145	35.8732973	7834742.402	7695430.028	7626488.993
27	110082.3	35.9277	140658	34.7069	148284.4565	34.96733891	7698479.721	7402574.107	7453551.949
28	136708.2	36.2172	170784.2	35.0041	162515.4731	35.86003585	7734780.733	7437042.998	7631340.578
29	116457.2	35.6573	124312.1	35.777	140533.8679	35.37555171	7633346.546	7651489.238	7548023.915
30	103684.6	36.8076	154567.4	34.2668	149456.4182	35.25723312	7896125.435	7293010.785	7513385.885
31	67858.16	35.4779	80062.49	34.3111	88924.92963	34.41187414	7642945.747	7377137.912	7390186.208
32	109388.2	36.3137	128308.5	35.6775	144887.2036	35.27232065	7783069.916	7625880.195	7521234.24
33	82553.94	36.5765	124421.2	34.1883	100569.2188	36.82207409	7867028.909	7306107.231	7902377.186
34	84539.51	36.5074	95710.12	36.4437	134999.5774	34.04767363	7850022.34	7825003.497	7264955.856
35	73598.13	36.3737	87599.43	35.3972	107019.4075	34.47621334	7831907.903	7605653.9	7386075.275
36	119123.3	36.3403	135694.3	35.8912	176325.5279	34.37316298	7779122.562	7664925.774	7294372.087
37	92136.84	37.0653	118737.7	36.2181	136865.0125	35.72633367	7963671.477	7752944.422	7627932.075
38	102610.2	35.1554	118057.3	34.144	118218.1005	35.07890068	7538099.921	7302826.141	7505865.253
39	104875.5	36.2738	131429.8	35.0933	129460.6614	35.95868716	7776911.263	7495780.407	7685901.567
40	62066.5	36.3827	70679.54	35.9901	79263.68546	35.76221047	7845393.277	7751441.496	7693330.9
41	68983.63	36.5004	88490.07	35.2571	90351.38185	36.32491256	7864056.444	7574323.487	7804541.439
[illegible]	[illegible]	[illegible]	[illegible]	[illegible]	[illegible]	[illegible]	[illegible]	[illegible]	[illegible]

[illegible]

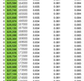

530	108788.8	35.6742	130507.6	34.6252	147584.6665	34.32078019	7644685.985	7394976.091	7311728.021	1	0	0
531	77127.92	36.9423	112895.1	34.7358	102819.2807	36.20312622	7951945.323	7436821.712	7765604.374	1	0	0
532	98530.59	36.1037	108422.1	36.0733	138709.438	34.81567831	7748293.353	7731781.731	7428164.902	1	0	0
533	107270.5	36.1689	130559.5	35.0527	134543.7326	35.66078475	7753704.834	7487824.984	7616006.885	1	0	0
534	86463.44	35.4982	106204.1	34.2067	111468.684	34.69287304	7628754.271	7328306.55	7428715.036	1	0	0
535	107228.8	36.0109	127942.8	35.3472	148338.1994	35.16687555	7719419.757	7554452.393	7494865.699	1	0	0
536	125100.3	36.2642	165139.2	34.5481	155242.2778	35.64798913	7756608.053	7343577.993	7592527.329	1	0	0
537	162834.1	35.921	199886	35.1332	204393.6671	35.4217858	7644265.782	7435997.828	7494212.68	1	0	0
538	94684.7	37.1991	115147.6	36.2565	128368.6477	36.06196945	7990198.22	7764874.71	7709375.855	1	0	0
539	123082.6	35.7022	135241.1	35.479	151745.3992	35.21630627	7636468.039	7575804.601	7502201.822	1	0	0
540	113220.9	35.0451	115681.7	35.357	128785.7115	35.01158212	7503505.331	7568854.435	7480666.557	0	1	0
541	67413.77	35.7344	85177.8	34.615	89393.71099	35.21632399	7699142.418	7438087.054	7564557.362	1	0	0
542	133593.2	35.5238	163979.2	34.2957	172364.6526	34.59305179	7587181.932	7289877.369	7346123.816	1	0	0
543	80829.26	35.6283	102369.9	34.4452	105140.0652	35.07779684	7662670.377	7383978.774	7518703.377	1	0	0
544	85817.37	35.5377	108030	33.5663	107366.0969	34.58725792	7637987.124	7187310.894	7409863.126	1	0	0
545	138161.7	35.4444	145056.3	35.8303	180472.1491	34.39666884	7565357.355	7642344.652	7295334.253	0	1	0
546	55833.21	37.0844	81609.18	34.9166	81525.25983	36.03180145	8004330.502	7507191.03	7749662.498	1	0	0
547	69184.82	36.39	84138.93	35.153	96705.26551	35.0231081	7839862.81	7559040.095	7515252.073	1	0	0
548	165388.5	36.1526	249204.4	33.6688	241758.6737	34.47269748	7692053.664	7068398.858	7250571.87	1	0	0
549	79476.07	35.7674	90479.31	35.1302	99197.76545	35.40134081	7694247.028	7544762.509	7594965.047	1	0	0
550	82509.2	35.4076	89077.3	35.3159	94889.60806	35.92805363	7613008.774	7586519.583	7713749.496	0	0	1
551	75749.55	36.5979	109501.4	34.5675	99009.26686	36.19214111	7878465.799	7403438.116	7766966.874	1	0	0
552	77583.04	36.4321	84224.39	35.872	103347.2949	35.1119159	7840602.985	7712236.747	7527911.619	1	0	0
553	70728.97	35.2755	90441.21	33.1329	83051.48082	35.27311656	7596076.841	7110687.75	7583242.945	1	0	0
554	80562.22	35.9668	115991.5	33.2511	129050.9317	33.03389641	7736490.743	7110841.85	7050569.148	1	0	0
555	105821.9	36.7058	119847.9	36.5984	135141.9798	36.30085959	7871048.803	7834487.13	7754523.144	1	0	0
556	92781.5	35.6906	102013.1	35.6261	114281.6633	35.5979928	7664239.642	7640999.298	7622621.691	1	0	0
557	80252.93	36.5362	99670.03	35.5927	109746.231	35.72393755	7860564.21	7636081.402	7654530.08	1	0	0
558	92470.58	36.358	117140.9	35.293	126302.8988	35.47522706	7809602.84	7553483.978	7583918.427	1	0	0
559	91103.5	35.5195	95419.34	35.4241	106407.9287	35.23589927	7628745.811	7603700.55	7551754.186	1	0	0
560	145842	35.6156	179268.2	34.7083	183618.7558	35.14014761	7594886.626	7364260	7454776.065	1	0	0
561	74028.93	36.71	83775.84	36.2135	107930.9545	35.06882383	7904557.937	7786898.701	7513962.286	1	0	0
562	80084.74	37.1394	92948.51	36.2342	113853.8719	35.39045248	7991832.389	7782231.134	7577942.461	1	0	0
563	138779.2	35.8315	165360.3	35.2098	167353.8852	35.64341766	7648875.284	7487166.891	7579422.152	1	0	0
564	116098.7	36.1379	147408.6	35.241	158925.2603	35.21166642	7738144.699	7511904.342	7494013.531	1	0	0
565	138522.7	35.3894	162313.6	34.5441	167963.3094	34.81313241	7553053.104	7345529.535	7398357.701	1	0	0
566	108798.2	36.2574	130733.6	35.7226	155044.8595	34.99217651	7771430.016	7633241.316	7450189.775	1	0	0
567	166351.9	34.9553	187916.9	34.6605	209278.3416	34.20061801	7430869.987	7345234.374	7223918.178	1	0	0
568	135799.9	35.1881	139450.8	35.1184	163570.517	34.52574457	7512021.711	7493211.391	7340289.333	1	0	0
569	79108.86	36.5314	99915.89	35.4153	119522.1996	34.88297756	7860665.762	7597275.115	7461979.027	1	0	0
570	108718.9	36.2656	143011.6	34.9059	150356.0896	34.99354969	7773285.17	7443465.749	7455176.994	1	0	0
571	68831.88	36.9212	90844.76	35.6425	95674.66798	36.02450003	7955661.976	7655731.096	7733926.192	1	0	0
572	138868.1	36.3076	182594	34.998	207946.922	34.21974317	7752256.535	7423916.177	7229406.277	1	0	0
573	74684.29	35.9412	76348.55	36.3895	99425.09632	35.19082207	7736806.44	7832576.785	7548983.363	0	1	0
574	61463.74	35.853	72485.34	34.757	77090.40184	35.70197665	7730864.191	7481628.727	7682412.905	1	0	0
575	107812.8	36.4941	134248.1	35.6939	138289.7502	35.91831389	7823860.128	7623508.07	7668232.509	1	0	0
576	137063	36.04	158800.6	35.4381	177415.2593	35.03229388	7695898.209	7543349.405	7436538.525	1	0	0
577	157903.6	35.5797	187878.1	34.801	181368.798	35.65958928	7575032.702	7375803.011	7568921.997	1	0	0
578	118014.7	36.4292	159670.1	34.8264	151439.2515	35.70879077	7799545.263	7409544.295	7609545.044	1	0	0
579	93779.67	36.3287	117884.3	35.3686	116419.7386	36.52353989	7801925.811	7589157.539	7821642.944	0	0	1
580	109789.5	36.0552	125442.1	35.9124	161868.2868	34.30585496	7726478.308	7679803.484	7294200.536	1	0	0
581	103152.2	35.1094	114613.8	34.9233	113675.7378	35.69073148	7527564.148	7475654.636	7643383.533	0	0	1
582	130242.2	36.189	155663.3	35.7173	179042.3123	34.98269312	7735116.135	7607174.887	7424131.193	1	0	0
583	94985.59	35.9966	133915.3	33.9361	130636.0444	34.94778939	7728560.562	7241790.025	7464951.45	1	0	0
584	96694.79	35.5539	105763.7	35.2036	111849.8486	35.52088327	7630628.786	7545417.803	7608294.442	1	0	0
585	136434.5	36.3321	165666	35.6028	187796.3609	35.14131717	7760024.544	7572290.699	7449852.654	1	0	0
586	94432.05	35.4943	112019.8	34.5379	111540.9006	35.48948065	7619933.501	7394489.348	7601778.313	1	0	0
587	148029.6	35.3387	152754.2	35.7515	176800.0407	35.05084104	7532514.614	7617521.334	7441184.802	0	1	0
588	79545.41	35.9796	95642.28	34.9151	105641.81	35.01343808	7740286.84	7492842.182	7504213.836	1	0	0
589	178170.7	35.6888	231906.4	34.3224	235181.2082	34.70527557	7578468.313	7227756.218	7307698.09	1	0	0
590	81710.41	35.9752	99210.04	34.9315	99138.83584	35.82853656	7737171.74	7492829.433	7687871.127	1	0	0
591	177731.9	36.7891	192790.6	35.057	186114.287	35.79582871	7858042.397	7426532.483	7593786.921	1	0	0
592	105305.6	36.6924	119813.8	36.0398	128857.3309	36.06826815	7869460.873	7713106.797	7710256.137	1	0	0
593	115080.1	36.149	138102.7	35.3755	153994.6211	35.10155019	7741590.195	7550448.749	7476911.399	1	0	0
594	88896.72	35.2432	95923.3	35.0828	106122.1203	34.99965365	7570903.732	7529002.442	7500737.603	1	0	0
595	112237.7	36.426	134478.5	35.5279	131241.122	35.28677943	7804624.018	7587189.999	7755363.806	1	0	0
596	76659.18	36.5811	84566.31	36.1098	90482.78458	36.2058431	7873914.081	7763572.67	7778353.405	1	0	0
597	83584.21	37.3295	128059.1	35.069	129118.3996	35.77250817	8029636.927	7493802.681	7645714.299	1	0	0
598	136199.9	35.7886	177830.7	34.0639	164135.7727	35.23536695	7642120.698	7225654.015	7493954.116	1	0	0
599	109235.8	36.12	144370.3	34.6268	137668.3483	35.48609482	7741129.444	7381460.541	7574914.985	1	0	0
600	95598.98	34.9783	100754.7	34.7622	92280.77952	36.27335195	7506615.779	7454493.305	7791405.807	0	0	1
601	56838.27	36.7482	74697.5	35.3184	73926.03705	36.40272881	7930052.179	7601448.554	7837879.444	1	0	0
602	88341.59	34.8262	112189.2	33.2549	112544	34.56334469	7480817.259	7115455.297	7399467.898	1	0	0
603	83829.17	35.0777	87230.17	35.2029	89277.13487	36.11105152	7539999.968	7563795.012	7759134.913	0	0	1
604	45504.19	36.7997	56734.5	35.6588	66560.61863	35.56666534	7952571.319	7693377.527	7663533.994	1	0	0
605	58354.2	35.902	58925.81	35.9163	78926.73432	35.03017048	7744625.587	7747148.783	7534565.547	0	1	0
606	74738.77	35.9591	78377.57	35.9341	101071.7446	34.7885179	7740657.924	7731575.446	7459899.524	1	0	0
607	55766.85	36.7818	62213.19	35.4757	78562.20995	35.40756686	7938432.699	7648120.815	7616953.778	1	0	0
608	156513.6	35.2054	188876.9	33.8895	170756.4142	35.45817046	7501057.122	7176704.288	7535757.812	0	0	1
609	78474.61	35.7754	85016.64	35.501	96204.39142	35.61068901	7696982.766	7630795.61	7643458.369	1	0	0
610	104639	35.8398	128500.9	34.7124	126648.5187	35.70615309	7684819.006	7415925.86	7633762.5	1	0	0
611	110721.7	36.4026	147125.7	34.8679	135917.3668	36.23558937	7801066.096	7431090.191	7739561.863	1	0	0
612	97357.93	36.3595	128653.3	35.0713	127058.0445	35.81734531	7805058.705	7493769.717	7657519.603	1	0	0
613	95237.67	35.6781	109769.7	35.2401	128866.0186	34.93405339	7659079.707	7549353.099	7465736.079	1	0	0
614	100570.8	36.1788	117558.3	35.5578	119692.46	36.06651664	7762556.516	7610610.812	7705999.872	1	0	0
615	123115.3	35.7852	159691.8	34.4172	164556.724	34.7218763	7654471.445	7320579.112	7381930.594	1	0	0
616	86260.81	36.5155	102323.9	36.0702	103621.3419	36.80363988	7850053.12	7737211.094	7895318.554	0	0	1
617	89419.8	36.747	123557.3	35.1069	122498.341	36.03846227	7897212.058	7506606.182	7710137.088	1	0	0
618	115453.7	36.6045	152232.2	34.9663	147688.7855	35.97785764	7840210.625	7447389.268	7671774.773	1	0	0
619	125679.6	35.7101	174070.5	33.4647	166160.416	34.65772739	7635598.185	7099186.393	7366384.713	1	0	0
620	59055.75	36.8092	66282.4	36.2459	86231.49643	35.72144124	7941102.39	7811441.605	7677502.264	1	0	0
621	79072.46	36.8491	106833	35.4083	121738.8703	35.28884563	7929748.905	7588836.578	7547974.127	1	0	0
622	96742.4	35.4835	101406.8	35.7432	118656.3162	35.20588988	7615272.145	7667061.756	7533026.996	0	1	0
623	105553.4	36.2978	131945.9	35.3904	139225.1095	35.60416206	7783455.661	7559837.747	7599019.078	1	0	0
624	68989.75	37.1995	91641.91	35.7886	96509.82593	36.40289967	8015988.345	7686684.806	7815332.79	1	0	0
625	125266.8	34.9167	124352.8	35.2718	154049.0737	33.96549119	7403569.782	7541662.335	7215004.288	0	1	0
626	140735.6	35.3111	158900.4	34.8804	154559.3116	35.82400843	7533819.343	7422038.921	7631466.504	0	0	1
627	136539.1	35.6896	143565.6	35.7757	151570.5324	35.84926673	7620269.126	7631966.35	7639944.947	0	0	1
628	49791.55	36.3046	57665.87	35.4718	63037.92131	36.16677772	7840696.887	7651813.669	7797465.714	1	0	0
629	78226.97	36.233	87921.51	35.702	109628.0492	35.01368724	7796685.572	7671577.643	7500281.75	1	0	0
630	92519.19	35.2642	93506.1	35.76	118335.6597	34.87102067	7571837.067	7678603.485	7460566.845	0	1	0
631	63648.95	36.3569	66320.1	36.4299	89945.23386	34.73266297	7838196.11	7851398.058	7458886.47	0	1	0
632	140432.1	36.0445	161280.3	35.6481	182127.8497	35.11714596	7693508.627	7580512.871	7450267.77	1	0	0
633	91314.95	36.3351	105300.2	35.6239	119808.829	35.58067742	7805792.607	7637231.328	7613331.182	1	0	0
634	93530.37	35.201	102022.5	34.9506	112068.5368	35.00436943	7557092.931	7494178.755	7495816.12	1	0	0
635	73309.23	36.3998	95280.38	35.0765	91202.9966	36.60675245	7837860.998	7528270.707	7864945.186	0	0	1
636	118267.1	35.3128	141570	34.342	136404.0495	35.20733806	7556650.292	7322351.179	7515594.012	1	0	0
637	95377.28	35.4949	115564.1	34.3081	126461.5613	34.24956753	7619128.114	7340985.016	7317373.696	1	0	0
638	104501.9	36.3196	138500.7	34.9107	161052.7706	34.10296837	7789244.697	7449929.772	7250920.478	1	0	0
639	100451.3	36.2604	122412.8	35.4755	136525.9207	35.36606111	7780422.305	7587875.518	7549969.167	1	0	0
640	130656.5	36.2317	157428	35.4764	185842.5393	34.63374983	7743972.247	7553041.761	7341491.281	1	0	0
641	86346.64	37.0049	131318.1	34.5797	118461.0887	36.08337244	7956345.121	7384266.094	7723935.161	1	0	0
642	136247.2	35.3941	173790.1	33.8624	172486.3403	34.5121917	7556335.924	7185905.917	7328427.915	1	0	0
643	78529.5	36.5103	107434.9	34.6707	108637.0648	35.57914225	7856654.424	7427940.52	7626169.291	1	0	0
644	119989.6	35.8127	154875.8	34.5764	160797.9177	34.696266	7663573.957	7359984.325	7380123.231	1	0	0
645	140281.1	35.7641	168091.2	35.0199	207420.1423	33.57680814	7632719.72	7443158.37	7090196.916	1	0	0
646	115973.3	36.5466	146664.2	35.2384	152836.1114	35.62469517	7827100.602	7512094.407	7589870.761	1	0	0
647	132326.3	35.2781	145320.2	35.3001	144960.2289	35.91022599	7535048.741	7526842.899	7659804.197	0	0	1
648	107266.6	36.3867	142939.1	35.0615	164830.9379	34.32357661	7801052.439	7477360.917	7295089.526	1	0	0
649	72996.45	36.902	89354.55	35.852	108589.7801	35.15605516	7947311.346	7702744.269	7532282.403	1	0	0
650	143020.2	35.538	166408.1	34.9908	190950.4636	33.98279896	7580839.928	7438534.675	7188905.044	1	0	0
651	118576.6	35.6626	117837	36.1036	145085.6842	35.06598754	7632363.823	7728954.605	7476191.115	0	1	0
652	151530.7	36.0123	177524.7	35.4579	181078.5506	35.68015447	7675423.45	7528926.039	7573681.902	1	0	0
653	89168.78	35.5262	109887.3	35.5591	123281.914	35.52634001	7649467.827	7618559.563	7598048.35	1	0	0
654	93198.6	36.4864	118947.7	35.2397	121824.67	35.62030545	7836791.593	7540087.28	7619928.137	1	0	0
655	132545.2	35.8944	193269.7	34.8114	200460.181	35.10136396	7886123.278	7372683.538	7428505.363	1	0	0
656	94264.21	36.1604	104321.8	35.8782	130005.3004	34.75128331	7764882.635	7693484.341	7422873.366	1	0	0
657	115938.1	36.9599	153354.8	35.9301	192832.2627	34.5614417	7916969.706	7655728.257	7318786.039	1	0	0
658	74853.37	34.7046	76130.8	35.2773	84237.78277	35.3959467	7467885.976	7591071.935	7608752.67	0	0	1
659	87117.21	35.5779	97536.72	35.3389	99709.15415	35.88084691	7645408.563	7583063.96	7698669.994	0	0	1
660	141103.7	36.2439	158511.1	36.2583	189115.8495	35.30583777	7736174.26	7721904.177	7484290.236	1	0	0
661	111236.7	36.4125	123897.8	36.3458	134145.9252	36.43104978	7802688.581	7775541.395	7783814.864	1	0	0
662	118446.6	35.6874	151072.2	34.3434	148907.4087	35.15708223	7637892.503	7313166.971	7492168.001	1	0	0
663	114295.6	36.2691	147037.2	34.7346	167507.3519	34.3029059	7768463.694	7402222.114	7287920.518	1	0	0
664	182890.4	34.4906	189418.9	34.37	213459.975	33.66307462	7313324.577	7280585.902	7102906.326	1	0	0
665	65079.65	35.9798	73968.23	35.6956	90131.08833	35.25214341	7754800.666	7684150.337	7571605.012	1	0	0
666	86039.07	36.3754	97435.85	35.9538	117603.0649	35.05292131	7819834.087	7716805.587	7500833.904	1	0	0
667	103321.8	37.2435	134424.1	36.2374	168477.6657	35.09581996	7991226.115	7741438.357	7459282.941	1	0	0
668	124964.1	35.8267	157803.5	34.7214	142074.315	36.20925105	7661637.119	7388590.573	7727680.518	0	0	1
669	70932.57	37.162	88760.34	36.241	108130.0087	35.63963491	8005884.335	7787895.552	7637823.883	1	0	0
670	108136.8	36.1418	120174.4	36.0975	139410.2816	35.62690487	7746963.22	7725287.408	7603776.85	1	0	0
671	133193.2	36.2955	161986.7	35.4041	170475.7642	35.53168851	7755297.497	7532782.663	7552016.947	1	0	0
672	81274	37.1334	117400.3	35.2771	128820.6646	35.07760524	7989330.632	7549763.964	7494981.136	1	0	0
673	87515.3	36.0804	98643.59	36.0658	118994.4593	35.36951886	7754245.148	7739922.692	7568252.139	1	0	0
674	131739.4	35.2031	159025.6	34.3011	154502.2575	35.0575639	7519344.047	7299016.796	7464943.738	1	0	0
675	183970.3	35.8073	215322.1	35.4519	206574.3461	36.05220666	7598428.819	7489838.888	7629048.302	0	0	1
676	123398.1	35.9227	146537.7	35.3194	160878.9904	35.02008184	7684078.986	7529810.275	7450420.616	1	0	0
677	133439.2	35.8957	154324.7	35.3631	152853.9972	35.98639372	7668169.18	7531535.228	7668464.801	0	0	1
678	99482.75	36.0791	114797.8	35.4885	129012.3304	35.09226335	7741979.637	7598302.164	7497975.278	1	0	0
679	87124.06	36.0275	104736.2	34.9519	99370.1958	36.19685577	7743120.186	7491745.135	7767690.634	0	0	1
680	73938.16	36.2277	98671.88	34.6622	97796.84485	35.61819817	7799832.778	7434838	7643497.965	1	0	0
681	122775.7	35.1835	128003	35.4074	124116.1671	35.40286051	7524046.542	7567476.224	7787717.938	0	0	1
682	70902.33	35.7755	81987.81	35.2217	96686.17338	34.47175384	7704589.796	7573139.874	7395439.278	1	0	0
683	101530.2	35.7825	108918.8	35.9561	124886.9237	35.52450407	7675468.123	7705824.273	7596044.314	0	1	0
684	139314.1	36.2226	171196.1	35.4569	162468.7426	36.31868557	7733338.576	7535038.867	7731070.698	1	0	0
685	93457.16	35.8993	117302.5	35.0018	115551.5	35.82815664	7708924.135	7490021.252	7671375.893	1	0	0
686	106330.1	36.3951	124207.1	35.883	134670.3848	35.84596829	7803824.171	7674639.863	7656128.21	1	0	0
687	106596.5	35.06	142302.4	33.0385	155802.851	32.81169974	7513380.05	7038324.218	6975524.781	1	0	0
688	108274.3	35.345	131729.1	34.2299	129337.8834	34.9016312	7573653.772	7307825.18	7456217.543	1	0	0
689	138409.2	35.5763	185267	33.857	176346.2671	34.95308935	7593769.646	7173241.962	7420393.125	1	0	0
690	158686.7	35.1125	167665.5	35.4741	179917.1704	35.33404192	7472702.321	7542308.216	7499618.835	0	1	0
691	153659.3	36.6369	190772	35.7745	224721.6161	35.05882543	7809048.62	7584499.405	7394998.561	1	0	0
692	105257.2	35.8332	125792.1	35.0576	143190.645	34.78595356	7682764.748	7493660.646	7417223.288	1	0	0
693	79254.52	35.9768	96769.63	34.6966	100602.9228	35.50883668	7739971.768	7444220.052	7616923.151	1	0	0
694	92491.76	35.7785	108947.3	35.1508	120594.4815	35.28804285	7683647.562	7530770.271	7548944.04	1	0	0
695	91672.34	36.7923	121836.1	35.2815	114298.4817	36.47057436	7904813.345	7546281.207	7812252.62	1	0	0
696	77279.49	36.1417	88800.66	35.4895	96940.15667	35.78065344	7777804.045	7624527.589	7679662.843	1	0	0
697	125391.7	36.1767	178177.5	33.9946	168643.1836	35.06884528	7737286.252	7210233.497	7453254.718	1	0	0
698	107178.7	35.5918	113771.9	35.742	129354.0419	35.46017814	7628374.939	7654431.2	7577596.535	0	1	0
699	109063.2	36.186	125542.9	35.6573	144274.6451	35.02115263	7755627.416	7624254.18	7467257.688	1	0	0
700	69654.94	36.4758	74112.64	36.2378	98145.30321	35.04164597	7858040.073	7801846.656	7517841.073	1	0	0
701	177595.3	36.284	215170.5	35.6088	261777.5653	34.37212822	7708412.141	7524083.146	7208695.153	1	0	0
702	71059.34	36.2943	88973.53	35.0646	98405.48622	35.06964479	7817184.171	7532000.598	7523666.182	1	0	0
703	81869.94	35.291	93466.35	34.5632	103835.9031	34.41683483	7588304.569	7418533.494	7376353.396	1	0	0
704	109431	35.3252	130983.3	34.1609	146637.4863	33.7893512	7568177.126	7293585.564	7197173.894	1	0	0
705	71196.75	36.1771	75668.89	35.8358	82429.6512	36.12317255	7791581.045	7712911.39	7768616.794	1	0	0
706	61703.57	36.3245	73984.52	35.2604	84601.53573	35.41057453	7833097.909	7589546.18	7611568.144	1	0	0
707	92989.29	36.4135	117793.8	35.2244	137313.0616	34.50069273	7821153.163	7537914.662	7361101.998	1	0	0
708	93315.92	36.4657	103044.1	36.2812	131632.6804	35.01921315	7832179.845	7782342.578	7479478.124	1	0	0
709	126076.6	34.8075	144327	33.9432	156274.6614	33.80700585	7439014.199	7232912.385	7191373.796	1	0	0
710	77180.75	35.6764	90976.98	34.8479	97720.77883	35.14293724	7676754.578	7482899.847	7540280.345	1	0	0
711	101501.5	35.1502	111398.9	34.9636	117717.8315	35.37551799	7538069.616	7487623.979	7570832.624	0	0	1
712	107368.6	36.071	116330.5	35.8345	129282.1926	35.65885338	7732342.942	7671969.186	7620848.659	1	0	0
713	94162.43	37.3866	127069.9	35.8997	146426.8478	35.33194978	8031479.539	7675400.663	7532654.449	1	0	0
714	115270.2	36.584	154567.9	34.9941	141247.6367	35.41744565	7831588.566	7451077.921	7773756.417	1	0	0
715	53527.83	35.9758	65735.33	34.2698	67717.61668	35.45656145	7765495.486	7382493.727	7638446.906	1	0	0
716	89815.53	36.8513	108384	36.1488	133028.2668	35.44776485	7919479.733	7748222.668	7571224.394	1	0	0
717	98125.72	35.4504	108764.8	35.0776	118009.7423	34.98381144	7606691.301	7515042.989	7485346.819	1	0	0
718	84053.65	36.1772	98804.41	35.2463	116542.8599	34.79815295	7778730.588	7561670.305	7446522.5	1	0	0
719	139930.6	36.2678	170935	35.6116	158231.9354	36.72933935	7742559.099	7568936.13	7824559.408	0	0	1
720	115696.5	36.3952	134089.6	36.0667	144589.5395	36.12423335	7794480.827	7704686.794	7706687.461	1	0	0
721	89230.23	35.8598	96895.57	35.8765	112974.5459	35.44058115	7704583.513	7700531.242	7589716.801	1	0	0
722	91855.99	36.8841	117249	35.9289	130585.2987	35.64189164	7924570.375	7691571.112	7615815.604	1	0	0
723	135403.5	35.1964	210376.5	31.7116	222566.9239	31.7196147	7514227.376	6681844.99	6671405.855	1	0	0
724	131479.3	35.6432	195244	32.6991	175655.3381	34.42447445	7615259.459	6911609.407	7306194.363	1	0	0
725	110778.8	36.4295	137718.9	35.2994	144071.9046	35.44268971	7806852.4	7534293.805	7559077.721	1	0	0
726	102164.1	36.4675	137079.9	35.0831	145766.7857	35.25609899	7823710.063	7487910.218	7516829.024	1	0	0
727	63467.52	35.7096	76986.66	34.747	81557.60587	35.61215299	7697693.41	7474964.38	7658423.337	1	0	0
728	128714.5	36.2518	140051.8	36.4401	168876.8441	35.66736222	7750278.28	7779875.735	7583081.594	0	1	0
729	113833.8	36.0684	159629.9	34.0395	135835.546	36.05956757	7725312.392	7238559.492	7701386.929	1	0	0
730	65105.38	36.6835	82213.11	35.4545	93759.14169	35.61092659	7907726.551	7623513.716	7647259.3	1	0	0
731	128763.7	36.4629	179230.8	34.4455	148654.5377	36.55159657	7796115.936	7307189.895	7795506.012	1	0	0
732	136105.3	36.2216	181547.1	34.6335	175408.7838	35.72045746	7736322.99	7345740.374	7588111.161	1	0	0
733	126574.3	35.9576	147608.4	34.7291	155223.0409	34.92740357	7694491.598	7400449.124	7435933.778	1	0	0
734	113808.3	35.7985	128997	35.4296	140519.6404	35.25127356	7666669.868	7571308.717	7521027.407	1	0	0
735	84011.19	36.61	117529.6	34.5915	116574.0555	35.54020119	7872836.574	7400829.685	7607768.811	1	0	0
736	109157.9	36.1591	143298.4	34.645	134033.2563	35.98634956	7749707.085	7386481.541	7687275.944	1	0	0
737	47151.84	36.8553	58017.88	36.0164	68055.35766	36.12183529	7963017.569	7769821.708	7782700.447	1	0	0
738	43411.23	37.0978	52435.46	35.4302	63307.00835	35.27457192	8019463.332	7647988.987	7603303.727	1	0	0
739	43567.39	35.9837	46034.43	35.696	62630.65612	35.09475991	7777157.333	7712168.774	7564900.158	1	0	0
740	103579.8	36.1477	112368.7	35.5943	104405.855	36.68977056	7752808.313	7623722.994	7869785.569	0	0	1
741	105291	35.7263	115363.5	35.6948	134139.5336	35.39827508	7659493.42	7642589	7559356.97	1	0	0
742	92709.43	36.8186	116900.3	35.7503	132750.3436	35.58998848	7909482.7	7653103.863	7602413.343	1	0	0
743	64758.27	36.4715	66202.42	36.5671	79730.66668	36.02764047	7881998.451	7881319.989	7750552.741	0	1	0
744	86656.71	34.8137	84719.69	35.4168	106226.8912	34.58297385	7479786.088	7612812.052	7410071.229	0	1	0
745	134756.1	34.833	148704.6	34.5458	182380.899	33.12240542	7435888.452	7359510.994	7016475.818	1	0	0
746	114350.1	35.5014	126483.7	35.4159	148249.0878	34.70485602	7601554.917	7570851.393	7394539.025	1	0	0
747	106093.9	36.3075	131379.8	35.534	136705.2639	35.72630275	7785021.825	7591618.348	7628085.102	1	0	0
748	109377.9	34.7503	118945.7	34.3519	119841.9361	35.15375693	7443279.626	7347136.898	7520510.749	0	0	1
749	107256.6	36.3019	124039	35.9662	131153.7133	36.3089159	7782627.503	7692895.859	7760262.377	1	0	0
750	126062.6	35.9714	137823.5	35.7874	162735.667	34.9492741	7692003.199	7640253.265	7433174.514	1	0	0
751	81854.9	35.3544	91234.26	34.8475	110033.1656	34.28281065	7602106.104	7482557.408	7341027.183	1	0	0
752	72133.01	37.002	88947.35	36.4348	95013.72209	36.814945	7969922.534	7829835.928	7906383.238	1	0	0
753	84295.07	36.4401	116337.7	34.2087	100804.9295	36.38703779	7835624.926	7318605.977	7807590.25	1	0	0
754	82252.68	36.4842	100301.4	35.5856	105491.7784	35.92084215	7847251.573	7633907.3	7701579.976	1	0	0
755	106483.9	35.6488	116495.2	35.5347	135471.5951	34.87136196	7641473.871	7606658.045	7443505.084	1	0	0
756	132438.5	35.1619	140883.1	34.9768	150149.9321	34.88425723	7509693.76	7461009.343	7431629.419	1	0	0
757	61558.88	35.9648	74063.36	35	84623.28912	35.08560938	7755070.305	7532865.075	7540918.138	1	0	0
758	118534.9	35.5736	119814.6	36.2072	149142.8637	35.36459649	7613066.048	7749484.648	7537033.901	0	1	0
759	134092.8	36.4287	193655	34.0489	178549.7775	35.20083826	7783354.528	7206567.746	7472035.612	1	0	0
760	101083.6	36.2154	131659.9	34.8558	136744.1373	35.3906556	7769998.083	7443944.572	7555096.342	1	0	0
761	95060.39	35.8169	118996	34.7649	116782.2922	35.60716276	7689409.796	7436839.936	7622114.068	1	0	0
762	106145.5	35.4912	123374.1	34.8194	131934.9463	34.68888922	7607541.277	7444312.592	7407382.926	1	0	0
763	153976.4	35.6032	194444.3	34.3679	232232.8601	33.06960012	7584056.662	7275105.745	6955147.099	1	0	0
764	145115.4	36.2453	183425.7	35.0981	182871.7771	35.57918938	7732475.402	7444824.971	7549944.822	1	0	0
765	155696.7	34.9387	159217.5	35.5632	177587.519	35.25983264	7437909.948	7570121.5	7485819.767	0	1	0
766	110651.2	35.7504	118985	35.9274	142710.2374	34.93891976	7659376.433	7689513.471	7450949.521	0	1	0
767	106784.1	35.5456	149742	32.9971	139049.6289	34.69966654	7618733.284	7021881.079	7402610.597	1	0	0
768	66661.72	36.0413	66029.08	36.5002	78224.01593	36.48291397	7766592.045	7866953.282	7851008.99	0	1	0
769	113257.5	35.0553	124119.3	34.4879	141499.4916	34.14983786	7505701.013	7371525.547	7280660.419	1	0	0
770	92189.03	36.5033	133386.1	33.8408	126609.3142	35.18433734	7841479.227	7221605.936	7520389.747	1	0	0
771	62243.89	36.3192	84122.28	34.4079	90321.22018	34.7570038	7831402.626	7394127.92	7463600.743	1	0	0
772	130788.7	36.0626	175278.5	34.1985	178871.2947	34.62213698	7707100.952	7257466.085	7345938.579	1	0	0
773	124546.9	35.5222	150400.7	34.6617	140053.4823	35.4204053	7595890.95	7383016.968	7552252.827	1	0	0
774	160423.2	34.6698	181244.6	34.253	174209.6276	35.13271229	7374747.957	7263328.48	7461569.195	0	0	1
775	48376.76	36.0423	55025.18	35.1799	61990.98641	35.80920419	7785084.646	7591018.735	7720817.262	1	0	0
776	41850.1	36.2673	55885.73	34.5999	64308.12848	34.70149577	7840524.204	7464093.259	7477749.664	1	0	0
777	105769.8	36.8963	154470.5	34.9192	158698.6764	35.34464023	7913304.91	7434893.957	7523140.776	1	0	0
778	94272.55	35.5882	123167.9	33.9892	134884.1472	33.79063618	7640508.517	7264080.786	7209206.512	1	0	0
779	127649.3	35.8554	146227.2	35.6176	149736.5834	36.02861853	7665202.199	7594927.48	7680759.397	0	0	1
780	132051.6	36.7467	169440.7	35.9236	185808.0623	35.74123098	7854516.458	7638219.955	7582226.82	1	0	0
781	124808.5	35.9698	144960.9	35.4979	153008.4871	35.63439535	7692900.246	7570178.633	7591806.633	1	0	0
782	93237.71	35.8579	124529.4	34.1021	122802.124	35.14654768	7700145.438	7287251.274	7515983.695	1	0	0
783	106786.3	35.0846	141674	32.9289	134367.3386	33.96831841	7518526.865	7015133.343	7248340.954	1	0	0
784	47277.57	36.231	57319.78	34.6552	62021.88563	35.39341696	7827208.394	7474668.03	7630418.749	1	0	0
785	113901.8	36.3299	133453.7	35.9012	147450.6103	35.86911007	7782082.067	7669345.646	7648377.641	1	0	0
786	99641.2	35.935	118483.1	35.0784	134468.0885	35.00376076	7710500.873	7505468.723	7473284.279	1	0	0
787	115266.3	36.1499	143460.9	35.1007	146681.0436	35.57075888	7741597.127	7485364.948	7584303.262	1	0	0
788	65103.67	36.6204	72609.77	36.3107	92510.35753	35.19222874	7894000.457	7819184.623	7556203.829	1	0	0
789	215496.6	35.0587	265932	33.9479	265295.9806	34.31872515	7404188.721	7112335.186	7193570.062	1	0	0
790	74364.9	35.5328	104048.9	34.296	108326.6626	34.88663264	7865702.96	7349883.165	7475968.962	1	0	0
791	98144.13	36.1385	129168.9	34.375	129420.5111	35.00679179	7756229.65	7341929.115	7478990.624	1	0	0
792	84028.42	36.5718	106305.5	35.3705	106730.2594	35.90240094	7864533.105	7581164.786	7696333.464	1	0	0
793	84150.2	35.7633	117987.5	33.4191	112538.2329	35.12663845	7688673.447	7145357.945	7521920.494	1	0	0
794	96459.95	35.6263	106159.2	35.5192	132040.9863	34.27689532	7646606.023	7613616.771	7317690.252	1	0	0
795	134910	35.2349	159993.1	34.2486	171173.7404	34.17600386	7523078.585	7283630.642	7256673.114	1	0	0
796	81223.55	36.4329	100509.1	35.1867	100041.7834	35.61231926	7837144.492	7547010.76	7633975.298	1	0	0
797	75359.94	36.8668	94109.74	35.5636	99432.22496	35.93306849	7937314.354	7635326.352	7710296.813	1	0	0
798	119985.1	34.83	127123.9	34.6409	128409.8783	35.25310559	7450006.716	7401766.889	7533535.344	0	0	1
799	113969	35.5375	136998.2	34.882	142416.0654	35.10004145	7609781.215	7444291.226	7486262.044	1	0	0
800	70201.35	36.0885	76635.55	35.8095	98904.63025	35.30964035	7773304.84	7706233.173	7575327.912	1	0	0
801	73134.91	36.4516	85173.17	35.7781	98382.84292	35.55344501	7849282.942	7690869.096	7628838.449	1	0	0
802	64621.73	36.2228	72751.67	35.5977	78323.0408	36.11311117	7808077.47	7664087.062	7770536.654	1	0	0
803	152508.2	35.573	176941.9	35.1167	192333.7807	34.90935508	7578955.594	7455361.909	7394900.361	1	0	0
804	64100.4	35.5013	73246.58	34.9022	92233.27093	34.08369962	7651778.403	7512442.591	7315552.089	1	0	0
805	186826.2	37.028	280548.9	34.937	271175.1236	35.56544298	7860872.711	7312690.863	7458653.82	1	0	0
806	101956.1	36.4254	144902.9	34.1517	149046.9059	34.80967714	7814779.388	7277658.805	7416523.134	1	0	0
807	65401.79	36.2181	84469.76	34.455	82745.70891	35.71405701	7806284.069	7404008.927	7679383.155	1	0	0
808	127544.1	34.9766	162178.8	33.5178	138067.7982	35.79551254	7474312.444	7122621.324	7641764.694	0	0	1
809	114780.7	35.9807	142215.8	34.6883	145490.0648	35.07513575	7705295.919	7396966.976	7477775.014	1	0	0
810	79386.28	36.6966	113546.5	34.4627	99977.32691	36.53632922	7896279.319	7376611.595	7840865.003	1	0	0
811	148443.5	36.5657	168392.8	36.4683	185203.8238	36.16066091	7798772.071	7757664.88	7673990.38	1	0	0
812	157473.8	35.4301	190179.1	34.4474	194055.9159	34.88197137	7542936.067	7296650.064	7387226.623	1	0	0
813	73192.1	36.9326	89091.31	36.216	101580.6729	35.86291339	7953767.485	7782130.09	7692900.786	1	0	0
814	72913.41	36.2691	97585.46	34.6264	111624.4885	34.48045295	7809844.563	7428160.595	7382391.636	1	0	0
815	80508.68	36.5947	101414.5	35.3357	112377.8953	35.55937456	7873012.789	7578489.067	7616132.13	1	0	0
816	58715.85	37.4966	73780.08	36.3446	81974.70392	36.40561693	8090824.022	7825381.609	7830458.485	1	0	0
817	212642.3	35.2185	266218.7	34.0486	253875.3632	34.81327834	7441774.92	7133943.205	7312477.365	1	0	0
818	108343.9	36.3352	151483.4	34.371	153023.8599	34.88483587	7788786.969	7318751.969	7428881.253	1	0	0
819	76851.27	35.5517	95908.88	34.1576	102275.6316	34.77489887	7650000.281	7327927.331	7455735.664	1	0	0
820	94466.77	35.5199	120011.2	33.8195	125239.4961	34.03995954	7625454.414	7230353.819	7273039.35	1	0	0
821	115458.4	36.1902	150687.3	34.7996	162938.5497	34.87184589	7750154.739	7412696.351	7416143.308	1	0	0
822	124478.9	35.9122	138184.9	35.6747	153299.5821	35.22276058	7680717.541	7615392.036	7502050.425	1	0	0
823	102795.9	35.6197	123124	34.3772	127097.8402	34.62745424	7638829.992	7348452.154	7398867.692	1	0	0
824	136531.5	35.7809	167044.3	34.9117	174357.0622	35.02935821	7640116.723	7420690.101	7438958.68	1	0	0
825	53709.09	36.4868	66668.85	35.2433	88043.09864	33.94679657	7876368.923	7593134.62	7289987.615	1	0	0
826	127656.3	35.8604	141024.5	35.8694	152211.3861	35.81917099	7666285.244	7654866.391	7632763.057	1	0	0
827	82682.65	36.0763	76042.49	36.6719	99176.61479	35.69587294	7758184.543	7894274.395	7659000.106	0	1	0
828	116806.5	36.2384	165747.3	34.2426	165867.2016	34.82274507	7759292.533	7276567.719	7402543.034	1	0	0
829	81818.12	37.1023	104104	36.3068	119665.577	36.00992786	7982032.471	7786847.884	7706768.155	1	0	0
830	137317.5	35.2417	163710.8	34.4939	170524.637	34.53994363	7522156.029	7333220.489	7336421.252	1	0	0
831	93799.69	35.7391	101879.4	35.5172	125677.7897	34.709178	7673770.46	7617459.784	7418049.667	1	0	0
832	92298.5	36.1692	116628.6	34.6179	112515.6664	35.49185585	7766804.89	7407262.918	7601319.776	1	0	0
833	124178.1	35.9161	182373.9	33.3493	167967.6368	34.84370476	7681873.458	7065801.315	7404998	1	0	0
834	90492.17	36.7212	107135.7	35.8674	119372.9807	35.53650174	7890523.508	7688316.69	7604165.844	1	0	0
835	86083.03	35.8608	134219.9	32.37	124296.28	34.11375472	7707934.537	6901110.912	7290021.284	1	0	0
836	68906.41	35.0863	81158.85	34.2325	92017.55959	34.03924969	7556785.157	7358976.233	7306107.008	1	0	0
837	108785.8	36.4161	146378	34.8676	135934.5896	36.17730776	7805934.498	7431783.234	7726877.657	1	0	0
838	67411.52	37.1877	93661.15	35.2119	90854.94819	36.47630562	8014991.001	7559326.917	7836941.792	1	0	0
839	114157.6	35.1432	129691.1	34.5002	138931.2693	34.55403549	7523905.501	7368609.54	7371077.359	1	0	0
840	76355.92	36.1843	97275.59	34.6377	110958.0292	34.31439234	7787969.075	7430911.09	7346966.317	1	0	0
841	90264.4	35.1814	93453.53	35.3793	118398.397	34.03401439	7556086.428	7595926.562	7278588.324	0	1	0
842	121485.8	35.282	135433.5	35.1067	163488.633	33.93010442	7546732.83	7494682.434	7210914.192	1	0	0
843	104404.4	36.2573	121920.1	35.7573	132341.7599	35.78098132	7775795.304	7649611.766	7644332.5	1	0	0
844	122156.3	36.4521	155098.4	35.3607	157467.7869	35.97992223	7800381.294	7530228.72	7662444.492	1	0	0
845	123582.8	35.5142	148585.3	34.7412	148581.3722	35.42518918	7595124.345	7402105.181	7550764.669	1	0	0
846	105586	35.653	110512.3	35.7642	135892.799	34.92513941	7643263.732	7662509.601	7454771.925	0	1	0
847	83437.39	35.9937	113574.7	34.102	114326.2145	35.07607873	7739467.606	7298179.966	7509143.813	1	0	0
848	115737.2	36.1451	140773.7	35.2578	133823.0866	36.32444103	7740077.635	7522188.135	7760967.252	0	0	1
849	83136.74	36.4375	107676.5	34.9727	109640.7767	35.53876874	7836215.786	7493335.873	7614390.76	1	0	0
850	99492.98	35.3676	114841.1	34.7284	129013.5597	34.3390798	7587327.086	7433071.543	7334276.383	1	0	0
851	73577.45	36.2755	85486.13	35.9704	93512.88828	36.1002023	7810568.989	7732348.126	7752541.18	1	0	0
852	126816.3	35.4561	128976.2	36.0499	160789.4437	35.0206079	7579244.299	7706146.258	7450624.499	0	1	0
853	81740.51	37.1875	115604.1	35.4768	117924.719	36.17097484	8000626.99	7594965.318	7743511.123	1	0	0
854	55187.77	36.6922	64773.92	35.8511	79677.56405	35.55011159	7919525.378	7727146.665	7646519.24	1	0	0
855	89289.4	36.1257	101012.5	35.6581	114998.3905	35.32758586	7762312.963	7648948.075	7563134.448	1	0	0
856	88644.41	35.9374	107535.9	34.7096	107692.4291	35.72727099	7722017.301	7436287.6	7657308.375	1	0	0
857	135144	36.2152	177360.3	34.8608	170988.357	35.65452765	7735904.609	7399314.526	7578202.337	1	0	0
858	69851.16	36.7053	94256.25	34.9074	103606.8918	34.7350909	7907715.009	7492558.794	7446752.499	1	0	0
859	88578.02	36.0013	108692.8	35.0423	125845.7515	34.90648507	7735983.054	7507425.527	7460764.619	1	0	0
860	128945.6	35.5877	154902.8	34.5707	145909.3369	35.57781039	7605723.837	7358718.871	7586607.55	1	0	0
861	109779.8	35.4285	120401.5	35.3985	131832.1986	35.29329671	7590295.868	7573152.18	7538848.201	1	0	0
862	80059.19	36.9727	126568.1	34.029	138754.0165	33.89208569	7955630.543	7269335.033	7227385.78	1	0	0
863	77473.22	35.9267	81037.52	35.898	89730.48952	36.106647	7730875.297	7721080.167	7757724.276	0	0	1
864	119265.7	35.9811	135942.8	35.5529	137140.0624	36.18413798	7700894.338	7591150.707	7727156.67	0	0	1
865	116295.5	35.2731	158964.8	32.8431	164003.2376	33.12981758	7549988.696	6979176.854	7036464.445	1	0	0
866	68693.33	35.847	83204.37	34.4773	76424.4614	36.36000742	7722328.349	7410116.61	7826095.912	0	0	1
867	71893.48	36.1548	85976.58	35.5632	100449.6254	35.06415589	7786021.377	7643356.168	7520429.08	1	0	0
868	68100.14	36.3359	66352.01	36.7949	95169.40142	34.87571899	7829183.773	7930695.426	7484754.239	0	1	0
869	116660.3	35.9705	164497.1	34.0463	150413.8937	35.55082925	7701205.861	7235153.996	7576238.887	1	0	0
870	85018.52	35.6933	76074.55	36.6357	92184.40516	36.16249884	7672605.633	7886371.152	7767409.256	0	1	0
871	144857.4	35.9287	148367.9	36.4907	168862.6793	36.07523221	7663913.771	7782564.442	7671764.365	0	1	0
872	101239	36.492	128921.5	35.4019	126326.1172	36.43173941	7829979.117	7565362.853	7791784.559	1	0	0
873	70536.71	36.1786	81995.24	34.8661	79427.67534	36.10645891	7792556.389	7495840.746	7767986.21	1	0	0
874	90428.49	37.1852	127065.4	35.3616	123637.6864	36.12906111	7991438.854	7558458.692	7728688.585	1	0	0
875	111771.6	35.7805	143644.7	34.362	147540.6998	34.53027435	7664790.534	7324637.621	7357303.658	1	0	0
876	97488.22	35.7708	126747.4	33.7257	132671.8538	34.06438935	7676962.451	7203235.616	7270916.592	1	0	0
877	80776.18	36.2426	95089.12	35.5171	108145.3903	35.24883066	7796223.304	7624228.556	7552870.713	1	0	0
878	110008.3	35.6446	113881.4	36.0296	138147.4542	35.28985992	7637021.195	7716818.713	7531785.991	0	1	0
879	50961.78	36.8312	58294.55	36.1401	71241.26248	35.75810003	7953972.564	7796437.951	7700459.957	1	0	0
880	101066.1	36.547	122019.6	35.7543	134343.8642	35.70432168	7842096.135	7648860.915	7625669.113	1	0	0
881	65225.63	36.4559	80669.88	35.1967	76941.59816	36.75610152	7858142.362	7569023.435	7911666.262	0	0	1
882	83651.77	35.1198	110014.9	33.1013	98099.60293	35.18627588	7549310.791	7084249.554	7549320.782	0	0	1
883	90627.58	35.8597	123687.1	33.6622	110874.5271	35.70866054	7703158.88	7192486.521	7650081.464	1	0	0
884	100788.8	35.6292	136720.4	33.1108	130558.8142	34.24863786	7642906.466	7059623.665	7313074.387	1	0	0
885	83642.9	35.507	100052.8	34.3228	105426.9656	34.70257555	7633490.634	7359705.167	7436865.507	1	0	0
886	87229.14	35.9694	99238.31	35.5581	110677.5381	35.53380033	7730393.886	7629000.284	7612287.2	1	0	0
887	103129	36.1267	122311.7	35.2983	131028.5513	35.41070731	7748682.71	7549461.027	7565169.986	1	0	0
888	92218.14	36.143	101842.5	36.0339	101023.8939	36.72033163	7763140.97	7729796.98	7879809.702	0	0	1
889	89304.7	35.7972	113249.9	34.3651	116797.2463	34.99508866	7690884.126	7355687.539	7489070.318	1	0	0
890	107534.3	36.5698	136308.1	35.6496	156822.0475	35.30502551	7840571.883	7611814.974	7516407.502	1	0	0
891	86333.46	36.3698	106692.9	35.4537	107707.9985	36.29031354	7818322.175	7598859.218	7779665.037	1	0	0
892	144509.9	36.1822	166867.7	35.6873	173856.7154	35.87502217	7719371.817	7589446.579	7623256.478	1	0	0
893	121109.2	35.0884	172355.3	32.1192	156667.6317	33.85496172	7505043.854	6808467.238	7201403.604	1	0	0
894	106162	35.5584	146381.1	33.301	134478.2503	34.88461618	7622138.453	7091284.633	7447379.114	1	0	0
895	73848.25	35.6759	74428.28	35.9118	91525.75971	35.51806236	7679989.269	7730671.918	7628005.432	0	1	0
896	104881.4	35.9361	120184.1	35.4422	148776.4981	34.19348113	7705514.945	7582853.404	7282868.884	1	0	0
897	119820.1	36.0357	119947.4	36.6265	166169.0941	34.5435953	7712217.713	7840489.096	7341570.452	0	1	0
898	96616.69	35.0231	112270.4	34.2681	120561.5588	34.4145242	7515339.762	7335590.808	7359125.545	1	0	0
899	67789.01	36.5842	71491.21	36.5759	84903.03185	36.33107622	7883458.333	7877952.061	7811329.405	1	0	0
900	120565.2	35.9647	141248	35.121	160698.5259	34.63017827	7696028.114	7491987.151	7365859.05	1	0	0
901	104787.3	36.3459	119443.7	35.7696	144087.2455	34.84959765	7794669.67	7654757.943	7430159.157	1	0	0
902	127849	36.3498	186264.6	33.9668	188689.5419	34.38342136	7772462.779	7196120.763	7284237.64	1	0	0
903	85793.52	36.5653	86963.82	36.6892	111815.5318	35.85580199	7861347.255	7887108.79	7681120.329	0	1	0
904	87460.49	36.5205	122893.4	34.5242	123597.7216	34.98606414	7849935.135	7380629.306	7480308.445	1	0	0
905	50359.37	36.8713	65288	35.0419	75614.29191	35.18234873	7963275.248	7550752.582	7570952.562	1	0	0
906	125348	36.3532	139805.4	35.7682	153404.9562	35.74196136	7775698.177	7634086.752	7614788.668	1	0	0
907	68200.86	36.6217	94048.55	34.3807	102585.2934	34.63622625	7891193.084	7378289.376	7425286.756	1	0	0
908	109351.7	36.0329	140225.8	34.6753	145073.1908	35.02383454	7722068.661	7396139.955	7467042.032	1	0	0
909	63351.06	36.5185	70732.19	36.0543	79550.56081	36.58462771	7873610.277	7765343.174	7871789.011	1	0	0
910	147866.8	34.4167	168047	33.83	162752.4249	34.70798039	7332291.746	7184606.208	7380714.742	0	0	1
911	109403.9	35.4801	129346.3	34.637	151169.1529	33.8639966	7601874.067	7398695.805	7208865.731	1	0	0
912	119288.6	36.1822	159739.3	34.7165	162974.3939	35.24519345	7744585.552	7385587.613	7497251.196	1	0	0
913	77966.25	35.2261	91348.53	34.3712	109469.0339	33.81006983	7578103.918	7378920.929	7238845.353	1	0	0
914	131904.8	34.2176	159711.7	32.9768	159316.0185	33.76271953	7304975.812	7007504.483	7178707.207	1	0	0
915	98723.07	35.7849	148933.1	32.5737	127275.3467	34.97448197	7678804.355	6930658.135	7474113.54	1	0	0
916	61055.5	36.8101	73879.18	35.978	86977.83259	35.76587111	7939284.893	7745615.753	7686412.361	1	0	0
917	172364.5	35.5722	228737.9	33.5375	190895.818	35.5380573	7558923.34	7060335.939	7532981.093	1	0	0
918	92093.24	36.1037	107075.2	35.4554	108917.3562	36.11293929	7754713.22	7598846.77	7739904.983	1	0	0
919	100053.3	35.3726	116122.6	34.714	127935.5249	34.52103982	7587854.386	7428662.362	7374901.791	1	0	0
920	83350.24	35.8744	104926.4	34.4925	118121.7864	34.05601946	7713636.286	7391698.85	7283647.54	1	0	0
921	79597.58	36.3829	103406.1	34.598	102000.8315	35.28170132	7827905.338	7416163.314	7566159.414	1	0	0
922	71835.61	35.9267	90897.79	34.5339	99469.41056	34.49540163	7736517.158	7414733.656	7397795.675	1	0	0
923	123212.1	35.9624	170826.1	34.0542	166882.5165	35.04071347	7692895.441	7230539.286	7448901.19	1	0	0
924	81479.67	35.5701	94839.08	34.763	107308.1339	34.93753691	7649356.353	7460589.51	7486051.075	1	0	0
925	122093.4	34.4743	123188.2	35.0153	147359.2679	34.14525197	7370588.108	7487061.638	7273803.94	0	1	0
926	93474.86	35.376	103365.8	35.1953	104284.2135	35.68842194	7595176.573	7546026.222	7652273.1	0	0	1
927	152154.9	36.3323	166774.4	36.3657	188989.3575	35.87706951	7744339.753	7736985.788	7608568.807	1	0	0
928	77033.27	36.5027	110408.1	34.3439	118930.9403	34.70160798	7856492.899	7353938.961	7423151.241	1	0	0
929	50174.34	36.3973	62940.23	35.3912	65103.8554	36.05953821	7860453.386	7629024.025	7772112.239	1	0	0
930	97815.81	36.2877	136505.8	34.3381	125246.0723	35.82664252	7788989.092	7326567.303	7661352.24	1	0	0
931	130034.3	35.4283	164637	34.0234	183493.5609	33.56625203	7569979.021	7230047.529	7111829.222	1	0	0
932	109941.9	36.0268	127881.4	35.5605	140002.968	35.47613111	7720167.452	7600867.201	7570414.845	1	0	0
933	36859.06	36.3869	46474.02	34.8155	55660.17746	35.33579489	7871509.683	7520365.412	7624256.819	1	0	0
934	99745.07	36.24	134117.5	34.5305	126185.7721	35.71957622	7776682.955	7370777.645	7637142.643	1	0	0
935	178463.8	35.4913	223360.8	34.2629	211574.4584	35.23823987	7535255.441	7223382.22	7447139.832	1	0	0
936	100410.1	35.9632	126063.9	34.7505	125709.435	35.42676517	7715858.731	7426651.836	7573979.135	1	0	0
937	95757.5	36.7365	138079	34.605	133269.971	35.79909207	7888585.184	7383007.596	7647340.499	1	0	0
938	82168.53	36.8618	114819.3	35.026	118771.3527	35.39210576	7929412.941	7497775.86	7573384.306	1	0	0
939	57525.43	35.5095	63418.3	35.185	66816.75796	35.82610229	7660151.877	7583732.11	7719664.139	0	0	1
940	46348.76	36.6511	61887.68	35.1763	62890.05625	36.18343263	7919447.521	7583354.059	7801253.375	1	0	0
941	111433.2	36.4314	134065.1	35.5882	138467.9249	36.15343292	7806603.56	7600712.593	7719155.339	1	0	0
942	57037.85	36.1787	63995.45	35.3733	76292.91333	35.62279	7806087.16	7624077.875	7665999.888	1	0	0
943	85500.91	36.5623	136476.7	33.4776	123418.4797	35.07150027	7860981.968	7139574.277	7499069.5	1	0	0
944	99737.01	36.2093	122186.5	34.7766	137970.7343	34.57235898	7770030.276	7436201.832	7376020.339	1	0	0
945	78492.4	36.7344	98654.57	35.4275	100449.236	36.56620716	7905390.652	7601190.542	7846886.794	1	0	0
946	97446.9	37.2145	121624.9	36.5224	133297.3095	36.34174335	7990787.034	7816183.81	7765253.532	1	0	0
947	68056.71	36.952	74952.37	36.5567	82141.66324	36.82052763	7963130.2	7870327.913	7920468.631	1	0	0
948	94335.28	36.158	118638.7	34.8917	101163.0753	36.84348877	7764288.287	7464751.377	7906437.617	0	0	1
949	130170.7	36.1013	145722.6	35.8559	163852.1069	35.5373173	7716127.887	7647226.979	7559863.973	1	0	0
950	113055.4	36.4785	150977.6	34.9866	150934.3377	35.49471419	7815211.344	7453054.854	7563522.339	1	0	0
951	107833.3	36.1582	131753.2	35.0926	131577.9418	35.80945366	7750830.75	7495304.087	7651284.525	1	0	0
952	128519.4	35.9135	173796.2	34.165	178391.4192	34.51981221	7676952.397	7251653.299	7324179.085	1	0	0
953	98371.87	36.1046	116600.3	35.1424	113909.3123	36.03086563	7750627.185	7521283.509	7717075.055	1	0	0
954	79231.56	36.4211	109342.3	34.5899	107291.0475	35.48220226	7836571.692	7408458.154	7604446.273	1	0	0
955	82130.46	36.2394	87506.89	36.1401	113846.213	34.90463066	7794177.165	7767223.226	7472361.12	1	0	0
956	162270.6	36.4541	181965.5	36.425	221761.0531	35.23807027	7760705.188	7734673.332	7436916.377	1	0	0
957	136293.8	36.97	174678.3	35.967	191948.8344	35.84811263	7898807.84	7642426.134	7599315.813	1	0	0
958	131270.1	35.6378	164542.1	34.3375	147020.7492	35.78242977	7614293.71	7298394.04	7629968.32	0	0	1
959	119502.5	35.9425	132135	35.7692	134079.4702	36.2475064	7692274.967	7641978.429	7743989.818	0	0	1
960	109855.2	34.6948	134622.2	33.5798	133742.652	34.35498387	7430751.507	7163642.816	7333003.897	1	0	0
961	98715.33	36.3137	106430.5	36.4788	125333.8149	36.08581121	7793745.595	7821912.346	7717592.479	0	1	0
962	107800.2	36.1911	152486	34.2643	158166.0124	34.5464387	7758002.534	7294540.783	7350191.52	1	0	0
963	73554.51	36.4511	86250.3	35.5987	93795.87011	35.8077456	7848774.759	7650811.464	7688695.366	1	0	0
964	89109.97	35.9245	92722.23	36.1383	100522.0828	36.25979029	7718755.509	7761615.517	7780216.998	0	0	1
965	139146.2	35.86	160356.9	35.4785	183178.8503	34.8152413	7654706.024	7550575.429	7383600.51	1	0	0
966	113870.3	35.6858	124682.1	35.1933	149384.8958	34.15959122	7642108.428	7524258.239	7274894.82	1	0	0
967	150524.7	35.3926	189484.4	33.9941	179636.8722	35.04597321	7541732.49	7198824.027	7437289.991	1	0	0
968	147502.2	36.4587	205953	34.3513	217915.0648	34.29483306	7776470.743	7260001.045	7235758.247	1	0	0
969	73943.1	35.9697	85612.22	35.0728	91239.41985	35.74002992	7743738.101	7537141.302	7676534.423	1	0	0
970	102658.1	35.5293	111790.2	35.4155	134720.0324	34.46421857	7619315	7585445.83	7355767.696	1	0	0
971	143433.5	36.2929	176907.3	35.4916	210432.5062	34.56534001	7744495.829	7536864.645	7302011.323	1	0	0
972	91813.08	36.1048	114847.7	34.9166	112806.7638	35.91356787	7755246.085	7473966.382	7692683.992	1	0	0
973	87396.08	36.1061	106195.9	34.8648	123626.1356	34.11123436	7759946.703	7471363.131	7290143.651	1	0	0
974	134085	35.0291	156511.9	34.3431	170085.399	34.36720189	7479169.774	7307656.661	7299316.627	1	0	0
975	81293.92	36.3231	103596.2	35.2428	114408.971	35.28155018	7813194.568	7556109.248	7553718.427	1	0	0
976	165124.4	36.5129	231068.7	34.7689	236587.1922	35.16148824	7770624.573	7325644.955	7405445.823	1	0	0
977	125102.5	36.7804	187634.4	34.4576	167361.294	35.96015787	7868780.379	7301420.761	7648255.378	1	0	0
978	56701.69	36.5118	62291.96	36.1134	70579.84522	36.42619673	7878808.062	7786621.534	7846326.178	1	0	0
979	102729.2	35.8172	133236.5	34.0308	135165.1019	34.65285494	7681822.696	7263053.831	7396321.045	1	0	0
980	121258.3	35.7102	146924.1	34.9836	169280.4563	34.18611618	7640025.455	7456447.933	7260764.221	1	0	0
981	67742.16	36.2783	74635.88	35.8405	79556.64128	36.54166526	7817027.958	7714971.651	7862445.428	0	0	1
982	68029.02	36.7707	78722.61	36.3896	100177.0801	35.62976231	7923745.821	7830238.017	7643631.09	1	0	0
983	105564.7	35.729	154578.2	33.0687	145222.6808	34.29018907	7659817.987	7032604.415	7307441.301	1	0	0
984	106227.3	34.8656	103073.2	35.4373	126908.4396	34.54955006	7471502.347	7598905.425	7382125.319	0	1	0
985	135831.2	36.2763	173597.3	34.8905	147480.8878	36.57849783	7748486.223	7409529.559	7802526.409	0	0	1
986	82574.92	36.8994	100387.5	36.1755	113328.4025	35.90017109	7937168.353	7782024.574	7689250.682	1	0	0
987	102108.7	36.5715	116738.5	36.2693	128338.1878	36.13187503	7846376.008	7766065.136	7724599.663	1	0	0
988	128434.1	35.1861	157087.2	34.22	125801.1075	35.59399166	7518939.513	7280320.519	7627573.634	0	0	1
989	118052.5	34.6744	112941.4	35.5635	131100.4158	34.90843205	7418125.294	7616462.085	7455933.115	0	1	0
990	83540.77	35.8647	91205.59	35.4544	99695.54074	35.47741003	7711326.804	7614498.842	7611000.233	1	0	0
991	88634.77	35.9078	94918.19	35.9743	96414.20556	36.71085492	7715611.312	7723768.484	7882359.713	0	0	1
992	80241.6	35.9377	93363.92	35.2343	109794.1379	34.70132011	7730503.876	7564499.489	7432225.476	1	0	0
993	106336.1	36.3123	126644	35.5438	129525.7801	35.98440157	7785812.762	7598470.506	7691360.042	1	0	0
994	78681.99	36.8187	85596.39	36.4308	102859.506	35.97312424	7925538.956	7832302.986	7715575.289	1	0	0
995	90970.21	34.9625	94914.39	34.9204	118401.9894	33.65741334	7507819.671	7494725.502	7196733.884	1	0	0
996	101315.5	36.4012	128400.5	35.3527	129012.1089	36.02853811	7810168.128	7555191.596	7701466.393	1	0	0
997	143928.3	35.1819	158187	35.1096	180722.4931	34.54660928	7502550.325	7472565.166	7327672.114	1	0	0
998	102642.9	35.6002	125391.5	34.5035	137395.3474	34.49630888	7634744.251	7373639.294	7360066.921	1	0	0
999	65200.52	36.5513	74252.63	36.0527	81130.65185	36.53840898	7878885.441	7761470.826	7860163.695	1	0	0
1000	151331.8	36.6634	166063.8	36.7219	199745.0143	35.73332039	7817120.665	7815104.651	7566570.574	1	0	0
	**Dcostps3**	**Dqalype3**	**Pcostps3**	**Pqalype3**	**Icps3**	**Ieps3**	**NMB3**	**NHB3**				
	101079.6	36.0624	122908.8	35.1382	-21829.14809	0.924240173	7736759.651	7514055.22				

Deterministic Sensitivity analysis		3.0000	4.0000	5.0000	6.0000	7	8	9	10.000	11.000	12.000	13.000	14.000	15.000
		model value	base value	lower	upper				Low(=2)			High(=3)		
	Discounting rate	0.0500	0.0500	0.0000	0.0800	1			-26512.027	-34535.129	29649.690	-21063.916	-27297.888	22573.887
序列	SSCR	0.6200	0.6200	0.4960	0.7440	1			-23503.929	-27678.433	-30607.909	-23503.929	-21816.891	-22824.078
1	DSCR	0.6900	0.6900	0.5520	0.8280	1			-30399.647	-22760.911	21543.760	-25338.570	-27592.528	21543.760
2	PSCR	0.6100	0.6100	0.4880	0.7320	1			-25254.327	-29939.313	-22769.865	-31922.511	-29939.313	-30683.012
3	P0.5	0.3234	0.3234	0.2910	0.3557	1			-21584.78871	-27697.56727	21204.66114	-25636.92281	-32430.86125	21920.64623
	P1	0.5303	0.5303	0.4773	0.5833	1			-20263.36032	-26191.34552	21232.53613	-27351.1702	-34388.9444	21913.249
	P2	0.1205	0.1205	0.1084	0.1325	1			-22738.28048	-29046.93766	21422.31975	-24300.9681	-30868.2750	21670.180
	P3	0.0259	0.0259	0.0233	0.0285	1			-23355.18472	-29762.16028	21493.64421	-23653.9979	-30118.0437	21594.323
	p0.5_1	0.0413	0.0413	0.0330	0.0496	1			-23248.51008	-29689.79626	21840.49314	-23744.18931	-30173.99408	21264.44404
	p0.5_2	0.0056	0.0056	0.0045	0.0067	1			-23483.940	-29917.270	21549.370	-23523.630	-29961.038	21538.224
	p0.5_3	0.0016	0.0016	0.0013	0.0019	1			-23513.368	-29947.697	21526.935	-23494.528	-29930.963	21560.514
	p1_0.5	0.1675	0.1675	0.1340	0.2010	1			-24014.203	-30436.665	20943.028	-23093.741	-29539.654	22027.651
	p1_2	0.0072	0.0072	0.0058	0.0086	1			-23511.839	-29945.541	21524.071	-23496.098	-29933.148	21563.256
	p1_3	0.0016	0.0016	0.0013	0.0019	1			-23512.933	-29947.691	21530.375	-23494.947	-29930.956	21557.113
	p2_0.5	0.0227	0.0227	0.0182	0.0272	1			-23519.746	-29956.741	21539.219	-23489.082	-29922.957	21548.045
	p2_1	0.0289	0.0289	0.0231	0.0347	1			-23490.011	-29928.290	21577.943	-23516.736	-29949.446	21512.239
	p2_3	0.0008	0.0008	0.0006	0.0010	1			-23505.401	-29940.736	21541.950	-23502.460	-29937.893	21545.567
	p3_0.5	0.0060	0.0060	0.0048	0.0072	1			-23501.663	-29937.304	21547.828	-23506.152	-29941.284	21539.772
	p3_1	0.0060	0.0060	0.0048	0.0072	1			-23499.266	-29934.961	21550.600	-23508.493	-29943.573	21537.063
	p3_2	0.0008	0.0008	0.0006	0.0010	1			-23503.442	-29938.841	21544.349	-23504.414	-29939.783	21543.173
	p0.5_CVD	0.0014	0.0014	0.0012	0.0017	1			-23386.355	-29821.108	21656.913	-23620.916	-30056.928	21431.173
	p1_CVD	0.0022	0.0022	0.0018	0.0027	1			-23425.729	-29860.494	21617.628	-23581.810	-30017.810	21470.195
	p2_CVD	0.0035	0.0035	0.0028	0.0042	1			-23452.107	-29887.437	21595.202	-23555.229	-29990.667	21492.838
	p3_CVD	0.0035	0.0035	0.0028	0.0042	1			-23483.892	-29919.324	21564.132	-23523.699	-29959.035	21523.659
	p0.5_CHD	0.0002	0.0002	0.0002	0.0003	1			-23501.490	-29936.842	21545.971	-23506.365	-29941.782	21541.552
	p1_CHD	0.0003	0.0003	0.0003	0.0004	1			-23503.346	-29938.680	21543.986	-23504.511	-29939.946	21543.535
	p2_CHD	0.0004	0.0004	0.0003	0.0005	1			-23503.383	-29938.782	21544.415	-23504.474	-29939.843	21543.107
	p3_CHD	0.0004	0.0004	0.0003	0.0005	1			-23503.466	-29938.864	21544.324	-23504.391	-29939.761	21543.197
	p0.5_Stroke	0.0011	0.0011	0.0009	0.0013	1			-23493.567	-29930.308	21563.625	-23514.235	-29948.268	21523.994
	p1_Stroke	0.0020	0.0020	0.0016	0.0024	1			-23501.845	-29938.202	21552.657	-23506.000	-29940.415	21534.905
	p2_Stroke	0.0035	0.0035	0.0028	0.0042	1			-23500.427	-29936.869	21554.664	-23507.382	-29941.720	21532.984
	p3_Stroke	0.0035	0.0035	0.0028	0.0042	1			-23500.372	-29936.186	21550.326	-23507.433	-29942.393	21537.286
	p0.5_ESRD	0.0074	0.0074	0.0059	0.0089	1			-21873.128	-28357.867	23520.051	-25065.655	-31453.569	19649.747
	p1_ESRD	0.0152	0.0152	0.0121	0.0182	1			-22052.313	-28535.527	23330.185	-24892.206	-31281.708	19834.309
	p2_ESRD	0.0078	0.0078	0.0063	0.0094	1			-23185.749	-29631.844	21936.916	-23813.535	-30238.468	21161.000
	p3_ESRD	0.0017	0.0017	0.0014	0.0020	1			-23478.777	-29914.956	21574.477	-23528.900	-29963.494	21513.259
	pCVD_Death	0.0049	0.0049	0.0039	0.0058	1			-23536.044	-29974.392	21532.388	-23472.447	-29904.927	21554.908
	pCHD_Death	0.0007	0.0007	0.0006	0.0009	1			-23504.343	-29939.792	21543.801	-23503.516	-29938.835	21543.720
	pESRD_Death	0.0552	0.0552	0.0442	0.0662	1			-25824.295	-32324.591	19677.776	-21568.987	-27950.241	23099.796
	pStroke_Death	0.0040	0.0040	0.0032	0.0048	1			-23517.042	-29954.329	21543.968	-23491.027	-29924.539	21543.556
	cphuman	2210.1119	2210.1119	1653.2000	2767.0300	1			-23483.341	-29939.313	21708.466	-23524.517	-29939.313	21379.053
	cshuman	156257.8100	156257.8100	100884.0000	211631.5500	1			-23503.929	-27599.789	5167.090	-23503.929	-32278.834	37920.411
	cdhuman	76234.5296	76234.5296	16087.0000	136381.8900	1			-25727.490	-32480.525	21543.760	-21280.374	-27398.108	21543.760
	cpprogram	94.0000	94.0000	0.0000	354.5500	1			-23500.454	-29939.313	21571.561	-23513.561	-29939.313	21466.703
	csprogram	8163.9028	8163.9028	1308.1000	15019.7500	1			-23503.929	-29649.658	19516.173	-23503.929	-30228.970	23571.361
	cdproagram	4466.1765	4466.1765	4143.7000	4788.6100	1			-23515.850	-29952.938	21543.760	-23492.009	-29925.690	21543.760
	cprehy	898.4040	898.4040	718.7232	1078.0848	1			-23269.783	-29705.167	21777.907	-23738.075	-30173.459	21309.614
	chy1	963.4320	963.4320	770.7456	1156.1184	1			-23394.075	-29829.459	21653.614	-23613.783	-30049.167	21433.906
	chy2	1194.6120	1194.6120	955.6896	1433.5344	1			-23445.957	-29881.341	21601.732	-23561.901	-29997.285	21485.788
	chy3	2989.4760	2989.4760	2391.5808	3587.3712	1			-23451.385	-29886.769	21596.304	-23556.473	-29991.857	21491.217
	cMI	31628.7790	31628.7790	25303.0232	37954.5348	1			-23128.872	-29564.256	21918.817	-23878.985	-30314.370	21168.704
	cCHF	11202.4460	11202.4460	8961.9568	13442.9352	1			-23483.921	-29919.305	21563.768	-23523.936	-29959.321	21523.753
	cStroke	12609.6855	12609.6855	10087.7484	15131.6226	1			-23371.928	-29807.312	21675.762	-23635.930	-30071.314	21411.759
	cESRD	123923.1180	123923.1180	99138.4944	148707.7416	1			-19205.085	-25640.469	25842.605	-27802.773	-34238.157	17244.916
	U_nor	0.6984	0.6984	0.4689	0.9278	1			-23503.929	-29939.313	21543.760	-23503.929	-29939.313	21543.760
	U_pre	0.6514	0.6514	0.6066	0.6962	1			-22208.142	-28288.740	20356.039	-24962.024	-31796.635	22880.254
	U_1	0.6553	0.6553	0.6057	0.7048	1			-22858.199	-29116.782	20951.883	-24187.202	-30809.667	22170.050
	U_2	0.6830	0.6830	0.5595	0.8065	1			-22820.103	-29068.256	20916.964	-24230.004	-30864.187	22209.282
	U_3	0.6690	0.6690	0.5153	0.8227	1			-23190.685	-29640.302	21256.640	-23825.751	-30349.250	21838.743
	U_MI	0.6840	0.6840	0.6165	0.7524	1			-23410.239	-29819.970	21457.884	-23599.636	-30061.225	21631.486
	U_Stroke	0.6050	0.6050	0.5445	0.6655	1			-23429.735	-29844.805	21475.754	-23578.594	-30034.421	21612.199
	U_CHF	0.6400	0.6400	0.6086	0.6714	1			-23497.340	-29930.920	21537.721	-23510.521	-29947.710	21549.803
	U_ESRD	0.6000	0.6000	0.3900	0.8100	1			-22677.907	-28887.126	20786.627	-24392.400	-31071.048	22358.135

-3760.6288	From ESRD to death	-4,131.89	-3,451.04
	Human costs	-4,116.40	-3,404.86
	Discounting rate	-4,241.92	-3,370.23
	[illegible]	[illegible]	[illegible]
	[illegible]	[illegible]	[illegible]

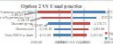

[illegible]	[illegible]	[illegible]	[illegible]
	Human costs	-5,196.88	-4,383.70
	Discounting rate	-5,525.62	-4,367.66
	Proportion of hypertension level 1	-4,190.62	-5,502.23
	Treatment cost of ESRD	-4,102.47	-5,478.11

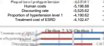

3447.0016			
	From ESRD to death	3,148.44	3,695.97
	From hypertension level 1 to ESRD	3,732.83	3,173.49
	Project costs	3,122.59	3,771.42
	Treatment cost of ESRD	4,134.82	2,759.19
	[illegible]	[illegible]	[illegible]

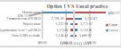

Supplement: Supplementary file 1 [file Data_Sheet_1.PDF]
